# Microbial Hub Taxa Link Host and Abiotic Factors to Plant Microbiome Variation

**DOI:** 10.1371/journal.pbio.1002352

**Published:** 2016-01-20

**Authors:** Matthew T. Agler, Jonas Ruhe, Samuel Kroll, Constanze Morhenn, Sang-Tae Kim, Detlef Weigel, Eric M. Kemen

**Affiliations:** 1 Max Planck Institute for Plant Breeding Research, Cologne, Germany; 2 Center for Genome Engineering, Institute for Basic Science, Daejeon, South Korea; 3 Max Planck Institute for Developmental Biology, Tübingen, Germany; Harvard University, UNITED STATES

## Abstract

Plant-associated microorganisms have been shown to critically affect host physiology and performance, suggesting that evolution and ecology of plants and animals can only be understood in a holobiont (host and its associated organisms) context. Host-associated microbial community structures are affected by abiotic and host factors, and increased attention is given to the role of the microbiome in interactions such as pathogen inhibition. However, little is known about how these factors act on the microbial community, and especially what role microbe–microbe interaction dynamics play. We have begun to address this knowledge gap for phyllosphere microbiomes of plants by simultaneously studying three major groups of *Arabidopsis thaliana* symbionts (bacteria, fungi and oomycetes) using a systems biology approach. We evaluated multiple potential factors of microbial community control: we sampled various wild *A*. *thaliana* populations at different times, performed field plantings with different host genotypes, and implemented successive host colonization experiments under lab conditions where abiotic factors, host genotype, and pathogen colonization was manipulated. Our results indicate that both abiotic factors and host genotype interact to affect plant colonization by all three groups of microbes. Considering microbe–microbe interactions, however, uncovered a network of interkingdom interactions with significant contributions to community structure. As in other scale-free networks, a small number of taxa, which we call microbial “hubs,” are strongly interconnected and have a severe effect on communities. By documenting these microbe–microbe interactions, we uncover an important mechanism explaining how abiotic factors and host genotypic signatures control microbial communities. In short, they act directly on “hub” microbes, which, via microbe–microbe interactions, transmit the effects to the microbial community. We analyzed two “hub” microbes (the obligate biotrophic oomycete pathogen *Albugo* and the basidiomycete yeast fungus *Dioszegia*) more closely. *Albugo* had strong effects on epiphytic and endophytic bacterial colonization. Specifically, alpha diversity decreased and beta diversity stabilized in the presence of *Albugo* infection, whereas they otherwise varied between plants. *Dioszegia*, on the other hand, provided evidence for direct hub interaction with phyllosphere bacteria. The identification of microbial “hubs” and their importance in phyllosphere microbiome structuring has crucial implications for plant–pathogen and microbe–microbe research and opens new entry points for ecosystem management and future targeted biocontrol. The revelation that effects can cascade through communities via “hub” microbes is important to understand community structure perturbations in parallel fields including human microbiomes and bioprocesses. In particular, parallels to human microbiome “keystone” pathogens and microbes open new avenues of interdisciplinary research that promise to better our understanding of functions of host-associated microbiomes.

## Abstract 2

Microbial interactions between kingdoms are responsible for significant microbiome variation on the surface of plants. Highly connected microbes are most important, amplifying abiotic and host factors to cause large perturbations in the structure of microbial communities.

## Author Summary

Under natural conditions, plant growth and behavior strongly depend on associated microbial communities called the microbiome. Much research has been performed to evaluate how the environment and plant genes help to determine the structure of the microbiome. Here, we show that interactions between microorganisms on plants can be responsible for large portions of observed microbial community structures on leaves. Importantly, particular microbes, termed “hub microbes” due to their central position in a microbial network, are disproportionally important in shaping microbial communities on plant hosts. We discovered fungal and oomycete hub microbes that act by suppressing the growth and diversity of other microbes—even across kingdoms—and several candidate bacterial hubs, which largely positively control the abundance of other bacteria. We also showed that factors impacting the microbial community—such as plant genotype—are strongest if they affect colonization of a hub microbe because the hub in turn affects colonization by many other microbes. Our results further suggest that hub microbes interact directly or via the microbial community. Hub microbes are thus promising targets for better understanding the effects of host genomic engineering and for future work in controlling disease-associated and beneficial host-associated microbial communities.

## Introduction

Hosts and their associated microbial communities are increasingly seen as inseparable entities (metaorganisms) whose ecology and evolution are inseparably entwined [1,2]. For example, the phyllosphere (above-ground portions) and rhizosphere (below-ground portions) of living plants are niches for myriad microorganisms that can determine the fate of plants by influencing fitness [3] and growth [4,5], protecting from herbivores [6], or driving the evolution of multidisease resistances [7]. Understanding the plant holobiont (the plant and the organisms that live in and on it), therefore, will have immense implications for human food security, biodiversity [8], and ecosystem functionality [9].

Given the broad range of microbes that colonize above-ground parts of plants such as bacteria, yeasts, filamentous fungi [10], and protists [11], there is poor understanding of the entire diversity of those plant-associated microbes as well as factors that shape complex plant microbial communities from host colonization to plant senescence. Current analyses point towards soil [12] and air [13] as important sources of leaf and root microbial inoculum. How defined microbial communities get selected by different plant organs from highly variable and complex inoculum communities [14,15] is under strong debate. Still, since plant phenotypes and fitness depend on the associated microbiome, such knowledge is critical to enable plant microbiome management, that is, reaching the full potential of using microbes and microbial communities to promote beneficial plant–microbe interactions [2,16].

Generally, three mechanisms contribute to microbial community structures: random colonization; species sorting by local factors (e.g., nutrient availability, host availability, and microbial interactions); and isolating factors such as dispersion and distance [17,18]. Previous work has identified neutral, abiotic, and host factors that sort and contribute to differences in plant bacterial or fungal communities [13,19–23]. Such studies are likely to reflect adaptations of microbes that enable them to colonize specific plant host environments [24,25]. While these adaptations can link abiotic and biotic host factors to colonization efficiency, they cannot be understood in isolation, since the host as a holobiont is simultaneously colonized by a multitude of prokaryotes and eukaryotes [26].

Phyllosphere colonization proceeds via mechanisms that fundamentally alter the host, since some microbes participate in what can be described as niche construction. For example, many symbionts (including pathogens) deliver effector proteins to suppress, activate, or alter host defense [27,28], and some are able to completely reshuffle host metabolism [29,30]. These host alterations can cause changes to microbiome structure since some microbes can take advantage of new conditions while others cannot. In fact, the niche of some microbes specifically rely on others. For example, primary colonizers can protect secondary from abiotic selection factors such as desiccation [31] or can increase secondary colonizers’ competitive advantage by providing secondary metabolites [32]. Further examples of direct microbe–microbe interactions include hyperparasitism of primary colonizers [33] and opportunists that exploit a weakening of plant defenses to colonize their hosts [34,35]. Such interactions explain why certain colonizers can affect establishment of even distantly related microbes on the host [32,36] and suggest an important role for interactions in determining microbiome structures.

Most studies implicitly assume that abiotic and host factors differentiate microbial communities because of variable microbial adaptations. Research in the animal field has shown that for example, variation in the Major Histocompatibility class II (MHC) genotypes contributes to microbial variation among hosts [37]. In human populations, the gut microbiome is significantly influenced by the host genetics and in turn, the microbiome has a significant impact on host metabolism [38]. How microbe–microbe interactions fit in colonization models remains, however, largely unknown, not least because of limitations to the robustness and depth of taxonomic resolution. To begin to move towards a more holistic understanding of forces shaping microbiomes in general and the phyllosphere microbiome in particular, we have measured diversity and community composition of three major groups of microbes representing key branches of life (fungi, bacteria, and oomycetes as a representative of the heterogeneous group of protists) in both epiphyte (surface microbe) and endophyte (interior microbe) leaf compartments of individual samples. Complementary approaches of wild sampling and a common garden experiment confirmed combinatorial mechanisms of species isolation and sorting due to abiotic and host factors that manipulate *A*. *thaliana* phyllosphere microbiomes. A systems biology approach documented highly interactive “hub” microbes, and in controlled laboratory experiments we confirmed that one, *Albugo laibachii*, strongly affects phyllosphere communities and found evidence for direct interactions by a second, *Dioszegia* sp. The results demonstrate that hub microbes mediate between sorting factors and microbial colonization, effectively amplifying sorting effects in the phyllosphere and stabilizing populations of specific microbes on individual plants. Our findings provide insights into the complexity of multikingdom interactions in the phyllosphere and improve the understanding of the dynamics of plant microbiome colonization.

## Results

### Factors Mediating Phyllosphere Microbiome Assembly

To identify how several factors (Table 1) control phyllosphere microbiome assembly, we selected five sites near Tübingen in southern Germany with stable *A*. *thaliana* populations that have been studied for several years [39] (WH, JUG, PFN, EY, ERG; S1 Table). We collected plants in the fall, covering the early growth phase of *A*. *thaliana* under short day conditions before its resting stage in winter, and in spring, just before its reproductive stage during increasingly longer days (Experiment 1). Microsatellite markers [40] confirmed that there is more *A*. *thaliana* genetic variation between sites than within sites, with no overlap of multilocus haplotypes between sites (S2 Table) [39]. We therefore grouped factors into “sampling time,” which includes differences between fall and spring, and “sampling location,” covering differences between sites such as soil, local climate, and plant genotypes (Table 1). Importantly, a major phenotype observed at all sites except PFN was the presence of white rust caused by the obligate biotrophic oomycete pathogen *Alb*. *laibachii*. From each sample, we recovered epiphytic and endophytic microbes, extracted genomic DNA, and generated six amplicon libraries: two from rRNA gene regions of bacteria (16S rRNA V3/V4 and V5/V6/V7 regions) and two from each of fungi and oomycetes (internal transcribed spacers 1 and 2 [ITS 1 and 2] of the large subunit rRNA complex). We included multiple amplified regions to address the fact that differences arise due to primer specificity and bias and due to differential gene region variability. Therefore, we treated the two amplified regions from a single microbial group complementarily, presenting findings generated by either dataset as well as differences between the datasets. Generally, amplicon-based microbial abundances reported are relative within each gene region.

**Table 1:** Factors tested for their effects on microbial community structure in this study. Since each “tested factor” naturally groups sources of variation together, a list of possible “grouped factors,” which could contribute to observed community structures, is provided.

Experiment	Tested Factor^1^	Example Grouped Factors^2^
Wild Tübingen	Sampling Time	Temperature, host stage
	Location	Soil, climate, host genotype
Cologne Garden	Genotype	Genotype, sub-location
Laboratory	Genotype x *Albugo* sp.	

^1^ “Tested factor” indicates the factor which we measured and which was tested in constrained ordination for effects on the microbial communities.

^2^ “Grouped factors” indicate some factors that are naturally grouped into the tested factor and which likely contribute to observed variation between samples.

**Fig 1:**
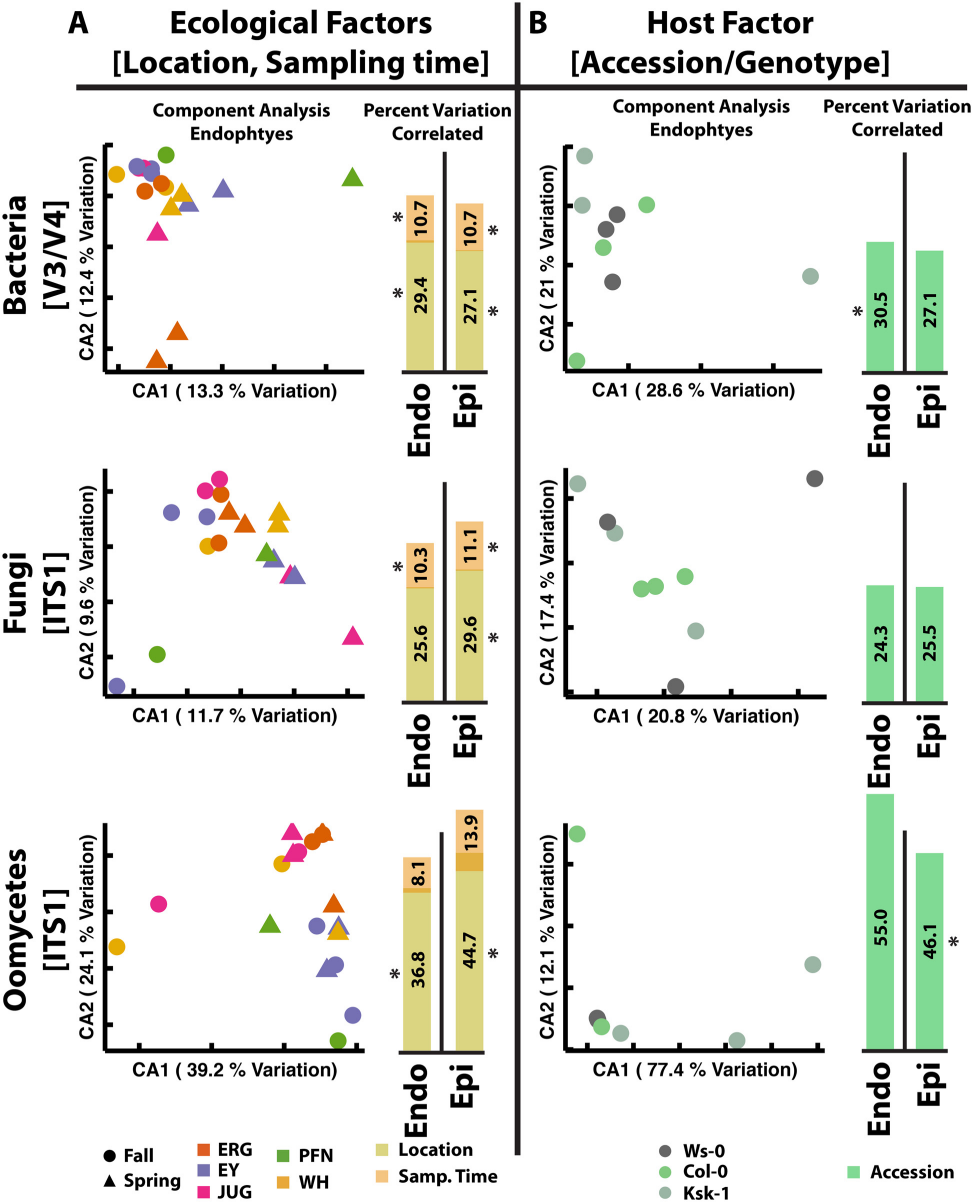
Experiment 1 and 2: Ecological and host factors are important in shaping phyllosphere microbial communities. A. Experiment 1: Sampling location and sampling time correlated to microbial community structure variation observed between Tübingen wild sites. Circles and triangles are samples collected in fall and spring, respectively. Colors of points illustrate the location where the samples were collected. Dot plots are unconstrained endophytic communities, while barcharts show factor correlations to endophytic (endo) and epiphytic (epi) variation. Overlap of bars represents factors correlated to the same variation. B. Experiment 2: The host *A*. *thaliana* accession correlated to microbial community structure variation observed in the Cologne garden experiment. Colors of points represent the host accession. For A and B, figures are based on genus-level data from bacterial 16S V3/V4 region, fungal ITS1 region and oomycete ITS1 region amplicons. For A and B, a star indicates that the measured correlation is statistically significant (*p* < 0.05) based on random permutations of sample classes. (S1_Data.xlsx)

We measured how factors correlated to microbial community structure by performing constrained ordination (canonical correspondence analysis) on log-transformed microbial abundances. For epiphytic and endophytic bacteria and fungi, location was correlated to up to 25%–30% of community variation, and sampling time about 10%–15% (most correlatations are significant at *p* < 0.05 based on random permutations, Fig 1A and S1A Fig). To further clarify variation, we calculated location- and sampling time-specific enrichment of each microbial genus based on whether it was more abundant at a specific sampling site compared to any other site or in spring or fall (Tukey’s honest significant difference test [HSD] *p* < 0.01, i.e., the genus contributes to distinguishing between locations or sampling times). A median of one and four enriched bacterial genera per location (endophytes and epiphytes, respectively) suggests that relatively few species contributed to observed variation between sampling sites (S3 Table). The location PFN, however, was unique because 25 and 16 bacterial genera (endophytes and epiphytes, respectively) were significantly enriched there (S3 Table). Enrichment of many taxa at PFN explains why samples there consistently had some of the highest endophytic and epiphytic bacterial alpha diversities (S2 Fig). Many fungal taxa were enriched in abundance at PFN and JUG (15 and 12 genera, respectively, S3 Table), compared to an average of 2.7 at each of ERG, WH, and EY. Only site PFN had significantly enriched endophytic fungal genera. Generally, bacterial locational variation was more quantitative than fungal: eight abundant fungal genera (each > 500 total observations) were only observed at < 5 sites, while all abundant bacteria were detectable at five or more sites (S3 Fig). Sampling time was also important, with many taxa at higher abundance in fall (122 total taxa compared to 25 in spring) (S4 Table). The large fall/spring difference can mostly be attributed to bacteria: 16 and 14 fungal taxa were more abundant in fall and spring, respectively, while the rest of the enriched taxa were bacteria. Interestingly, while 90 taxa were more abundant in at least one sampling location and 146 at one sampling time, only two taxa were both location- and season-enriched. For both bacteria and fungi, epiphytic alpha diversity was higher than endophytic (S2 Fig), and abundant genera differed between epiphytic and endophytic compartments (S4 and S5 Figs).

Oomycete communities presented a very different picture. Here, while sampling time still was correlated to about 10% of community variation, sampling location was correlated to 35%–80% (depending on leaf compartment and dataset, Fig 1A, S1A Fig). Oomycete alpha diversity was extremely low (S6 Fig) and the obligate biotrophic pathogen *Albugo* was dominant, comprising up to 100% of observations in some samples (S6 Fig), agreeing with observations of extensive white rust symptoms. Overall, we did not observe that sites physically more close to one another (S1 Table) were more similar in terms of observed microbial communities (Fig 1 and S1 and S2 Figs).

Endophytic *Albugo* detected via quantitative polymerase chain reaction (qPCR) in some of the samples scored as white rust-free was easily detectable (S2 Table and S7 Fig), indicating some extent of asymptomatic endophytic *Albugo* growth [11]. Considering the striking symptoms on hosts affected by *Albugo* and its ubiquity, we decided to examine distribution of this organism at the strain level. Two *Albugo* species, *Alb*. *candida* and *Alb*. *laibachii*, have previously been described causing white rust on *A*. *thaliana* [41]. ITS amplicon data suggested absence of *Alb*. *candida* in our samples, and *Alb*. *candida*-specific primers confirmed this (S2 Table). For strain determination, we thus focused on *Alb*. *laibachii*, using newly developed microsatellite-based markers. Although the *Albugo* genus was widespread, we found no strain overlap between sites but instead that each site was dominated by a stable major strain over multiple host generations (S2 Table). The second most common oomycete pathogen in plants was *Hyaloperonospora* sp. (*Hpa*). While only four of 19 tissue samples with observed white rust contained appreciable levels of *Hpa*, we found high relative levels in five of the ten tissue samples where white rust was not observed (more present when white rust was not observed at *p* = 0.022, one-tailed Fischer’s exact test). Hpa-relative abundance was not necessarily dependent on *Albugo*, since some of the highest observed levels were in samples with high levels of measured endophytic *Albugo* (S7 Fig).

Host genotype was not separable from location as a factor in wild samples. To determine whether it could uniquely affect microbial communities, we planted three natural *A*. *thaliana* accessions with differential resistance to *Albugo* sp. strains (Ws-0, Col-0, and Ksk-1: see S8 Fig for qPCR quantification of endophytic *Alb*. *laibachii* Nc14 and *Alb*. *candida* Nc2 levels in susceptible versus resistant accessions) in randomized plots in a common garden in Cologne, Germany (CG) and sampled their microbiomes just before flowering time (Experiment 2). Constrained analysis suggested that plant genotype affected microbial community variation, correlating to 25%–30% of bacterial and fungal community variation and 45%–55% of oomycete community variation (Fig 1B and S1B Fig). Because of the low number of samples, only a few of the correlations were significant, and therefore some of the constrained variance could be due to chance. Therefore, we tested each genus for genotype-enrichment based on whether they were enriched on a specific host accession compared to any other accession (Tukey’s HSD p < 0.05, i.e., the genus contributes to distinguishing the accession from other accessions, S5 Table). Indeed, even with only three samples per accession, multiple bacterial and fungal taxa were detected as enriched on each. The higher correlation of oomycete variation to host genotype was due to the low diversity of oomycete communities in the garden experiment—these were nearly completely dominated by *Alb*. *laibachii* (S6 Fig). We observed significantly more white rust on *A*. *thaliana* accessions Ws-0 and Col-0 than on the partially resistant accession Ksk-1 (S9 Fig), and this agreed with qPCR measurements of endophytic *Albugo* (S2 Table: There was significantly less *Albugo*, using oomycete levels as an *Albugo* proxy, in accession Ksk-1 than in Ws-0 or Col-0 based on one-sided *t* test of 10/5/13 samples at *p* < 0.1 or *p* < 0.05 using both 5/5/13 and 10/5/13 samples). Additionally, we delineated three different *Alb*. *laibachii* strains in the field: the dominant strain 1 was observed on 5 *A*. *thaliana* Col-0 and Ws-0 samples, but only one Ksk-1 sample, strain 2 grew on one sample each of *A*. *thaliana* Col-0 and Ws-0, while a third strain was found in a second *A*. *thaliana* Ksk-1 sample only (S9 Fig). Taken together, our results based on the phyllosphere of the model host *A*. *thaliana* indicate that the factor’s location, sampling time, and host genotype are important determinants for plant colonization patterns of bacteria, fungi, and oomycetes.

### Interkingdom Connectivity of Phyllosphere Microbiomes

**Fig 2:**
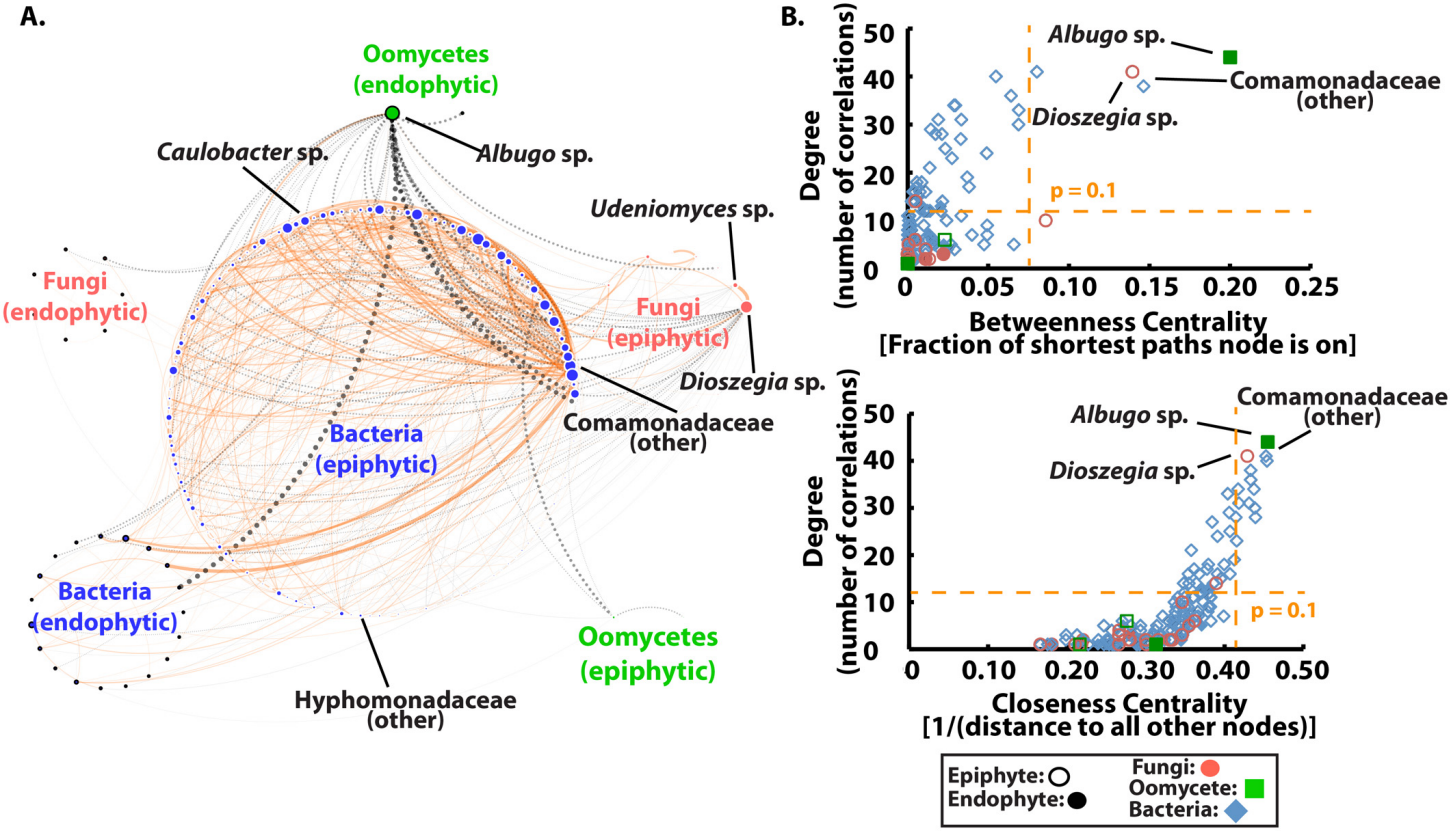
Computational Experiment 3: Inter- and intra-kingdom microbe–microbe interactions affect phyllosphere microbiome structure. A. A correlation network demonstrates that correlations between microbes within kingdoms tend to be positive (orange solid), while correlations between kingdoms tend to be negative (black dashed). Boldness of lines is related to the strength of the correlation. Correlations were made using samples from both Experiment 1 and Experiment 2. Additional care was taken to ensure correlations were robust (see S1 Text). The network structure was typical of a scale-free network since only a few nodes were highly connected (a power-law fit to the node degree distribution has alpha = −1.072 and r^2^ = 0.846). B. “Hub” microbes were identified as those which were significantly more central based on all three measurements of centrality. For the network shown in A (based on one of several cutoffs for “good” correlations, see S1 Text), three microbes, *Albugo* sp., *Dioszegia* sp., and a genus of *Comamonadaceae* were identified as “hubs” (yellow line: *p* = 0.1 based on a log-normal distribution fit). Other genus-level hub microbes indicated in Fig 2A were identified by combining the results of several other correlation cutoffs (see S11 Fig and S7 Table). (S1_Data.xlsx)

Up to ~40% of observed phyllosphere microbial community variation in constrained ordination models of wild samples could be explained by location and sampling time together (Fig 1 and S1 Fig). We hypothesized that microbe–microbe interactions could contribute to the remaining variation and reasoned that the most important microbes and microbial relationships could be discovered by looking for “hubs”—highly connected microorganisms in scale-free correlation networks [22,42]. Therefore, we generated a co-occurrence network by measuring abundance correlations between 90,524 pairs of microbes grouped at the genus level (Computational Experiment 3, Fig 2A—important terms related to network analysis are defined in Box 1). Correlations were based on samples from Experiment 1 and Experiment 2. We did not seek to detect binary interactions (where the presence of one microbe depends on another regardless of abundance), which would be distorted, because even single leaf samples pool leaf areas that are very large and diverse in terms of microbial habitats [43]. Using a cutoff that removed correlations with either a low r-squared value or that were based on microbes found only in limited samples (see S1 Text for details), the resulting edges represented correlations that are widespread among locations we sampled, since most (86%) were supported in at least 50 of 100 randomly subsampled datasets (S10 Fig). Within kingdoms, we found that correlations were usually positive (86.5%, *n* = 630) and were dominated by interactions between bacteria. Correlations between microbes from different kingdoms were overwhelmingly negative (76.6%, *n* = 141), driven by a disproportionate number of correlations to a few microbes (relatively more interactions with oomycetes than random X^2^ = 169.4 *p* < 2.2 x 10^−16^, S6 Table).

**Box 1. Definitions and clarifications of important terms related to network analyses that are commonly used in this manuscript.**

**Node**—In the network analysis, a node is a taxa representing operational taxonomic units (OTUs) grouped at a specific level (e.g., genus level).

**Edge**—In the network analysis, edges are lines connecting **nodes** and represent correlations between the nodes.

**Connectedness/Connectivity**—How central a node is in the network, i.e., how well connected it is to the rest of the network, measured by node parameters **degree, betweenness centrality,** and **closeness centrality**.

**Degree**—The number of direct correlations to a node in the network.

**Betweenness centrality**—The fraction of cases in which a node lies on the shortest path between all pairs of other nodes.

**Closeness centrality**—The reciprocal of the sum of distances to all other nodes.

**Hub node**—A node which is significantly more connected within the network than other nodes according to all three node parameters.

**Edge node**—Poorly connected nodes within the network that likely have little influence on microbial community structure.

**Keystone node**—A hub node that fundamentally underlies the observed network structure. Without this node, the observed network would look significantly different.

**Keystone versus Hub nodes**—Hub species are logical places to look for ecologically important microbes in a community. Ecologically important species are responsible for the microbial community structure and are therefore keystone species—without them the dynamics of the community changes. Not all hub species are keystones, however, since a high number of direct interactions (a requirement of hubs) is not a requirement of keystone species. Keystones are rather defined by the quantity of overall interactions in the network that are dependent on them [44]. Thus, some hubs that are only important in their “neighborhood” of the network would not be keystones overall. In the context of this study, a keystone microbe would be a critical determinant of colonization of widely occurring microbial taxa, and nonkeystone hub would be important for determining colonization of some specific taxa but not overall.

We found that the cutoff used to identify “good” correlations could strongly affect identification of the most-connected microbes (S11 Fig). Therefore, we used several cutoffs to identify genera with significantly (*p* < 0.1 based on fitting a log-normal distribution) higher betweenness centrality, closeness centrality, or degree, all of which are measures of how connected a node is in the network (defined in Box 1). Taking the intersection of significant taxa from the three connectivity parameters, genera representing each kingdom (*Albugo* sp., *Udeniomyces* sp., *Dioszegia* sp., *Caulobacter* sp., a genus of *Comamonadaceae* and a genus of *Burkholderiales*, the last two of which could not be identified at the genus level) were highly connected (S11 Fig). We performed the same analysis on operational taxonomic units (OTUs) grouped at order, family, and species levels, and all genera except *Udeniomyces* sp. were supported at > 1 taxonomic level (S7 Table). *Albugo* sp. was supported at all tested taxonomic levels.

**Fig 3:**
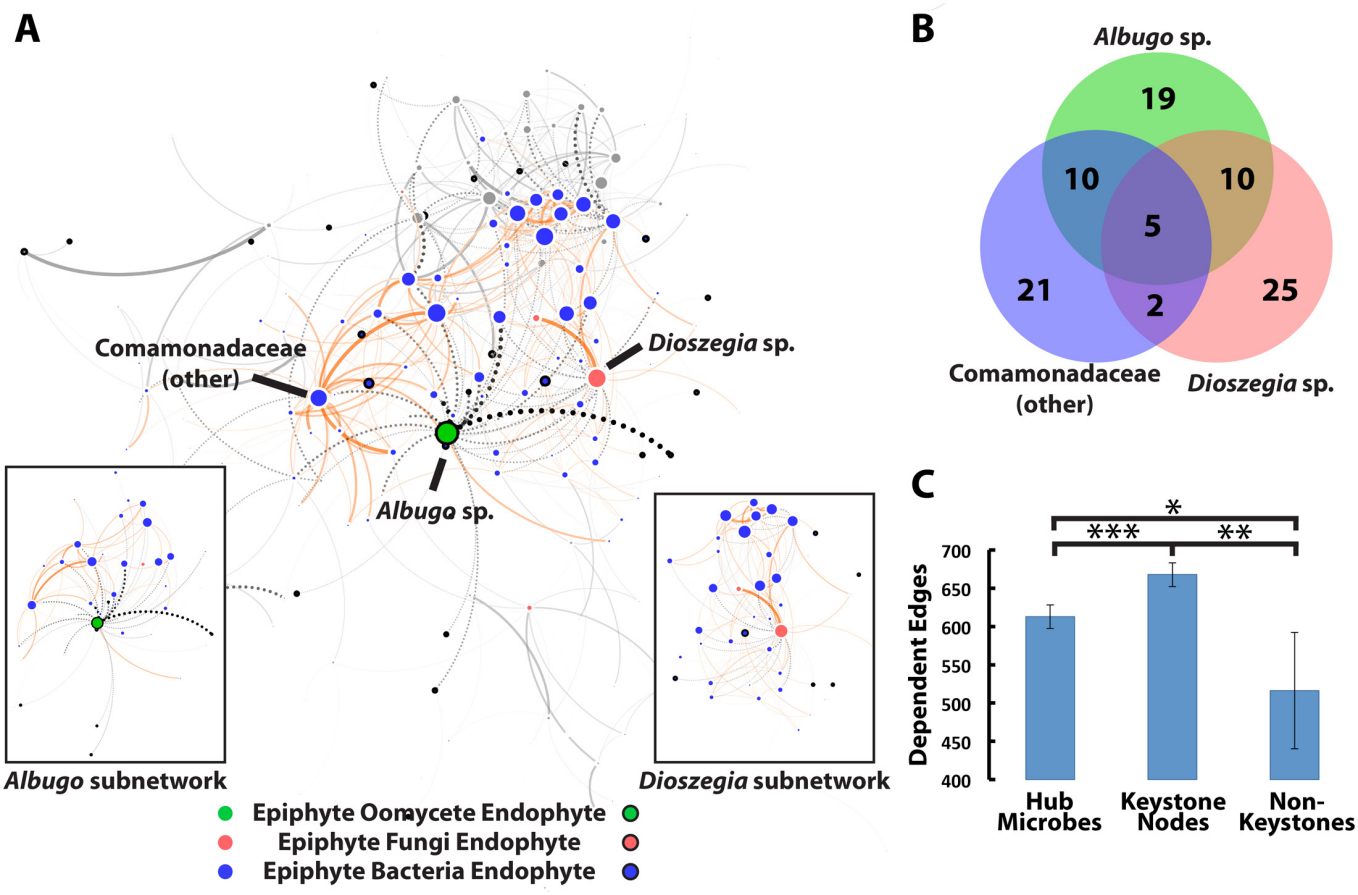
Computational Experiment 3: Hub microorganisms are critical determinants of the microbiome interaction network structure. A. Most high-degree bacteria (including the genus of *Comamonadaceae* designated as a hub) are first neighbors (i.e., direct and negative correlates) of the hub microbial genera *Albugo* sp. and *Dioszegia* sp., and many group into an intercorrelated cluster. First neighbors of the three “hub” microbes are shown in color and the rest of the network is shown in greyscale. The depiction is a spring-loaded visualization of the network in Fig 2 where tightly correlated nodes cluster together. B. The hub microbes were partly independent, since about half of the nodes to which they correlated were unique and half were shared. They together directly reach over half (100/191) of all nodes in the network. C. Hub microbes (high degree organisms with high centrality) can be considered as reasonable keystone species, since the magnitude of their effects in the network extend over more edges than nonkeystone nodes (high abundance organisms with low degree and low centrality) but over fewer than keystone nodes (high degree organisms with low centrality). An edge was considered dependent if it was not observed in a network built using partial correlations controlling for abundance of the test microbes. Error bars show standard deviation, and significance was tested with a one-sided Welch’s *t* test where (*): *p* < 0.1, (**): *p* < 0.05 and (***): *p* < 0.01. Hub nodes: *Albugo* sp., *Dioszegia* sp. and a genus of *Comamonadaceae*. Keystone nodes: *Mycobacterium* sp., *Rhodoplanes* sp., and *Rhizobiales* (other). Nonkeystone nodes: *Pseudomonas* sp., *Oxalobacteriaceae* (other), and *Sphingomonas* sp. (S1_Data.xlsx)

Hub microbes are not necessarily keystones in the community, or taxa which are responsible for significant amounts of the observed microbial community network structure [44], but ecologically relevant hub microbes are likely to be. To check if our analysis identified keystone hubs, we computationally analyzed three of the “hub” microbes (*Dioszegia* sp., *Albugo* sp., and the *Comamonadaceae* genus) identified in the genus-level network (Fig 2A). Together, these three hub microbes are direct correlates of most other nodes in the network (100 of 191 nodes, Fig 3B). We first generated a “spring-loaded” network view in which tightly correlated microbes form clusters (Fig 3A). The main observed cluster was of high-degree epiphytic bacteria and was formed by the large number of positive correlations between them. Interestingly, most microbes with high degree were first neighbors of the three hubs and were negatively correlated to *Albugo* sp. or *Dioszegia* sp. (Fig 3A), suggesting these microbes could be responsible for observed high positive correlations between many epiphytic bacteria. Next, we computationally “removed” each of the hub microbes to test their influence on network structure by building networks with partial correlations that account for their abundances. We also tested positive control keystone genera (with high degree and low betweenness centrality) or negative control species (with high abundance, low degree, and low centrality) (Fig 3C). Hub microbes affected less of the network structure than positive controls, but more edges were dependent on them than on negative controls. We can conclude from this that our three main “hub” microbes are likely “keystone” species with an important role in determining network structure for the leaf microbial community.

### Experimental Testing of the Microbial Hub Genera *Albugo* and *Dioszegia*

The hub microbes *Albugo* and *Dioszegia* were strongly negatively correlated to many of the bacteria in the microbial community networks but are themselves affected by abiotic and host factors. For example, *Albugo* is affected by host resistance encoded by single *A*. *thaliana* genes [45], and *Dioszegia*, although widespread, was seasonal, being significantly more abundant in spring samples (S4 Table). In light of the effects of abiotic and host factors on microbial community structure and the presence of central hubs in the microbial network, we hypothesized that there is a specific mechanism whereby microbial hubs act as “receptors” of abiotic and host factors and “regulatory units,” amplifying or dampening effects of microbiome perturbations. To test our hypothesis, we examined the effect of the presence of isolates of *Albugo* (Experiment 4) and *Dioszegia* (Experiment 5) on other phyllosphere microbiota.

Axenic isolation of *Albugo* is difficult because of its obligate biotrophic lifestyle, but several characteristics make this a good model system for testing our hypothesis. First, *Albugo* is easily propagated with associated microbes that would also be propagated in nature (i.e., spore- or leaf-associated microbes) by washing infected leaves (where infection refers to susceptible plants treated with live *Albugo* spores and visible white rust) and reinoculating. Second, its presence is easily controlled independently of other microbes by introducing a resistant host. We selected two strains (*Alb*. *laibachii* Nc14 and *Alb*. *candida* Nc2) that had been propagated continually for > 1 yr, giving any associated microbiome time to acclimate to lab conditions. We individually inoculated both strains using leaf washes (containing the *Albugo* strain spores and the strain-associated microbial community) onto the three host accessions that we had used for the garden experiment. As expected, susceptible plants displayed strong symptoms, while resistant plants were asymptomatic and had very low detected levels of *Albugo* sp. (S8 Fig). To additionally simulate an abiotic factor limiting *Albugo* (e.g., a distribution limitiation in the wild), we included a second set of *Albugo*-free controls by removing *Albugo* spores from leaf washes by filtering (< 6 μm). This set of controls also allowed us to account for noise in controls due to host genotype background. Filtering could have affected the abundance of other microbes, so we confirmed observed trends in one replicate of an experiment in which *Albugo* filtering was replaced by inactivation using a combination of the oomycete inhibitors metalaxyl and benalaxyl. In all cases all tested conditions were grown together in growth chambers and the communities were allowed to adapt over two cycles of reinoculation before sample collection for microbial community profiling (S12 Fig).

**Fig 4:**
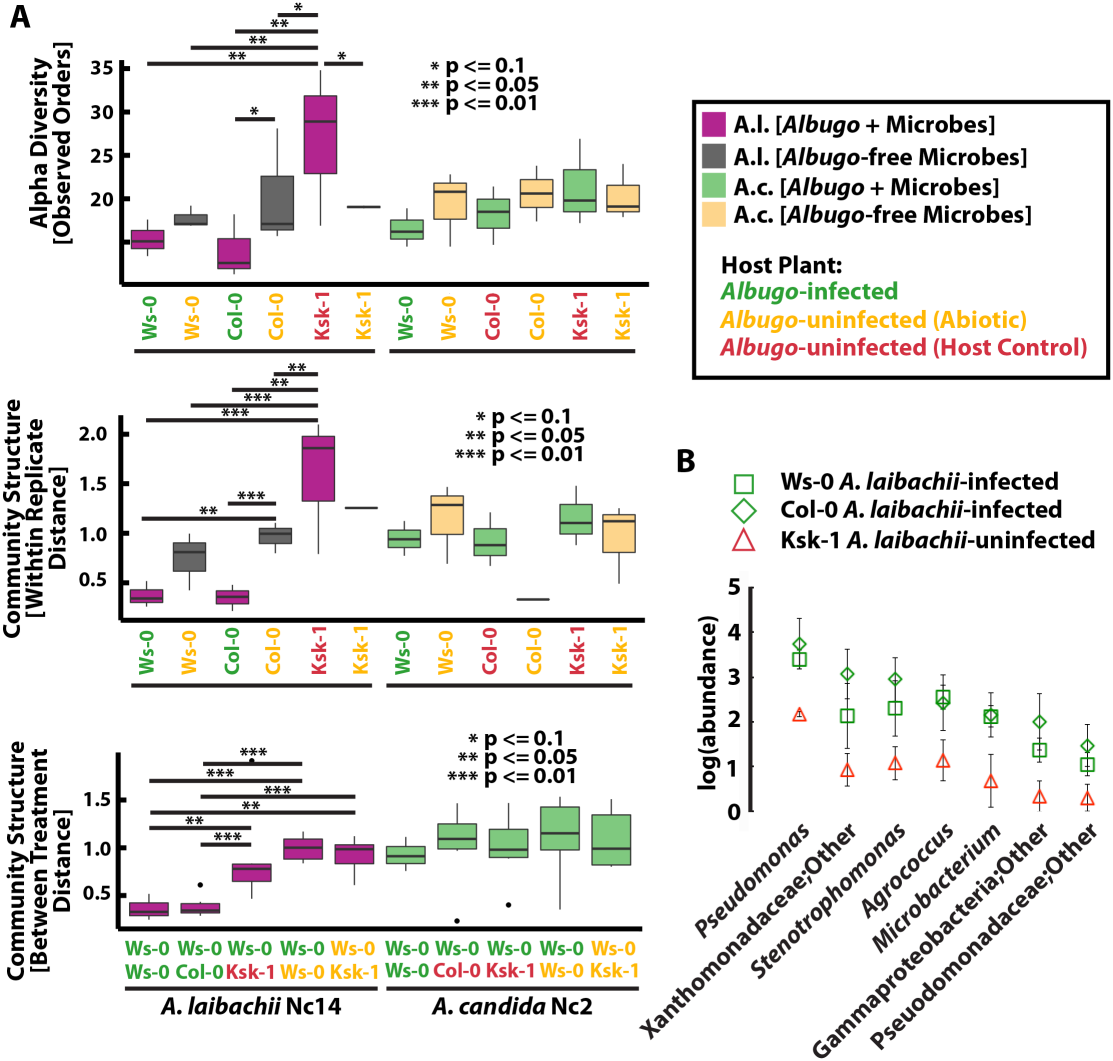
Experiment 4: Species of the obligate biotrophic pathogen and hub genus *Albugo* can affect colonization of microbes in the phyllosphere, linking abiotic or host genotype factors to a mechanism for observed microbial community variation. A. When *Alb*. *laibachii* Nc14 or *Alb*. *candida* Nc2 are absent due to abiotic (physical spore removal) or host (resistance) factors, the pathogen-associated microbial community increases in alpha diversity (also see S12 Fig) is less replicable (*A*. *laibachii* only*)* and shifts significantly (*A*. *laibachii* only*)*. B. Several genera of bacteria were observed to more efficiently colonize the endophytic compartment of the phyllsophere in plants infected with *Albugo* sp. than in controls. For A and B: Ws-0: *A*. *thaliana* Ws-0, Col-0: *A*. *thaliana* Col-0, Ksk-1: *A*. *thaliana* Ksk-1. Green: Susceptible hosts, Red: Resistant hosts, Yellow: Filter removal of *Albugo* on all hosts. (S1_Data.xlsx)

If *Albugo* is indeed a “hub” that transmits, e.g., host factors, affecting colonization of many microbes, we expected the following: 1) decreased alpha diversity as a consequence of infection (following from the observed strong negative correlations to many bacterial taxa), 2) less variability between replicates of infected plants (since other microbes in the inoculum were cocultivated with *Albugo* and many are presumably reliant on its presence), 3) divergence of control from infected communities, and 4) stronger differences between genotypes in the presence of *Albugo*. Compared to the resistant host *A*. *thaliana* Ksk-1, epiphytic bacterial communities on *Alb*. *laibachii*-infected plants had significantly lower alpha diversity (Fig 4A, S13 and S14 Figs) and significantly more similar beta diversity between replicates (within-replicate distance, Fig 4A and S15 Fig). Bacterial communities on plants with *Alb*. *laibachii* infection were more similar to each other than to uninfected controls, although this effect was mostly apparent in the bacterial V3/V4 dataset (between-treatment distance, Fig 4A and S16 Fig). Effects with abiotic *A*. *laibachii* control (regardless of filter removal or chemical inhibition) were the same but less significant than due to host resistance (filtering [Fig 4A and S13, S15 and S16 Figs]/chemical inhibition: [S14 Fig]—significance for within-replicate and between-treatment distances were stronger for the V3/V4 dataset). Alpha diversity or within-replicate distance differences between the three *A*. *thaliana* accessions were strongly increased in the presence of active *Alb*. *laibachii* (S13, S14 and S15 Figs), confirming that *Alb*. *laibachii* can amplify host genotype-specific bacterial community differences. The effect of *Alb*. *laibachii* on fungal communities were less consistent and not as clear. Most apparent was a slight depression in fungal alpha diversity with infection, but without statistical significance (Fig 4A and S13–S16 Figs).

For *Alb*. *candida* infections, a similar and consistent trend of relatively low bacterial and fungal alpha diversity was observed on infected *A*. *thaliana* Ws-0 (Fig 4A, S13 and S14 Figs), but it was not significant. We also did not observe more similar bacterial or fungal communities between replicates or between treatments with *Alb*. *candida* infection (Fig 4A, S15 and S16 Figs). These results, combined with much higher numbers of epiphytic bacteria on *Alb*. *candida*-infected leaves than *Alb*. *laibachii* (S17 Fig) suggests a comparatively weak impact on the bacterial community in *A*. *thaliana* caused by *Alb*. *candida* infection.

Not only epiphyte communities were disturbed by *Albugo* infection. Numbers of endophytic bacterial or fungal reads (which have been used as a proxy for the amount of endophyte microbes [22]) were lower in the absence and higher in the presence of *Albugo* (S18 Fig). We identified a subset of bacteria that were significantly enriched as endophytes during infection but not in any control, and most of them only due to *Alb*. *laibachii* infection, indicating that *Albugo* enabled their colonization of endophytic space (Fig 4B and S18 Fig). These bacteria were not likely to simply have been found because of higher epiphyte numbers in infected plants, since not all abundant taxa were enriched during infection (S19 Fig).

Next, we tested the hub fungus *Dioszegia* sp., which we isolated from the endophytic compartment of *A*. *thaliana* at site EY. Unlike *Albugo*, *Dioszegia* can be axenically cultivated, making possible tests of direct, one-on-one interactions with other microbes in the phyllosphere. In short, we spray-inoculated 3-wk old axenically-grown *A*. *thaliana* seedlings with *Dioszegia* sp. After 3 d, we inoculated isolates of one of six bacterial genera (all of which were isolated on or near *A*. *thaliana* and which we observed in phyllosphere samples, S8 Table). Colony forming units (CFUs) of *Dioszegia* and the bacteria were counted at the starting time and after one week of coculture (S20A and S20B Figs). For four isolates (*Janthinobacterium*, *Caulobacter*, *Flavobacterium*, and *Agromyces*), negative correlations to *Dioszegia* had been observed in the network analysis, while for two isolates (*Pseudomonas* and *Rhodococcus*) we had observed no correlation (S20C Fig). Of the latter two, only *Rhodococcus*, an isolate from *Alb*. *laibachii* Nc14 spores, interacted by reducing *Dioszegia* growth. *Rhodococcus* generally grew to high epiphytic abundances in lab conditions (S21 Fig), and thus reduced growth was probably due to spatial competition. Of the other four isolates, *Janthinobacterium* did not survive on the leaf and we did not observe any effect of *Flavobacterium*. *Agromyces* caused slightly reduced *Dioszegia* growth, but itself grew poorly in the phyllosphere. Of the negatively correlating genera, the *Caulobacter* isolate grew the best alone on plants and was strongly inhibited by *Dioszegia* (~100-fold lower CFU counts). *Caulobacter* was also identified as a hub at the genus and species level (S7 Table).

Taken together, our findings confirm that the microbial “hub” *Albugo* is a strong interactor in the phyllosphere, and that its presence limits alpha diversity and affects plant microbial communities. They also support the hypothesis that *Albugo* could stabilize plant microbial communities, for example on hosts in a single wild population. Negative correlations between the fungal hub *Dioszegia* and bacteria in the phyllosphere are due to both antagonistic effects by other bacteria on *Dioszegia* (e.g., due to spatial competition) and direct antagonism on specific bacteria. Therefore, host or abiotic signatures that affect the abundance of the hubs *Albugo* and *Dioszegia* can also have disproportionately large effects among phyllosphere microbiota.

### Microbial Hubs Mediate between Abiotic and Host Effects and Observed Phyllosphere Diversity

To look closer at the mechanism of how hub microbes select phyllosphere microbiota, we asked which endophytic taxa were enriched in the field in samples with high measurable levels of endophytic *Albugo* sp., and whether these were also enriched in lab experiments. Generally, in wild samples, no single bacterial genus dominated endophytes. Several taxa identified at the genus level were enriched (>10% of reads) in individual samples, including *Pseudomonas* (up to 93% of reads at many sites), *Sphingomonas* (up to 29% of reads at Cologne, ERG, and EY), *Methylobacterium* (up to 22% of reads at Cologne and PFN), *Deinococcus* (up to 12% of reads at Cologne), and *Flavobacteria* (10% of reads JUG) (S4 Fig). Of these, *Pseudomonas* sp. was also the genus that colonized the endophytic compartment during *Alb*. *laibachii* Nc14 infection most efficiently (Fig 4B). Therefore, while specific bacterial genera seem to benefit from *Albugo* infection, these seem to be location-specific rather than *Albugo*-specific.

Interestingly, we calculated interkingdom correlations of microbes to alpha diversity indices and found that the hub taxa *Albugo* and *Dioszegia* are strongly negatively correlated overall to bacterial diversity, as are two other epiphytic yeast-like fungal genera (*Leucosporidiella* and *Udeniomyces*—the latter was identified as a hub at the genus level, S7 Table) and a genus of *Pleosporales* fungi (S22 Fig). Only the epiphytic fungal genus *Heterobasidion* was positively correlated with bacterial diversity based on support from both bacterial amplicon datasets; in addition, one dataset supported positive correlations for several other fungal classes and the genus *Aspergillus*. Additionally, several epiphytic bacterial classes positively correlated with fungal epiphyte diversity and endophytic *Pseudomonas* negatively correlated to it (S22 Fig). Negative correlations of hubs to bacterial diversity (also observed in lab experiments for *Albugo* sp.) correspond to the network observation of extensive negative correlations to epiphytic bacterial genera. Thus, as hubs, *Albugo* and *Dioszegia* decrease bacterial diversity and thereby increase relative abundances of a few groups of abundant and location-specific bacteria. Significant correlations of other genera to alpha diversity of bacteria and fungi suggest that other bacterial and fungal taxa are also important and will be detected as hubs with different sampling strategies (e.g., within single host populations).

**Fig 5:**
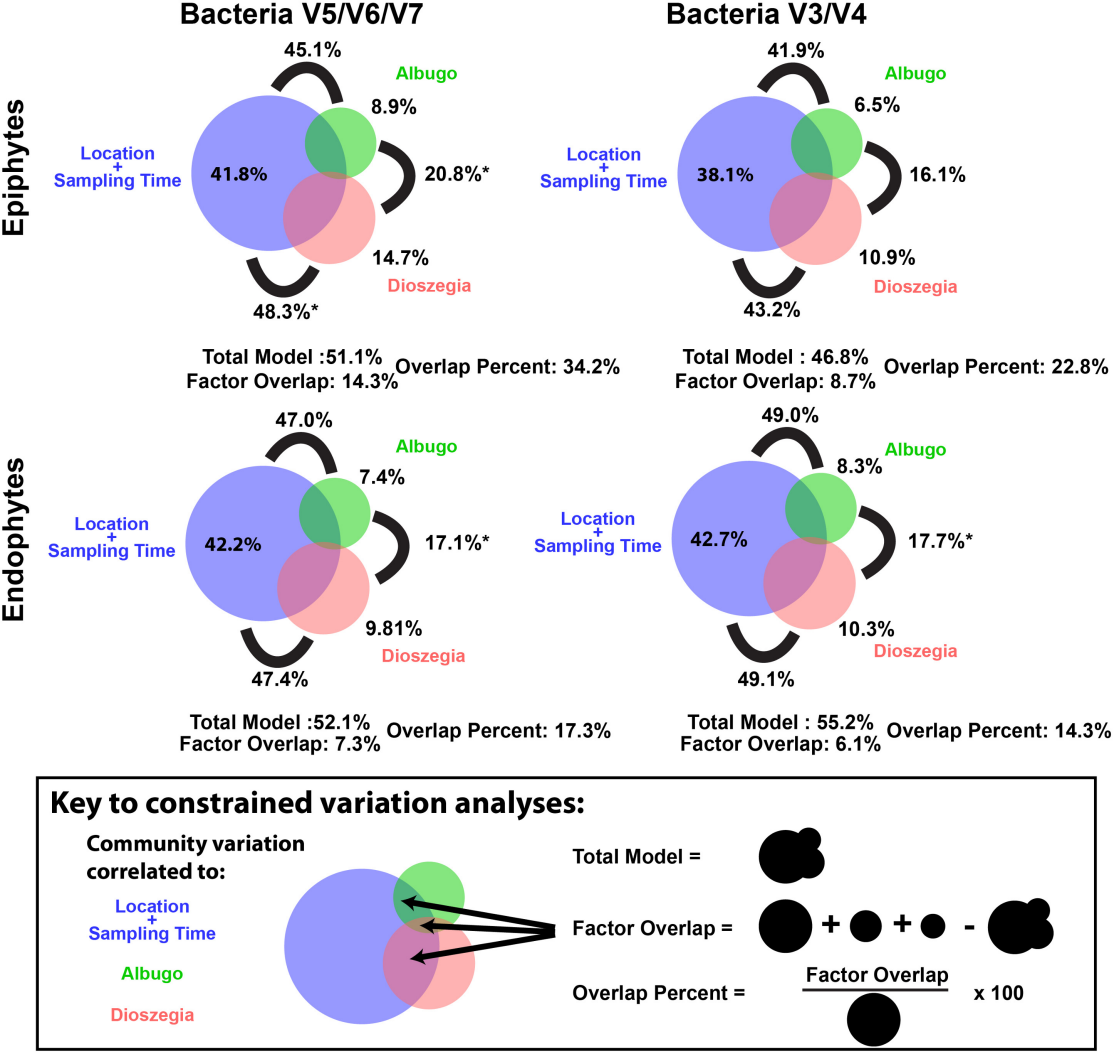
Experiment 1 and 3: Effects on bacterial colonization of eukaryotic “hub” microbes overlap with effects of location and sampling time. Each bubble represents the amount of microbial community variation between samples at Tübingen wild sites (Experiment 1) that could be correlated to the factor’s location and sampling time (blue), *Albugo* abundance (green), and *Dioszegia* abundance (red) using constrained ordination analysis. About 40% of observed variation in both epiphytic or endophytic bacterial colonization could be attributed to the external factors location and sampling time and about 50% when considering *Albugo* and *Dioszegia* in addition (total model). For epiphytes and endophytes, respectively, about 20%–35% and 15%–20% of variation (the overlap percent) linked to location and sampling time could also be correlated to either *Albugo* sp. or *Dioszegia* sp. (The “overlap percent” is the “factor overlap” divided by location/sampling time-correlated variation, where “factor overlap” is the percent of total community variation shared by *Albugo*/*Dioszegia* and location/sampling time). Black lines show the percent variation correlated to pairs of factors, and stars indicate that the two factors connected by the black line were significantly (*p* < 0.05) independent of one another.

Besides affecting relative abundances of specific bacterial groups, hub microbe abundance is itself affected by abiotic or host factors like climate, distribution, or host resistance alleles. Therefore, we used constrained ordination to ask to what extent external factors and microbial hubs are responsible for observed beta diversity variation. The external factors location and sampling time together correlated to ~40% of total epiphytic or endophytic bacterial variation. The hub microbes *Albugo* sp. and *Dioszegia* sp. together correlated to about 15%–20% of variation (Fig 5). External factor and hub microbe effects were not completely independent, since up to 34% of variation correlated to external factors overlapped with variation correlated to hub microbes (~14.3% of 41.8% for bacterial epiphytes based on V5/V6/V7 amplicons, Fig 5). Therefore, the external factors location and sampling time have important independent effects on phyllosphere microbiome structures, but up to one-third of their observed effects could be due to variation of two hub microbes.

## Discussion

Evidence has mounted that the holobiont is the unit on which evolutionary selection acts, but a full understanding of this concept, especially in plants, is missing complete explanations of how the metaorganism forms and is structured [1,2]. To elucidate principles enabling identification of mechanisms relevant for formation of the microbial fraction of the plant holobiont, we have generated an unprecedented high-resolution microbiome “map” showing a significant impact of biotic and abiotic factors. Analysis of three of the most important phyllosphere taxa (oomycetes, fungi and bacteria) addresses a lack of data with a broad taxonomic resolution which has prevented identification of specific mechanisms of microbial community differentiation. Our results suggest that mechanisms contributing to observed abundances are taxa-specific and are mediated by complex interactions between abiotic factors and taxa, between taxa and the host, and between multiple taxa. Sampling location (correlating to ~25%–35% of community variation) and sampling time (season, correlating to ~10% of community variation) were correlated to robust patterns of diversity variation caused by taxa that were not evenly distributed among sampling sites or times or which were completely specific to certain sites. Amplicon sequencing results in taxonomic resolution below the strain level, so microorganisms might be even less evenly distributed than our data suggests. This is illustrated by the oomycete genus *Albugo*, which was dominant and widely dispersed but which had site-specific strains that might be adapted to local conditions or hosts. Consistently, previous work on both phyllosphere and rhizosphere bacterial communities showed that location is a strong determinant of microbial community structures, which then vary to a lesser extent between different host species and genera [19,46]. Site-localized microbial taxa could result from both poor dispersal between sites (e.g., uneven distribution of microbial inocula like different soil conditions which differentially inoculate plants [12,14]) or local sorting mechanisms that completely exclude species in specific locations (e.g., local conditions or host plant effects [47,48]) [17]. Indeed, colonization of the phyllosphere and rhizosphere has been suggested to proceed with an ordered effect of inocula distribution followed by species sorting [12,49].

Distribution, dispersal, and species sorting to some extent go hand in hand, since strong adaptation of microbes to specific hosts have reproductive costs [50] and in some cases can limit their transmissibility [51]. In our study, it was clear that species sorting occurred at least in part due to hosts. For example, abundance of the genus *Albugo* was reduced (in the CG experiment) or eliminated (in lab experiments) due to (partial) resistance in the accession *A*. *thaliana* Ksk-1. We also observed much lower endophytic bacterial and fungal diversity than epiphytic caused by the “gateway” between the leaf surface (epiphytes) and interior (endophytes). This level of sorting occurs since endophytes and pathogens need to specialize and coevolve with hosts [52,53] to avoid or evade an arsenal of host self-defense mechanisms such as callose deposition [54], antimicrobial peptides [55], and reactive oxygen species (ROS) bursts [56]. Recent studies seeking to more generally connect host adaptation to microbiota colonization have utilized mutant plants [57,58] and genome-wide association studies (GWAS) [59] to demonstrate that plant genotypes sort their associated microbiota. Most direct allele or host accession effects, however, have only been minor and on specific taxa [21]. Comparatively, we observed significant effects on many diverse taxa in the CG experiment in this study, raising the question of what leads to broader host genotype impacts on microbial colonization. We proposed that one mechanism can be via microbe–microbe interactions. For example, despite high *A*. *thaliana* diversity between sites, we observed the genus *Alternaria* inside plant samples at almost all sites. We did not expect this because it is a plant pathogen with a necrotrophic lifestyle and host specificity on certain *A*. *thaliana* accessions [60]. This could be indicative of diverse strains with different host adaptations (i.e., broad compatibility as a genus), but an effectively expanded host range could also result from taking advantage of already broken down host barriers. For example, wide cooccurrence of *Alternaria* has been observed with *Alb*. *candida* [61].

**Fig 6:**
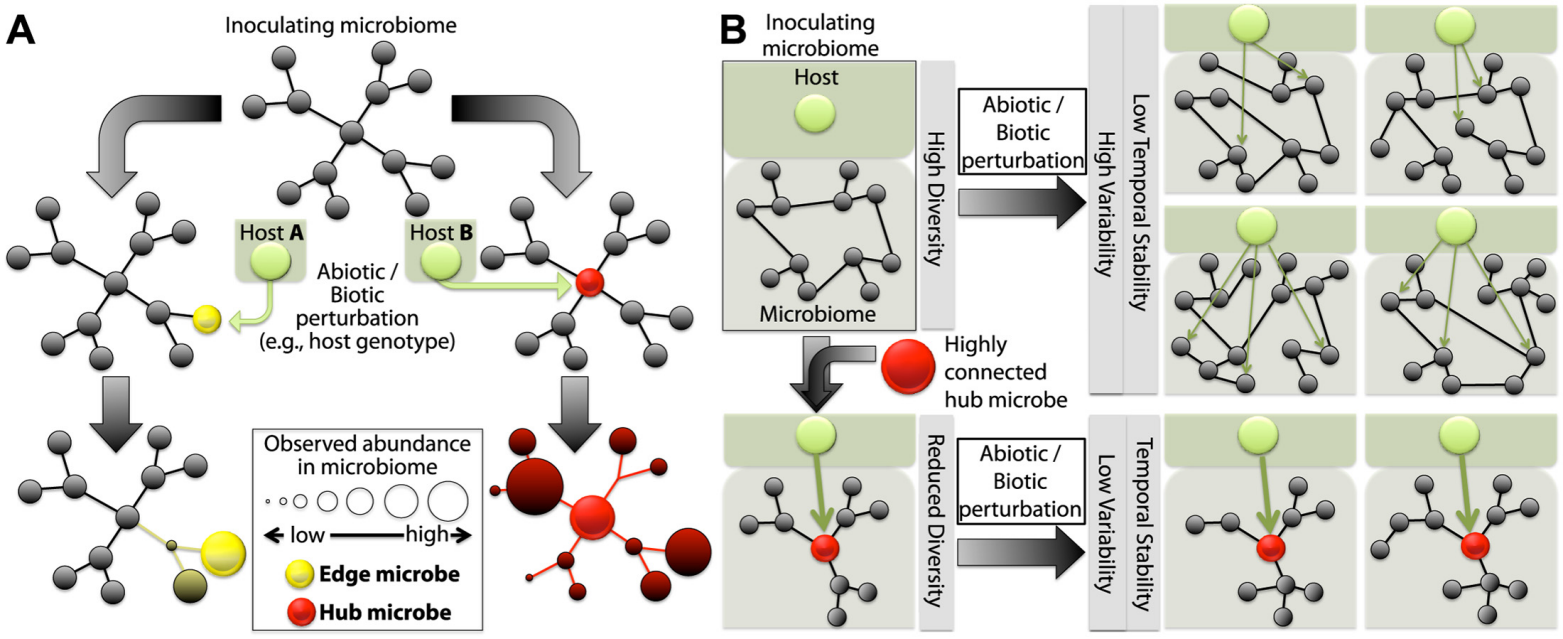
Direct targeting of a hub by a biotic or abiotic factor results in a cascade of abundance shifts throughout the community. A. The magnitude of effects of host or abiotic factors on microbial community structures is dependent on the connectivity of microbes targeted by the factor. For example, host “A” directly limits the colonization of an “edge” microbe (with low degree in the community network), and a relatively small shift in beta diversity is observed compared to the inoculating microbiome. On the other hand, when host “B” affects the colonization of a “hub” microbe, a drastic shift of many members of the inoculating microbiome is observed. B. A microbial community without a main “hub” microbe shows high fluctuations in beta diversity upon perturbation by different factors that might act on different “edge” microbes differently and is likely to be more subject to stochastic variation. A microbial community that is structured by a main “hub” microbe shows lower levels of fluctuation in beta diversity but is highly sensitive to perturbations of the “hub” microbe.

We propose that microbe–microbe interactions generally increase host effects due to the community correlation network topology we observed in which many microbes are weakly connected, while only a few “hubs” are highly connected, dominant interactors. In other words, many genotype effects (or other factors) will only perturb the activities of less influential microbes. If, on the other hand, an external pressure “hits” a hub microbe, the disturbance can be expected to cascade through the microbial community (Fig 6A). In this study, *Albugo*, the causal agent of white rust, was identified as an important hub. To show experimentally its hub status, we performed microbial “knockout” experiments in a CG experiment and in the lab, by introducing a range of different *A*. *thaliana* accessions carrying functional resistance alleles [45,62,63] or by physical/chemical removal/inhibition of *Albugo* in the lab (simulating an abiotic elimination of *Albugo* infection). We could show that, regardless of how *Albugo* is removed from the system, the associated microbial community is more stable in the presence of the pathogen and significantly changes in its absence. *Albugo* functions as a hub from the “bottom up” by limiting bacterial diversity and increasing relative abundance of major taxa in the phyllosphere. This supports the hypothesis that it also stabilizes abundance of site-specific taxa in the wild since hubs will promote deterministic host-associated taxa selection at affected sites (either directly or by modifying host phenotypes) (Fig 6B). On the other hand, community stability in the absence of major hubs is functionally based and occurs from the top down such that many observed taxa vary stochastically (Fig 6B). Here, abundant taxa are the target of perturbations that eliminate them or reduce their abundance and rare community members are required to fill the resulting open niches or functional voids [64]. Therefore, *Albugo* absence can plausibly explain strongly differentiated bacterial communities at the wild site PFN and significant accession-correlated microbial community differentiation in our garden experiment.

With the apparent importance of specific microbes for local species sorting in the phyllosphere, the question arises as to what makes these microbes hubs? One possibility is that they can exert strong indirect effects on other microbes via the host. On plants where host genetic diversity is a result of selection under pathogen pressure [65], pathogens can cause phenotypic expression of that diversity. At least some of the observed effects by *Albugo* sp. probably occur in this way, since it has been shown to, for example, alter host metabolism [30], which could lead to community differentiation. Therefore, from the point of view of its transformative effects on the host, the hub status of *Albugo* is not too surprising. Other more specific antibiosis selection mechanisms such as direct interactions and inhibition likely occur through, for example, ecological effectors [52,66]. No such pathways have been identified in *Albugo* genomes [67], but single protein effectors cannot be excluded. Our results suggest that the hub microbe *Dioszegia* directly inhibits some taxa, since it affected colonization efficiency of only specific bacteria. Not only *Dioszegia* but also other basidiomycetous yeasts [68] can directly interact with microbiota, and these are also likely to operate as microbial hubs. Plant-associated species like *Rhodotorula* and *Pseudozyma*, for example, are known for compounds secreted that are effective in “biocontrol” of bacteria and fungi, respectively [69,70]. Such direct effects would then be expected to cascade through the interconnected community. Thus, hub microbes can influence diversity by acting indirectly via the host or directly on colonization efficiency of other microbes.

Both indirect (via host [71]) and direct effects (via metabolites [72]) have been suggested for mechanisms of action of some microbes that cause abnormal human microbiomes. The hub status of these pathogens (and the dysbiotic microbiota they cause) is suggested to benefit them by promoting disease in the host [73]. Due to their disproportionally high impact on the metacommunity, these hubs are called “keystone” species [74]. Therefore, being a hub with a high level of “keystoneness” [44], as we have detected specifically for *Alb*. *laibachii* may be a critical part of host colonization. This might explain why *Alb*. *candida* was absent on wild *A*. *thaliana* (even where compatible strains were found on nearby *Capsella* sp. plants) since it was only weakly able to control *A*. *thaliana* microbial communities in lab experiments. Therefore, while hub interactions can occur indirectly through the host, where benefits of being a hub microbe can be identified, researchers should consider that strong selection exists for the ability of hubs to directly select cocolonizing microbiota.

Not all pathogens, however, share the hub microbe or even keystone status, so pathogenicity cannot be taken as a rule to detect “hub” or “keystone” species. The second most abundant oomycete genus that we recorded on *A*. *thaliana* was another obligate biotrophic pathogen, Hpa. Although Hpa was common, it was not a hub at the broad geographic and host diversity scales that we tested since it was not a strong interactor in the network and did not significantly correlate to bacterial diversity (S7 Fig, adjusted r^2^ value of 0.27 for correlation to epiphytic bacterial diversity). We still cannot exclude that Hpa might act as a hub within a specific *A*. *thaliana* population or by interacting with an *A*. *thaliana* genotype not in our survey. However, we hypothesize that the lack of hub status across broad scales compared to *Alb*. *laibachii* reflects fundamental differences in the biotrophic strategies of the pathogens. For example, a disproportionate number of hybrid incompatibility (HI) loci in *A*. *thaliana* encode leucine-rich repeat containing (NLR) resistance proteins to Hpa [65,75]. This evidence of active, strong selection at HI loci suggests that Hpa must have significant consequences for host fitness. While alleles are known that encode NLR proteins conferring resistance to *Alb*. *laibachii* [63,76], there are comparatively few, and none have been implicated as HI loci. Thus, we hypothesize that the “hub” characteristic of *Alb*. *laibachii* that leads to a low diversity and a stable phyllosphere microbiome is part of an “under-the-radar” approach to biotrophy. We therefore hypothesize that pathogens like Hpa, which thrive by participating in an extremely active evolutionary “arms race,” should exhibit less microbiome control.

*Dioszegia* and *Albugo* were functionally redundant with regard to decreasing bacterial diversity. Functionally redundant hubs in networks are characteristically stable, because the loss of one hub minimally interrupts function [77] and so this may suggest a relationship between these organisms. Interestingly, even after many generations of almost continuous subculturing in the lab (> 8 yr [67]) of *Alb*. *laibachii* Nc14, the basidiomycetous yeast *Pseudozyma* sp. is by far the most abundant associated fungus (S23 Fig). Associations of basidiomycetous yeasts including *Dioszegia* with other eukaryotes on plants such as arbuscular mycorrhiza fungi (AMF) and their spores [78] is known. Therefore, a close association and even beneficial relationship could exist between yeast and *Albugo* by limiting growth of complementary sets of microbes. Other relationships may also exist: other hub microbes (e.g., *Caulobacter* and a *Comamonadaceae* genus) seemed to have opposite effects on phyllosphere bacterial diversity. In at least one study, host manipulation of bacterial diversity has been suggested to affect its resistance to pathogens [79]. Thus, diversity manipulation might be a key battleground where hosts and various hubs cooperate or compete with one another. In this case, hubs with complementary or opposite microbial community functions are attractive targets for biocontrol studies in plants. Since pathogens have been identified as influential hubs in human hosts as well [71,72], a similar approach can be used there to find new targets for disease therapies.

Taken together, our results demonstrate that phyllosphere colonization by bacteria, fungi, and oomycetes is determined by various mechanisms of species sorting. These include seasonal effects, partitioning between epiphytic and endophytic leaf compartments, and host genetic differences. Most effects so far attributed to these factors have implicitly assumed their direct effect on microbes. Our broader-resolution study, however, strongly suggests that “hub” microbes are important intermediaries between abiotic, temporal, and host factors and colonization of many other microbes in the phyllosphere. Although previous studies have postulated the existence of keystone microbes in the phyllosphere [80,81] or suggested their existence based on bacterial network analyses [82,83], this is the first study to identify and confirm hubs from various kingdoms, to show their effects across kingdoms, and to identify hub microbes as direct targets of abiotic or host factors and mediators of observed microbiome variation. Because of complementary or antagonistic functions of these hubs, their resolution in plant, human, and other host contexts will improve understanding of what a holobiont is and how it functions. Specifically, if indeed hubs select cocolonizing microbiota to improve their own fitness, the host holobiont has to be understood in the context-colonizing hubs which themselves are holobionts. For example, host–pathogen coevolution can be expected to occur both on the molecular and microbial level. Thus, identifying hub interactions will reveal central targets to quickly revolutionize how we understand host–microbe–microbe relationships and to enable better future management of plant microbiomes—a crucial tool for biocontrol and resource saving food security.

## Materials and Methods

### Sampling Wild Populations of *A*. *thaliana* (Experiment 1)

We selected five sites near Tübingen in southern Germany for collection from wild populations of *A*. *thaliana* (S1 Table). These sites were selected because plants grew in open conditions in discrete populations with minimal disturbance from other plants such as grasses. Genotypic diversity within these populations was previously studied [39]. At two time points, in the spring and fall (5/7/13 and 11/26/13), we harvested several samples from each site. Because white rust caused by *Albugo* sp. was an extremely common phenotype at most sites, we recorded whether or not it was observed on collected plants. Where we recorded visible white rust (S2 Table), all leaves in the pool had visible white rust. Commonly, plant leaves were extremely small, in which case we pooled leaves from multiple plants, and otherwise we pooled multiple collected leaves from single plants (S2 Table). When otherwise healthy plants had leaves that were extremely dirty or where >50% of the leaf area exhibited lesions (most likely through mechanical wounding, insects or other factors), these were avoided.

### CG Experiment (Experiment 2)

In the garden experiment, three *A*. *thaliana* accessions (Ws-0, Col-0, and Ksk-1) were planted in nine plots. Each plot consisted of 30 plants, 10 of each accession, that were ordered randomly in 5 rows and 6 columns. The plants had been germinated from sterile seeds sown on Jiffy seed pellets (Jiffy Products International BV), initially watered with 2 mL / 1 L of WuxAl Liquid Foliar nutrient (AgNova Technologies Pty Ltd). After 10 d in a long-day greenhouse (12-h light / 12-h dark), when the plants had the second set of true leaves, the peat pellets and plants were transferred to the field site on 10/18/12. On 5/5/13 and again on 5/10/13, three leaf samples (pooled leaves from single plants) from rosettes of different *A*. *thaliana* Col-0, Ws-0 and Ksk-1 plants were harvested (see compartmentalization protocol below). We harvested from plants from various locations in the nine plots, selecting plants that were setting seeds but on which visible symptoms of senescence were not observed.

### Infection of *A*. *thaliana* with Lab Strains of *Albugo* spp. (Experiment 4)

We conducted experiments with the lab strains of *Alb*. *candida* Nc2 and *Alb*. *laibachii* Nc14 that had previously been kept growing on *A*. *thaliana* Ws-0 or Col-TH0, respectively, > 1 yr. In short, leaf washes from infected *A*. *thaliana* Ws-0 plants were collected and they or controls in which spores were either filtered from the solutions or chemically inhibited were sprayed on the *A*. *thaliana* accessions Ws-0, Col-0, and Ksk-1 (2 pathogens x 2 inocula x 3 hosts = 12 treatments). In each experimental replicate, all treatments were kept together in the same growth chamber under identical conditions. After 12 d, leaf washes were collected from all 12 treatments and were used to reinoculate a second round of plants. After another 12 d, the epiphytic (leaf surface) and endophytic (intra/intercellular) microbial communities were recovered from collected leaves from each of the 12 treatments (S12 Fig). The infection experiments were performed in three replicates. Further details can be found in the supporting materials and methods (S1 Text).

### One-on-One Interaction Assays in Cocultures of *Dioszegia* spp. and Bacterial Isolates Grown on *A*. *thaliana* (Experiment 5)

Interaction between *Dioszegia* sp. and individual bacterial isolates was observed on *A*. *thaliana* Ws-0 seedlings grown under sterile conditions on 1/2 MS media. Bacteria and *Dioszegia* (see S8 Table for strain information) were grown in liquid 10% TSB and PD media [84] until they reached an OD_600_ of 0.6. The microbes were pelleted, suspended in 10 mM MgCl_2_, and 200 μl microliter were sprayed on the individual plants using an airbrush pistol (Conrad Electronics, Germany). Three-week-old seedlings were sprayed with *Dioszegia*, and 2 d later the bacterial isolate was sprayed. After one week, leaf discs (0.07 cm^2^) were punched out from single leaves, crushed with a mortar and pestle and suspended in 50 μl of water. The CFU's for bacteria and Dioszegia were determined by growing on 10% TSB plates containing Nystantin and PDA plates containing antibiotics, respectively.

### Compartmentalization of Epiphytes and Endophytes from Leaf Samples

From each leaf sample (wild collection, garden experiments, and lab experiments), leaf epiphytic and endophytic microorganisms were collected using the same protocol. In short, the collected leaves in a 15 mL tube were first rinsed with water by gentle agitation for 30 sec, from which an aliquot was taken and stored. Next, 3–5 mL of epiphyte wash (0.1% Triton X-100 in 1x TE buffer) was added to the tube, agitated for about 1 min, and filtered through a 25 mm, 0.2 μm nitrocellulose membrane filter (Whatman, Inc). The filter containing epiphytic microorganisms was placed in a screw-cap tube and frozen in dry ice. Next, the same leaves were surface sterilized first using 15 sec washes of 80% ethanol followed by 2% bleach (sodium hypochlorite). Leaves were then rinsed three times with sterile autoclaved water and the resulting leaves containing endophytic microorganisms were frozen on dry ice for further processing.

### DNA Extraction and Amplicon Library Preparation and Sequencing

We extracted DNA with a custom protocol and prepared amplicon libraries for ten samples from each of two wild collection events (always two samples from WH, two from ERG, three from EY, two from JUG, and one from PFN) and three samples of each *A*. *thaliana* accession from the garden experiment collected on 5/10/2013. From the controlled lab experiments, we prepared libraries for triplicates of each of 12 treatments. In total, bacteria, fungi, and oomycete amplicon libraries were prepared from 65 epiphyte and 65 endophyte leaf fraction samples (see S1 File for samples and index sequences). A two-step amplification protocol was implemented, and the first step was prepared in triplicate. Primers consisted of a concatenation of the Illumina adapter P5 (forward) or P7 (reverse), an index sequence (reverse only), a linker region, and the base primer for the region being amplified. For each region, we used 20 different reverse primers that were identical except for the 12-bp index [85] that would be used later to identify sequencing products in combined libraries. Information for all primers used can be found in S2 File.

Amplicon libraries were quantified fluorescently, and products of 120 amplicon libraries (the six targeted amplicon regions from epiphyte and endophyte templates from ten samples) were combined in equimolar concentrations in seven combined libraries. The combined libraries were concentrated and quantified via qPCR and were sequenced on an Illumina MiSeq lane using a mixture of custom sequencing primers complementary to the linker/primer region of the concatenated primers (S2 File). Sequencing was performed for 500 cycles to recover 250 bp of information in the forward and reverse directions. Additional details can be found in supporting materials and methods (S1 Text).

Raw sequence data is publicly available online through MG-RAST project number 13322 [http://metagenomics.anl.gov/linkin.cgi?project=13322].

### Processing Amplicon Data

We developed a custom pipeline to simultaneously process reads from bacteria, fungi, and oomycetes for downstream analysis. In short, for data from each Illumina lane, we de-multiplexed and quality filtered reads, split sequence files into the six amplicon groups, and separated reads that were still paired or were orphans after filtering. We then trimmed adapter sequences and aligned paired reads. Next, we placed all aligned paired, unaligned, and orphan reads together and checked for chimeras then combined reads from which the first 125 or last 125 bases were identical (since all orphan reads were at least this long). We then combined the prefix/suffix combined reads from all sequencing runs and picked OTUs at 97% similarity and picked representative sequences for each OTU. Finally, OTUs were assigned taxonomy, and filters were applied to remove low abundance OTUs and nontarget amplicons. For downstream analyses, OTU tables were rarefied to an even depth of reads per sample and summarized to a specific taxonomic level (usually genus except where noted). More details on softwares used and processing parameters can be located in supporting materials and methods (S1 Text). Data and code used to generate the main figures in the text are being made available on GitHub (https://github.com/magler1/HubMicrobes).

For further details on downstream statistical analyses and other details not included in the main text, please refer to the Supporting Materials and Methods (S1 Text).

## Supporting Information

S1 Data Data supporting all data-based figures in the manuscript. (XLSX)

S1 Fig Experiment 1 and 2: Ecological and host factors are important in shaping phyllosphere microbial communities—additional datasets complementary to Fig 1. A. Experiment 1: Sampling location and sampling time correlated to significant portions of microbial community structure variation observed between Tübingen wild sites. Circles and triangles are samples collected in fall and spring, respectively. Colors of points illustrate the location where the samples were collected. Dot plots are unconstrained endophytic communities, while barcharts show factor correlations to endophytic (endo) and epiphytic (epi) variation. Overlap of bars represents factors correlated to the same variation. B. Experiment 2: The host *A*. *thaliana* accession correlated to significant portions of microbial community structure variation observed in the CG experiment. Colors of points represent the host accession. For A and B, figures are based on genus-level data from bacterial 16S V5/V6/V7 region, fungal ITS2 region and oomycete ITS2 region amplicons. For A and B, a star indicates that the measured correlation is statistically significant (*p* < 0.05) based on random permutations sample classes.
(TIF)

S2 Fig Experiment 1: Boxplots of alpha diversity (number of observed genera) for endophytic and epiphytic bacteria and fungi by location. Locations include CG (Experiment 2), and the wild Tübingen sites ERG, EY, JUG, PFN, and WH (Experiment 1). Letters indicate significant difference based on *t* test, *p* < 0.1.
(TIF)

S3 Fig Experiment 1 and 2: Abundant fungal endophytes are less widely distributed than fungal epiphytes or bacteria. Figures show the number of sites where individual genera (each dot represents one genus) are observed (site CG [Experiment 2] and wild Tübingen sites ERG, EY, JUG, PFN, and WH, Experiment 1). The red dashed line is provided at a total observation depth of 500 to make comparison easier. *Y*-axes were scaled to 2,500 observations for direct comparison between plots. Inset figures show expanded *y*-axes so that all genera are visible. To make all plots comparable, all data sets were subsampled to 1,000 reads per sample.
(TIF)

S4 Fig Experiment 1 and 2: Bacterial genera that make up at least 10% of reads in any one sample. Legends are common for the barcharts in the figure. Data is based on relative abundance calculated from data that was not first subsampled. Key: CG: Cologne garden experiment [C: Col0, K: Ksk1, W: Ws0], and the Tübingen wild sites: ERG, EY, JUG, PFN, WH [F: Fall, S: Spring].
(TIF)

S5 Fig Experiment 1 and 2: Fungal genera that make up at least 10% of reads in any one sample. Legends are common for the barcharts in the figure. Data is based on relative abundance calculated from data that was not first subsampled. Key: CG: Cologne garden experiment [C: Col0, K: Ksk1, W: Ws0], and the Tübingen wild sites: ERG, EY, JUG, PFN, WH [F: Fall, S: Spring].
(TIF)

S6 Fig Experiment 1 and 2: Oomycete genera that make up at least 10% of reads in any one sample. Legends are common for the barcharts in the figure. Data is based on relative abundance calculated from data that was not first subsampled. Key: CG: Cologne garden experiment [C: Col0, K: Ksk1, W: Ws0], and the Tübingen wild sites: ERG, EY, JUG, PFN, WH [F: Fall, S: Spring].
(TIF)

S7 Fig Experiment 1 and 2: Abundance of endophytic *Albugo* sp. is correlated to the measured endophytic and epiphytic bacterial diversity. Plotted points are scaled to measured endophytic Hpa (based on the log of the average of ITS1 and ITS2 datasets relative abundance information. Relative abundances were scaled from 0–1 before averaging so that information between the two datasets would be comparable). The color of the plotted points corresponds to whether or not “white rust” caused by *Albugo* sp. was observed on the sample.
(TIF)

S8 Fig Experiment 2 and 4: *Albugo* sp. was detectable only at very low background levels in control plants for *Albugo* sp. experiments, demonstrating effective host resistance and *Albugo* removal from inoculum. W: *A*. *thaliana* Ws-0, C: *A*. *thaliana* Col-0, K: *A*. *thaliana* Ksk-1. Yellow: *Albugo* sp. spores physically removed from inoculum by filtering, Green: Susceptible plants inoculated with *Albugo* sp. and associated microbes, Red: Resistant plants inoculated with *Albugo* sp. and associated microbes.
(TIF)

S9 Fig Experiment 2: Relative diversity of observed infecting strains of *Alb*. *laibachii*, and observed white rust symptoms were dependent on host ecotype in the Cologne garden experiment (CG). The *A*. *thaliana* accession Ksk-1 carries an allele for resistance to *Albugo* sp., which lent partial resistance to the wild pathogen strains. Letters indicate significance at *p* < 0.1 (Tukey’s HSD).
(TIF)

S10 Fig Computational Experiment 3: The generated correlation network is highly supported within subsamples of the datasets. A. The correlation network, which is identical to that presented in Fig 2 in the main text. B. Edge support in the correlation network determined by randomly subsampling 50% of the data for each correlation 100 times.
(TIF)

S11 Fig Computational Experiment 3: Discovery of robust “hub” microorganisms (shown for genus-level microorganisms) in microbial correlation networks. A. Comparison of the effects of different cutoffs for edge filtering (removing weak correlations between taxa) for three measures of node (taxa) centrality, where yellow highlights taxa that are significantly more central in the network using a specific cutoff. ratio of abundance sum to maximum abundance (STM) x R^2^ > 3 is a cutoff we designed that accounts for distribution of the microbes and the strength of the correlation (see Supplementary Materials and Methods [S1 Text] for details). Genera that were detected with any one of the filters were considered a possible “hub” microbe. B. Only taxa that were discovered using all three metrics were considered as likely “hub” taxa (see S7 Table for other taxonomic levels). Genus-level “hub” taxa are highlighted in A.
(TIF)

S12 Fig Experiment 4: The experimental setup for laboratory experiments with the strains *Alb*. *laibachii* Nc14 and *Alb*. *candida* Nc2. Experiments were complemented with two different types of controls for the experimental infected *A*. *thaliana*. The first control were *Albugo*-free containing microorganisms that were associated with each *Albugo* strain but no *Albugo* spores. This control represents an abiotic factor, such as a distribution limitation, that in nature would limit the growth of *Albugo*. We tested both filter removal of *Albugo* spores (see Fig 4 and S13, S15 and S16 Figs) and chemical *Albugo* inhibition (see S14 Fig). The second control was resistant accessions (Ksk1 for Nc14 and Col0/Ksk1 for Nc2) that were inoculated with *Albugo* and its associated microbial community. This control represents a host factor, resistance, that in nature would limit the growth of *Albugo*.
(PDF)

S13 Fig Experiment 4: Boxplots of alpha diversity (observed number of taxa) of epiphytic microbial communities at both the order and genus level taxonomies for the three replicate lab experiments. Results demonstrate that Albugo-infected plants had significantly lower bacterial diversity than controls (lines indicate *t* test, *p* < 0.05). Key: Ws-0: *A*. *thaliana* Ws-0, Col-0: *A*. *thaliana* Col-0, Ksk-1: *A*. *thaliana* Ksk-1. Green: Susceptible hosts, Red: Resistant hosts, Yellow: Filter removal of *Albugo* on all hosts.
(TIF)

S14 Fig Experiment 4: Alpha diversity (observed number of taxa) of epiphytic bacteria and fungi at the genus level for one replicate experiment using chemical inhibition or host resistance to Albugo. A strong suppressive effect on bacterial alpha diversity by *Albugo* sp. was confirmed regardless of the mechanism of removal of *Albugo* (see Fig 4 and S13 Fig for removal of *Albugo* spores by filtering). Solid black lines are placed to facilitate comparison of control samples to *Albugo*-infected samples. W: *A*. *thaliana* Ws-0, C: *A*. *thaliana* Col-0, K: *A*. *thaliana* Ksk-1. Green: Susceptible hosts, Red: Resistant hosts, Yellow: Chemical inhibition of *Albugo* on all hosts by metalaxyl and benalaxyl.
(TIF)

S15 Fig Experiment 4: Boxplots of within-replicate distances (i.e., replicability based on chi-square distances of log-transformed microbial abundance data) of epiphytic microbial communities at both the order and genus level taxonomies for the three replicate lab experiments. Results demonstrate that the final microbial communities recovered from *Albugo*-infected plants were more similar between experimental replicates than controls (lines indicate *t* test, *p* < 0.05). Key: Ws-0: *A*. *thaliana* Ws-0, Col-0: *A*. *thaliana* Col-0, Ksk-1: *A*. *thaliana* Ksk-1. Green: Susceptible hosts, Red: Resistant hosts, Yellow: Filter removal of *Albugo* on all hosts. (S1_Data.xlsx)
(TIF)

S16 Fig Experiment 4: Boxplots of between-treatment distances (based on chi-square distances of log-transformed microbial abundance data) of epiphytic microbial communities at both the order and genus level taxonomies. Results demonstrate that bacterial communities at the order level from *Albugo*-infected *A*. *thaliana* was more similar to other infected plant communities than to uninfected controls (lines indicate *t* test, *p* < 0.05). For simplicity, not every possible comparison is shown. Key: Ws-0: *A*. *thaliana* Ws-0, Col-0: *A*. *thaliana* Col-0, Ksk-1: *A*. *thaliana* Ksk-1. Green: Susceptible hosts, Red: Resistant hosts, Yellow: Filter removal of *Albugo* on all hosts.
(TIF)

S17 Fig Experiment 4: Abundance of epiphytic bacteria on *A*. *thaliana* infected with *Albugo* based on fluorescent cell count data. Results demonstrate high counts of bacteria on *A*. *thaliana* leaves infected with *Alb*. *candida* Nc2 (*t* test, *p* < 0.05). Controls with inhibited spores used the oomycete-specific inhibitory compounds metalaxyl and benalaxyl.
(TIF)

S18 Fig Experiment 4: Numbers of recovered microbial endophytic reads (after filtering out plant plastid or plant ITS sequences) tended to be higher in infected compared to control leaf samples. For this analysis, bacterial reads identified as cyanobacteria at only the phylum level (with no more specific taxonomic assignment) have been removed. Indicated significant differences are based on a *t* test with *p* < 0.05.
(TIF)

S19 Fig Experiment 4: Additional endophytic microbe colonization data complementing Fig 4B. A. Genera with significant increase or decrease (*t* test, *p* < 0.05) on plants infected with *Alb*. *candida* compared to both controls. B. Genera without statistically significant (*t* test, *p* < 0.05) enrichment on two host accessions infected with *Alb*. *laibachii*. Some abundant taxa were not enriched due to *Alb*. *laibachii* infection, while others were only enriched on *Alb*. *thaliana* Ws-0 (the accession on which stock infections were kept) or were similar abundance in all treatments.
(TIF)

S20 Fig Experiment 5: One-on-One interactions between *Dioszegia* sp. and bacterial isolates in the phyllosphere measured with 1-wk coculture assays on *A*. *thaliana* Ws-0. A. *Dioszegia* growth with bacteria (letters indicate significant differences at Tukey’s HSD *p* < 0.05). *Dioszegia* growth was significantly negatively affected by *Rhodococcus* sp. and *Agromyces* sp. compared to growth alone. B. Bacterial growth alone or with Dioszegia (letters indicate significant differences with Tukey’s HSD *p* < 0.05 only within each bacterial isolate test, not between isolates). The growth of *Rhodococcus* sp. was slightly increased in the presence of *Dioszegia* compared to growth alone. The strongest effect was on *Caulobacter* sp. where strong growth was completely inhibited by *Dioszegia* sp. Details about bacterial isolates are provided in S8 Table. C. Observed interactions confirmed and gave direction to several correlations observed in our network analysis (green) and others (red) could be a result of indirect connections (black). *Rhodococcus* sp. interactions were not observed in the network analysis, but this was a lab isolate and the genus was very low abundance in the field.
(TIF)

S21 Fig Experiment 4: Bacterial genera that make up at least 10% of reads in any one sample in the lab experiment with *Albugo* sp. Legends are common for the barcharts in the figure. Data is based on relative abundance calculated from data that was not first subsampled. Key: Spores + microbes: inoculation of Albugo sp. and associated microorganisms, Microbes only: inoculation of associated microorganisms after filter removal of *Albugo* sp., W: *A*. *thaliana* Ws-0, C: *A*. *thaliana* Col-0, K: *A*. *thaliana* Ksk-1, Green: Susceptible host, Red: Resistant host, Yellow: filter removal of *Albugo* sp. on all hosts.
(TIF)

S22 Fig Computational Experiment 3: Correlations of bacteria, oomycete, and fungal genera to alpha diversity (number of observed taxa) for bacteria and fungi. Interkingdom correlations reveal several genera correlated significantly to fungal and bacterial epiphyte and endophyte diversity. Strong negative correlations of *Albugo* sp. and *Dioszegia* sp. reinforces their putative role based on paired microbe correlations in limiting abundance of many epiphytic bacterial genera.
(TIF)

S23 Fig Experiment 4: Fungal genera that make up at least 10% of reads in any one sample in the lab experiment with *Albugo* sp. Legends are common for the barcharts in the figure. Data is based on relative abundance calculated from data that was not first subsampled. Key: Spores + microbes: inoculation of Albugo sp. and associated microorganisms, Microbes only: inoculation of associated microorganisms after filter removal of *Albugo* sp., W: *A*. *thaliana* Ws-0, C: *A*. *thaliana* Col-0, K: *A*. *thaliana* Ksk-1, Green: Susceptible host, Red: Resistant host, Yellow: filter removal of *Albugo* sp. on all hosts.
(TIF)

S24 Fig Experiment 4: Flattening lines indicate near-complete sampling was achieved for bacterial endophytes in laboratory experiment samples. This effect was especially apparent in the V5/V6/V7 region where sampling was deepest. Data is based on observed number of bacterial genera and each line represents one sample.
(TIF)

S1 File Metadata for amplicon libraries matching library names and sequence barcodes to sample metadata. (XLSX)

S2 File Sequences of all amplification and sequencing primers as well as concatenated primers used for addition of Illumina adapter regions to amplicons. (XLSX)

S1 Table Locations of wild sampling sites (WH, ERG, JUG, EY, PFN) (Experiment 1) and CG (Experiment 2) and the types of sites. (DOCX)

S2 Table Experiment 1 and 2: Supporting data on all wild or common garden experiment samples collected that were used to generate data for this work. (DOCX)

S3 Table Experiment 1: Supporting data showing microbial genera that are enriched in abundance at specific sampling locations in Tübingen wild samples. Enrichment at a location is based a significantly higher relative abundance there compared to any other location (Tukey’s HSD *p* < 0.01).
(DOCX)

S4 Table Experiment 1: Supporting data showing microbial genera that are enriched in abundance at specific sampling times in Tübingen wild samples. Enrichment in fall or spring is based on a significantly higher relative abundance in that season compared to the other season (Tukey’s HSD *p* < 0.01).
(DOCX)

S5 Table Experiment 2: Supporting data showing microbial genera that are enriched in abundance on a specific host accession in the Cologne garden experiment. Enrichment on an accession is based a significantly higher relative abundance on that accession compared to any other accession (Tukey’s HSD *p* < 0.05).
(DOCX)

S6 Table Computational Experiment 3: Supporting information comparing numbers possible correlations (i.e., number of comparisons made) to number of correlations observed in the network analysis. (DOCX)

S7 Table Computational Experiment 3: Comparison of “hub” microorganisms discovered in correlation networks using similar constraints at various levels of taxonomic grouping of OTUs. (DOCX)

S8 Table Experiment 5: Details about isolation and taxonomic characterization of *Dioszegia* sp. and bacterial strains tested with it in direct interaction assays on *A*. *thaliana*. (DOCX)

S1 Text Supporting Materials and Methods. Text including details of materials and methods that were not included in the main text. (DOCX)

## Abbreviations

AMF: arbuscular mycorrhiza fungi
CFU: colony forming units
CG: cologne garden
GWAS: genome-wide association studies
HI: hybrid incompatibility
Hpa: *Hyaloperonospora*
HSD: honest significant difference
ITS: internal transcribed spacers
MHC: Major Histocompatibility class II
NLR: leucine-rich repeat containing proteins
OUT: operational taxonomic units
qPCR: quantitative polymerase chain reaction
ROS: reactive oxygen species
STM: ratio of abundance sum to maximum abundance
