# Supplementary figures and images for: Microbial Hub Taxa Link Host and Abiotic Factors to Plant Microbiome Variation
Source: PLoS Biol. 2016 Jan 20;14(1):e1002352. doi: 10.1371/journal.pbio.1002352 (PMC4720289; doi:10.1371/journal.pbio.1002352)

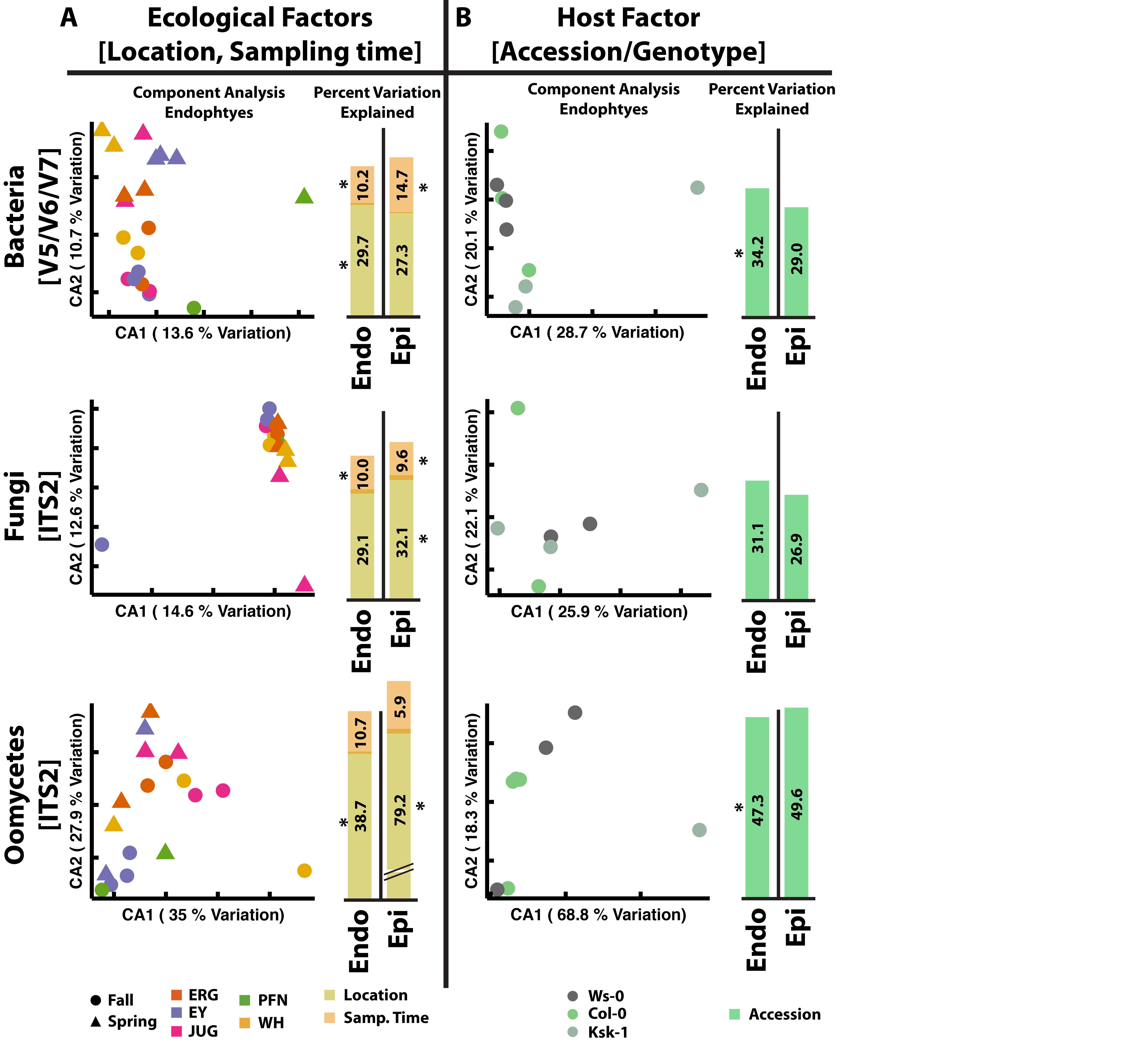

Supplement: S1 Fig — A. Experiment 1: Sampling location and sampling time correlated to significant portions of microbial community structure variation observed between Tübingen wild sites. Circles and triangles are samples collected in fall and spring, respectively. Colors of points illustrate the location where the samples were collected. Dot plots are unconstrained endophytic communities, while barcharts show factor correlations to endophytic (endo) and epiphytic (epi) variation. Overlap of bars represents factors correlated to the same variation. B. Experiment 2: The host A. thaliana accession correlated to significant portions of microbial community structure variation observed in the CG experiment. Colors of points represent the host accession. For A and B, figures are based on genus-level data from bacterial 16S V5/V6/V7 region, fungal ITS2 region and oomycete ITS2 region amplicons. For A and B, a star indicates that the measured correlation is statistically significant (p < 0.05) based on random permutations sample classes. (TIF) [file pbio.1002352.s002.tif]

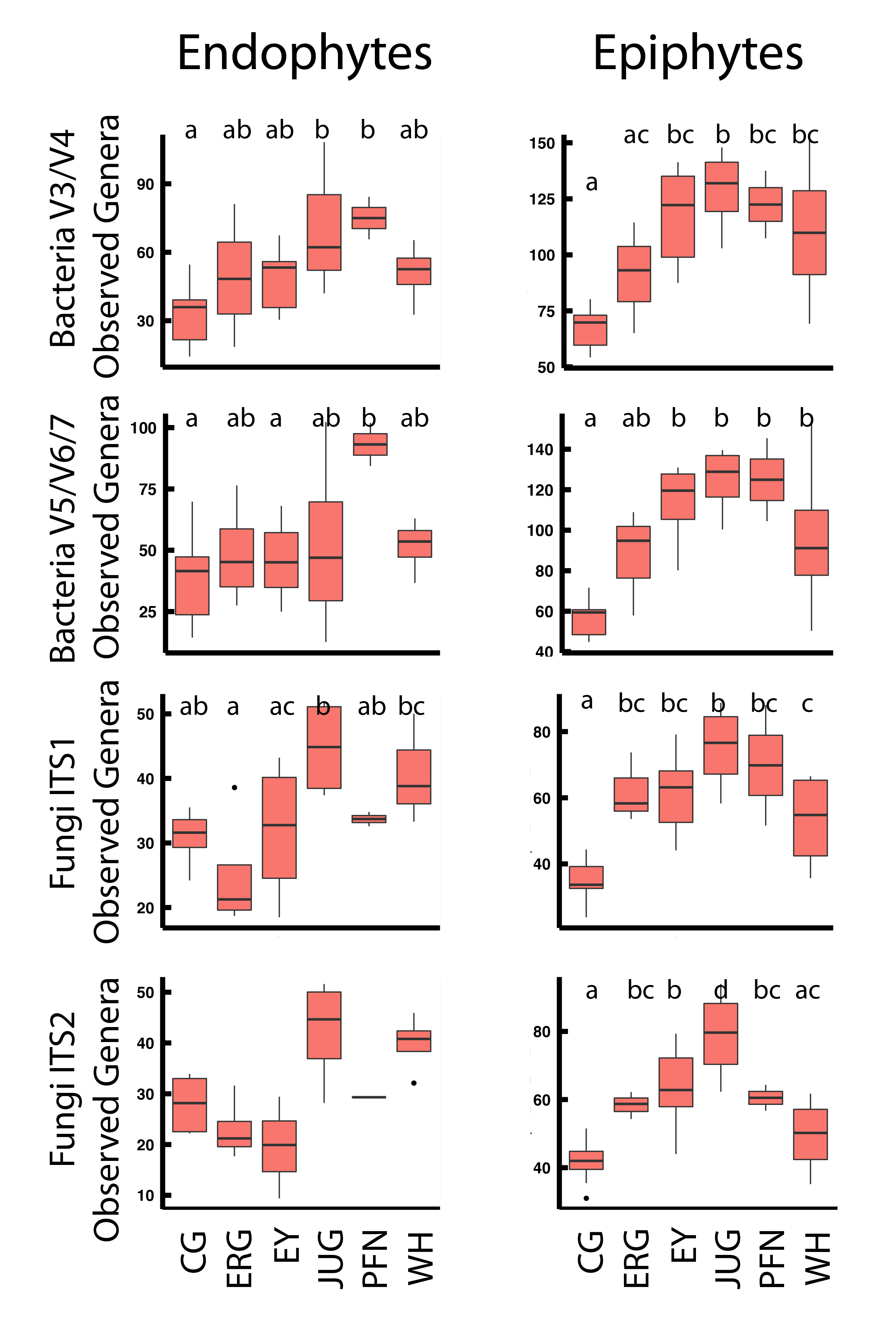

Supplement: S2 Fig — Locations include CG (Experiment 2), and the wild Tübingen sites ERG, EY, JUG, PFN, and WH (Experiment 1). Letters indicate significant difference based on t test, p < 0.1. (TIF) [file pbio.1002352.s003.tif]

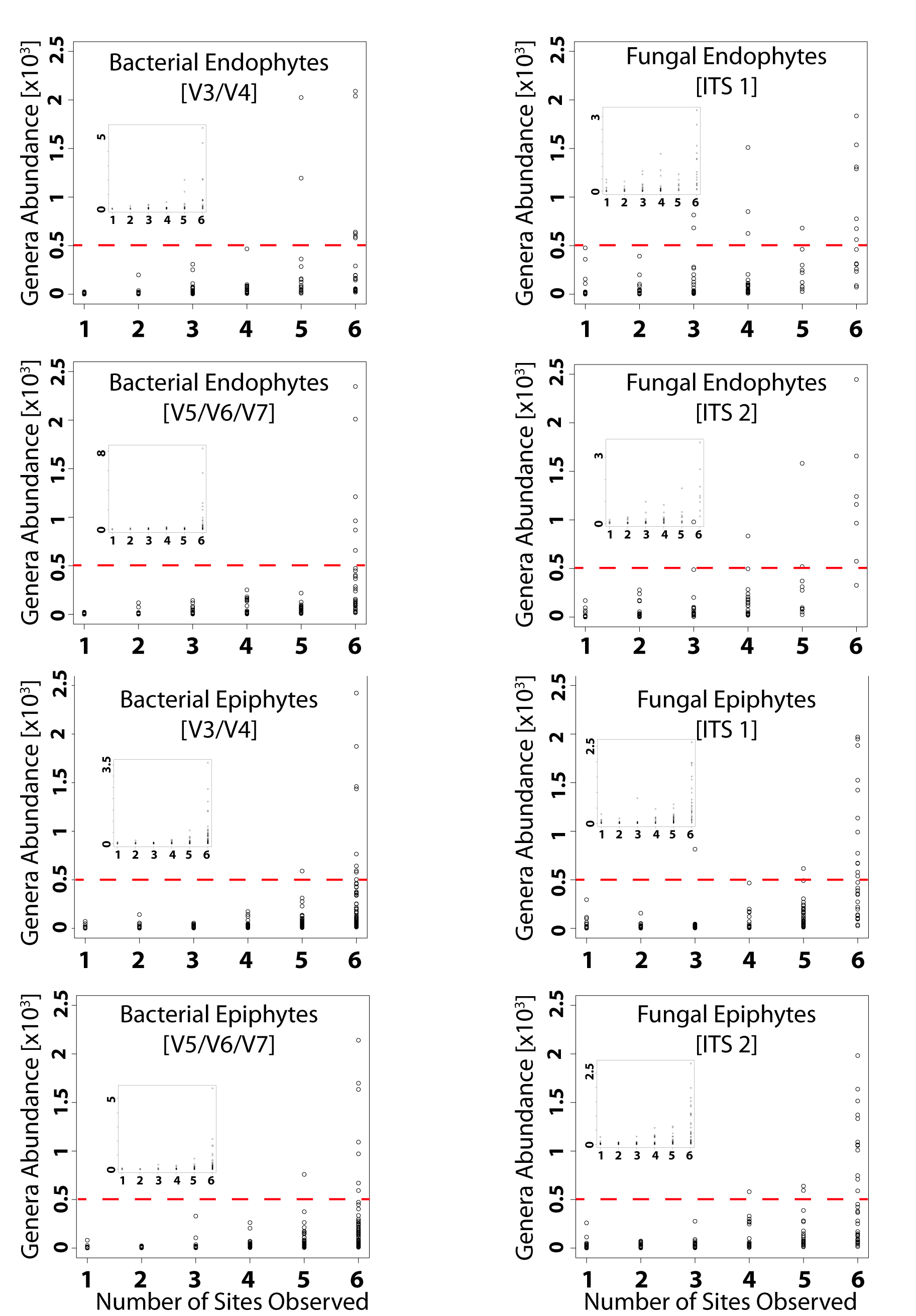

Supplement: S3 Fig — Figures show the number of sites where individual genera (each dot represents one genus) are observed (site CG [Experiment 2] and wild Tübingen sites ERG, EY, JUG, PFN, and WH, Experiment 1). The red dashed line is provided at a total observation depth of 500 to make comparison easier. Y-axes were scaled to 2,500 observations for direct comparison between plots. Inset figures show expanded y-axes so that all genera are visible. To make all plots comparable, all data sets were subsampled to 1,000 reads per sample. (TIF) [file pbio.1002352.s004.tif]

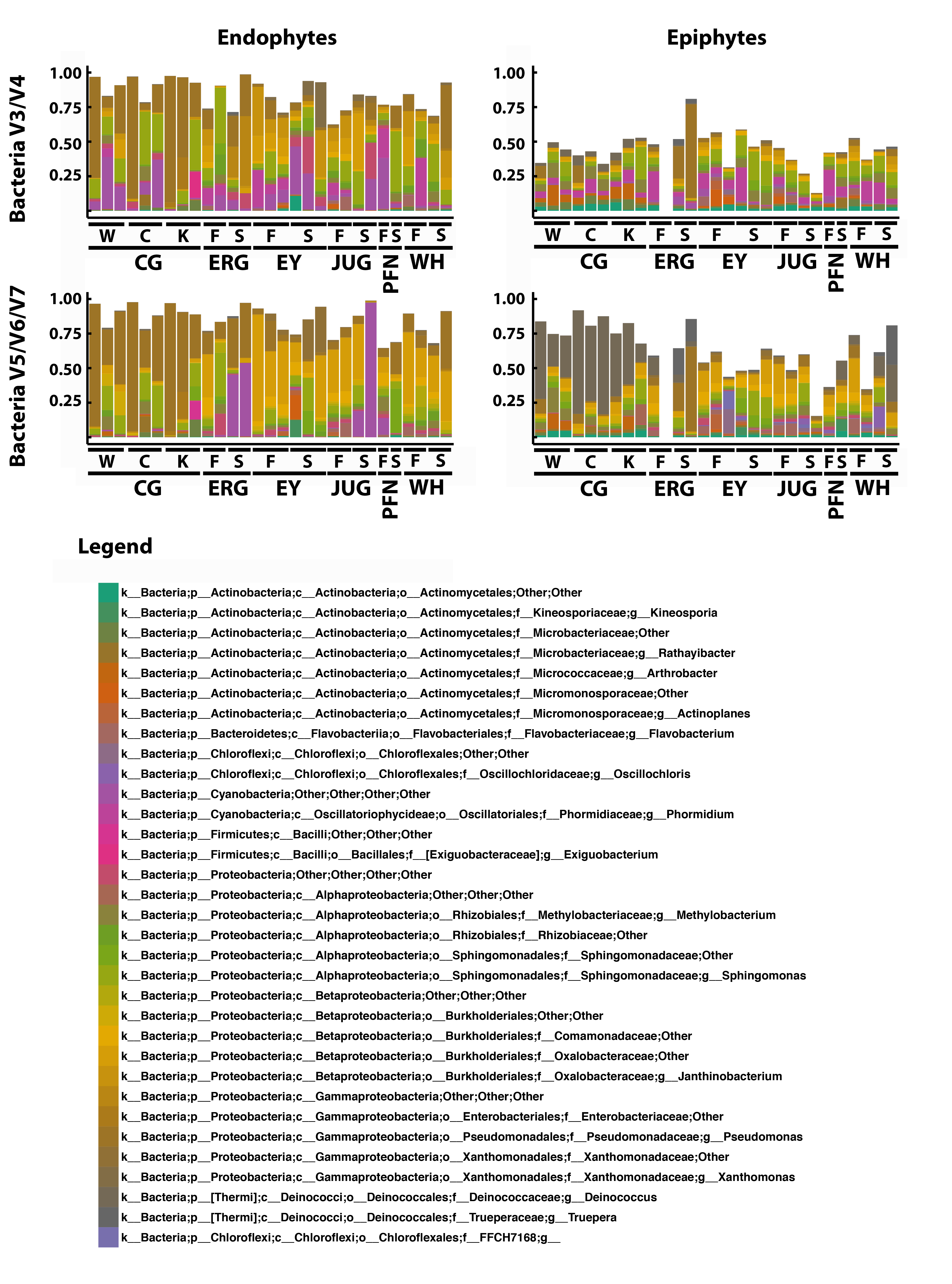

Supplement: S4 Fig — Legends are common for the barcharts in the figure. Data is based on relative abundance calculated from data that was not first subsampled. Key: CG: Cologne garden experiment [C: Col0, K: Ksk1, W: Ws0], and the Tübingen wild sites: ERG, EY, JUG, PFN, WH [F: Fall, S: Spring]. (TIF) [file pbio.1002352.s005.tif]

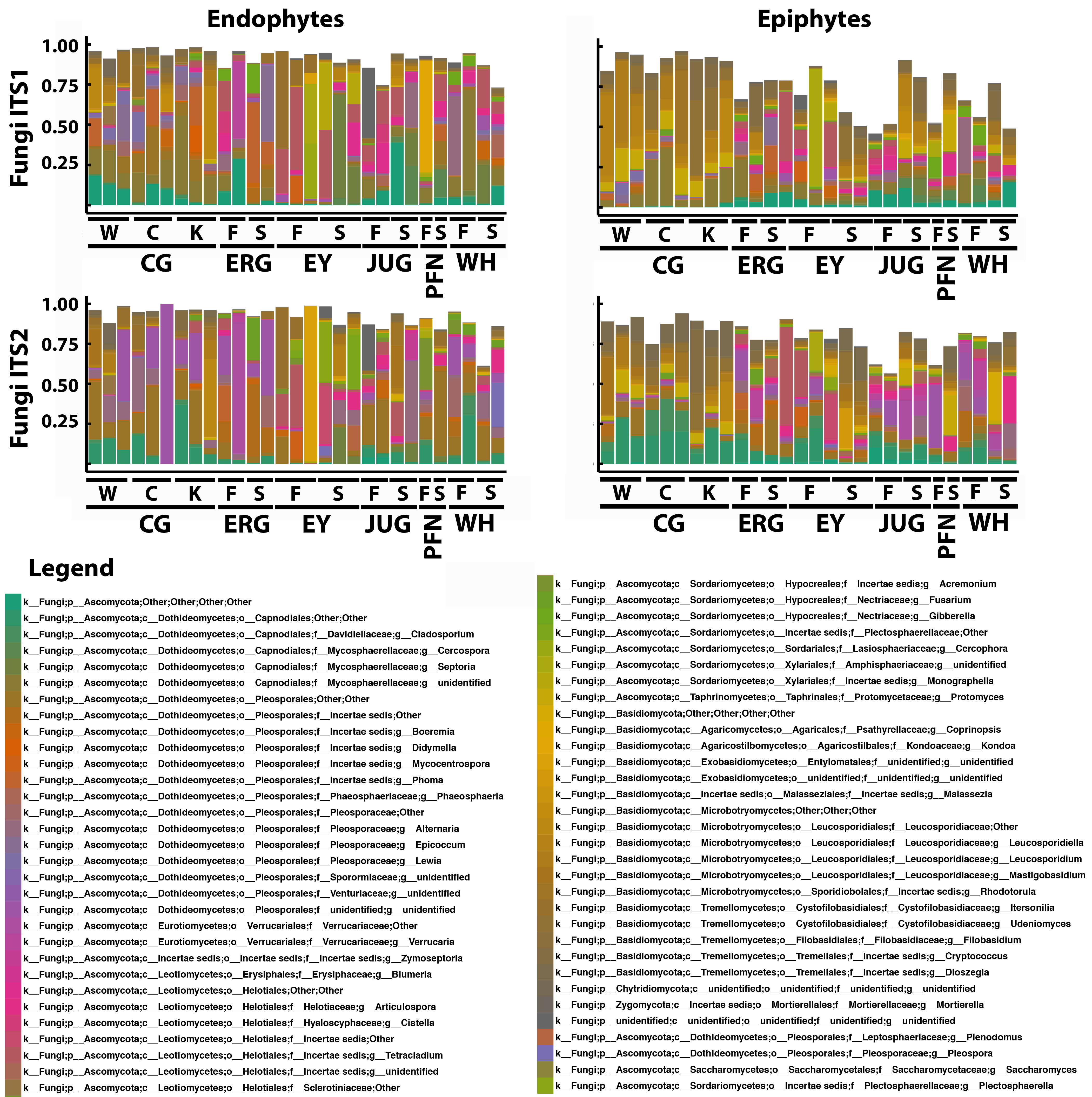

Supplement: S5 Fig — Legends are common for the barcharts in the figure. Data is based on relative abundance calculated from data that was not first subsampled. Key: CG: Cologne garden experiment [C: Col0, K: Ksk1, W: Ws0], and the Tübingen wild sites: ERG, EY, JUG, PFN, WH [F: Fall, S: Spring]. (TIF) [file pbio.1002352.s006.tif]

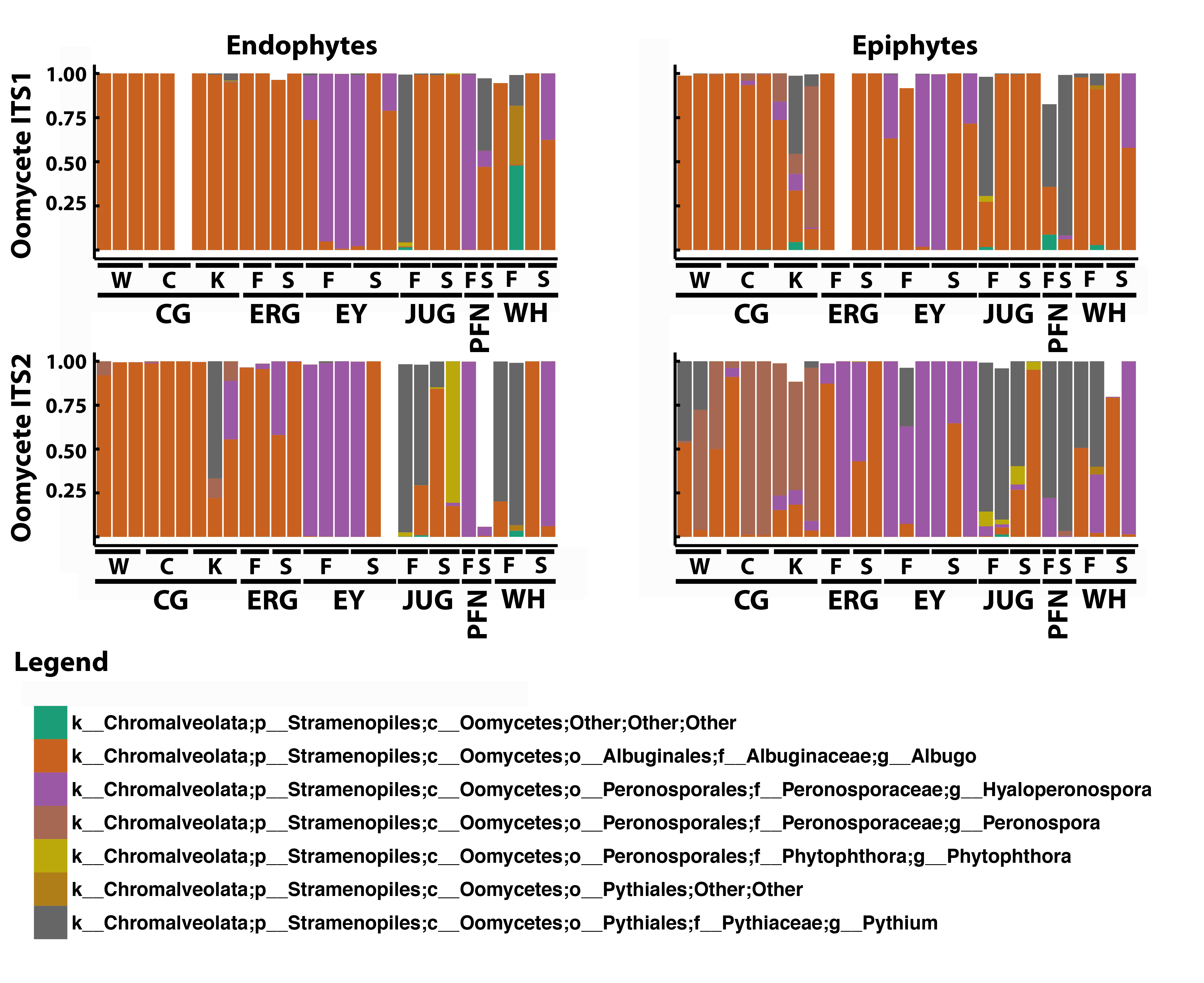

Supplement: S6 Fig — Legends are common for the barcharts in the figure. Data is based on relative abundance calculated from data that was not first subsampled. Key: CG: Cologne garden experiment [C: Col0, K: Ksk1, W: Ws0], and the Tübingen wild sites: ERG, EY, JUG, PFN, WH [F: Fall, S: Spring]. (TIF) [file pbio.1002352.s007.tif]

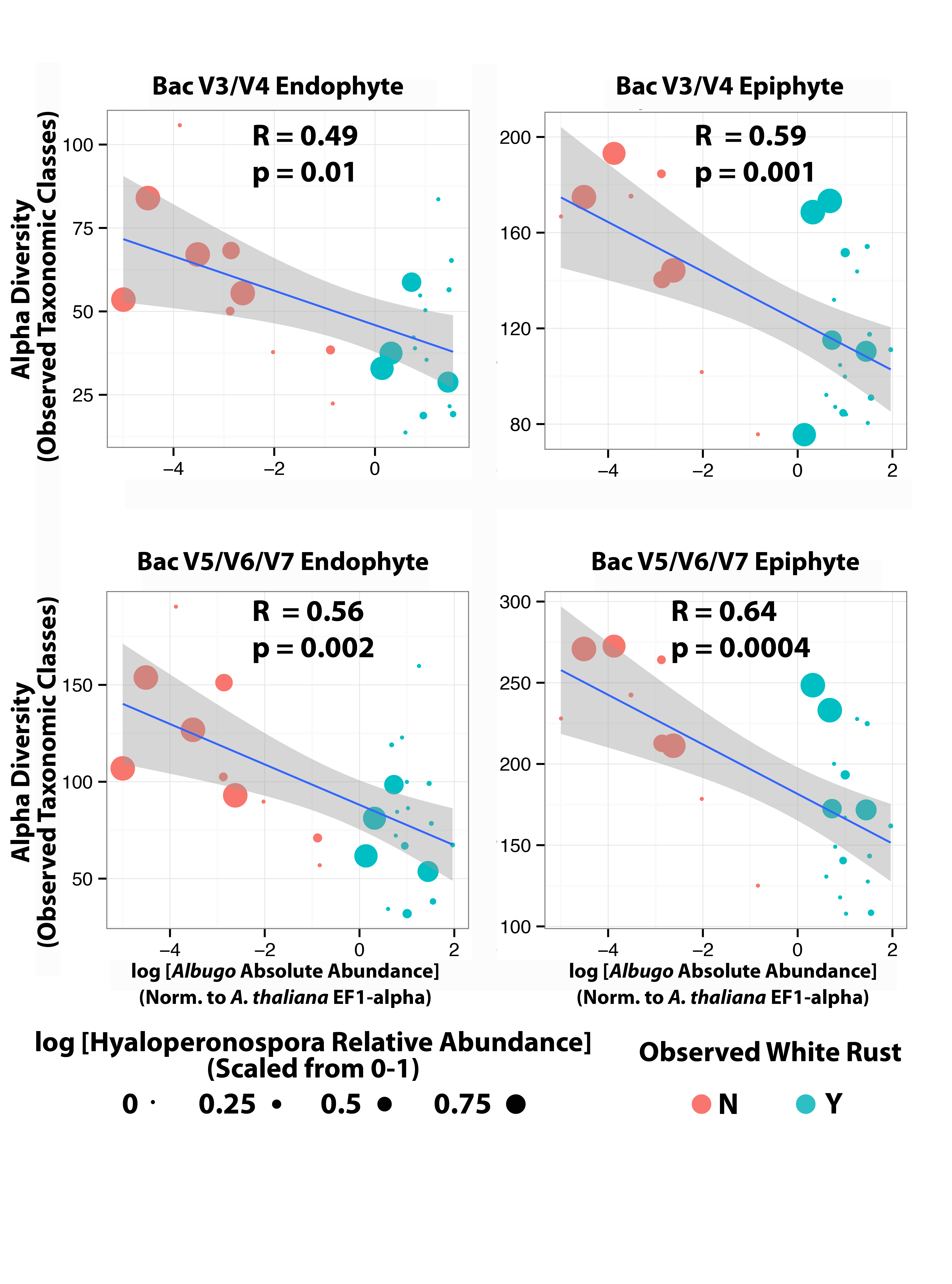

Supplement: S7 Fig — Plotted points are scaled to measured endophytic Hpa (based on the log of the average of ITS1 and ITS2 datasets relative abundance information. Relative abundances were scaled from 0–1 before averaging so that information between the two datasets would be comparable). The color of the plotted points corresponds to whether or not “white rust” caused by Albugo sp. was observed on the sample. (TIF) [file pbio.1002352.s008.tif]

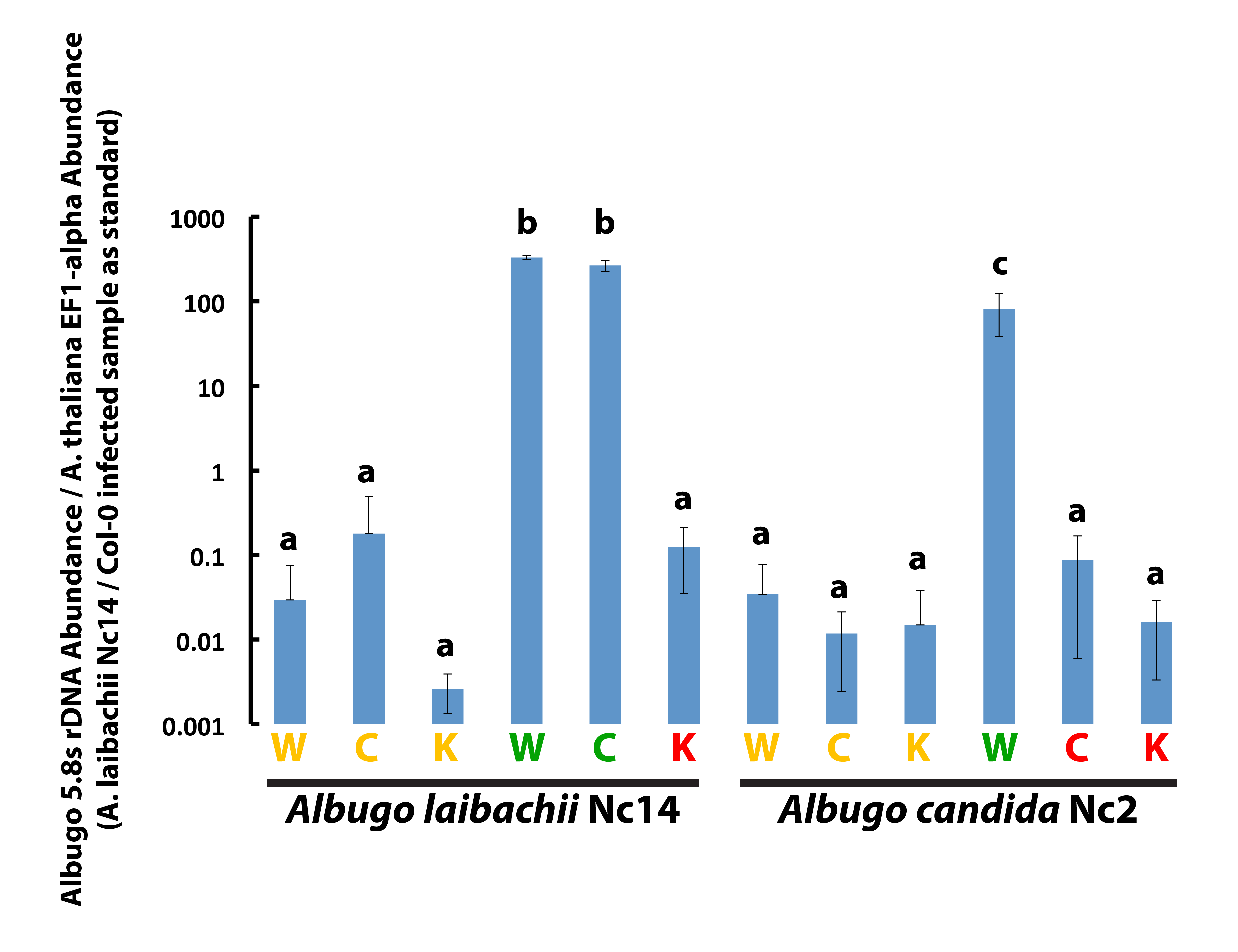

Supplement: S8 Fig — W: A. thaliana Ws-0, C: A. thaliana Col-0, K: A. thaliana Ksk-1. Yellow: Albugo sp. spores physically removed from inoculum by filtering, Green: Susceptible plants inoculated with Albugo sp. and associated microbes, Red: Resistant plants inoculated with Albugo sp. and associated microbes. (TIF) [file pbio.1002352.s009.tif]

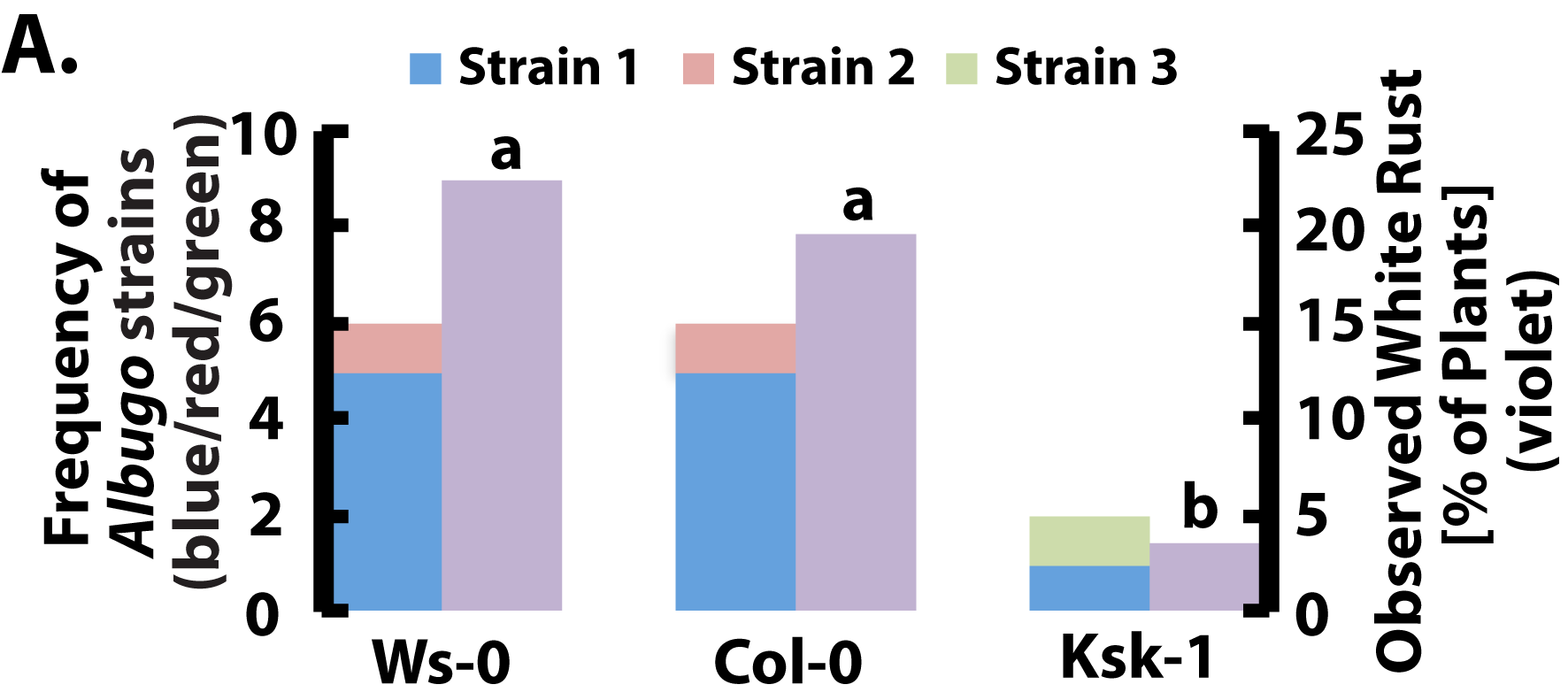

Supplement: S9 Fig — The A. thaliana accession Ksk-1 carries an allele for resistance to Albugo sp., which lent partial resistance to the wild pathogen strains. Letters indicate significance at p < 0.1 (Tukey’s HSD). (TIF) [file pbio.1002352.s010.tif]

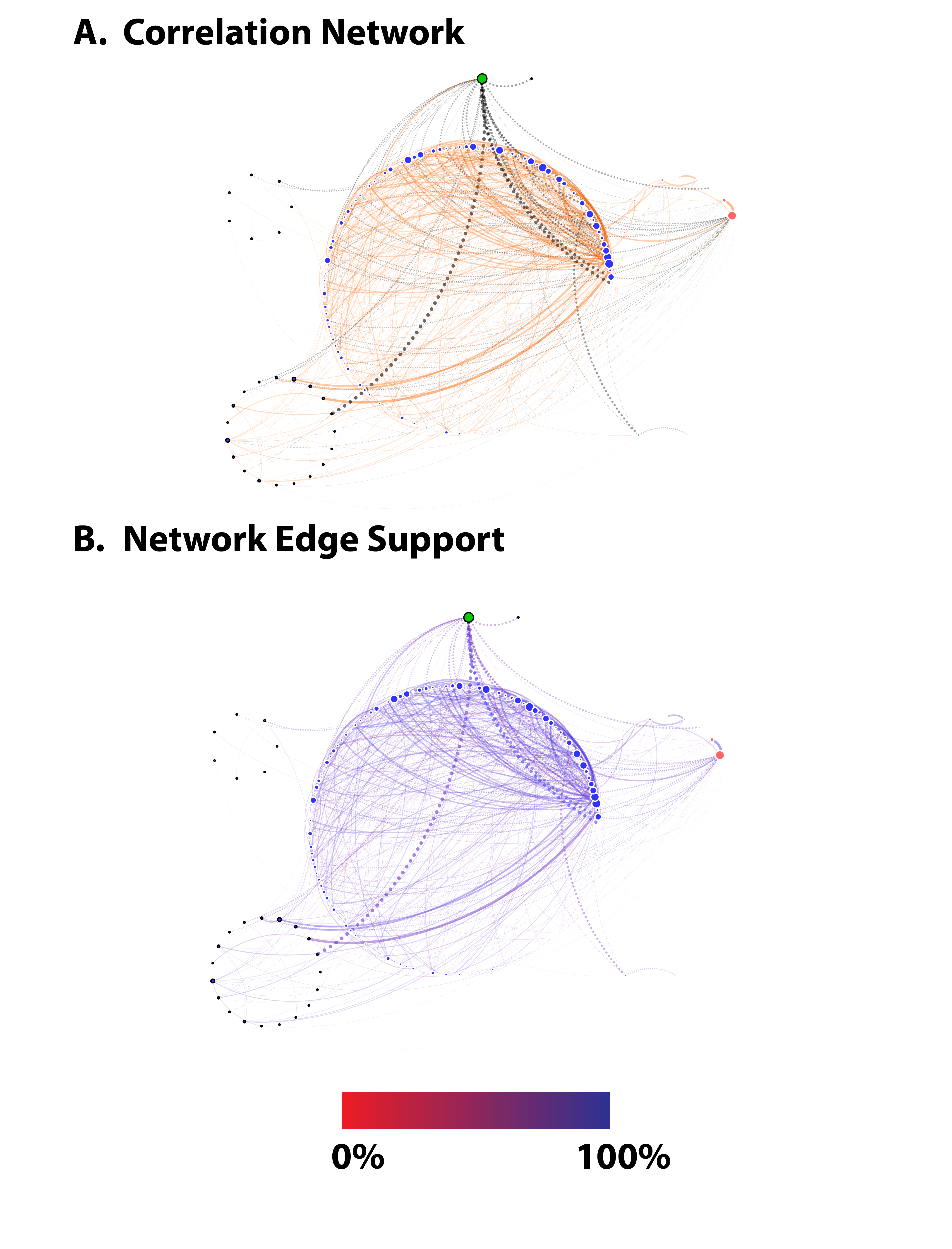

Supplement: S10 Fig — A. The correlation network, which is identical to that presented in Fig 2 in the main text. B. Edge support in the correlation network determined by randomly subsampling 50% of the data for each correlation 100 times. (TIF) [file pbio.1002352.s011.tif]

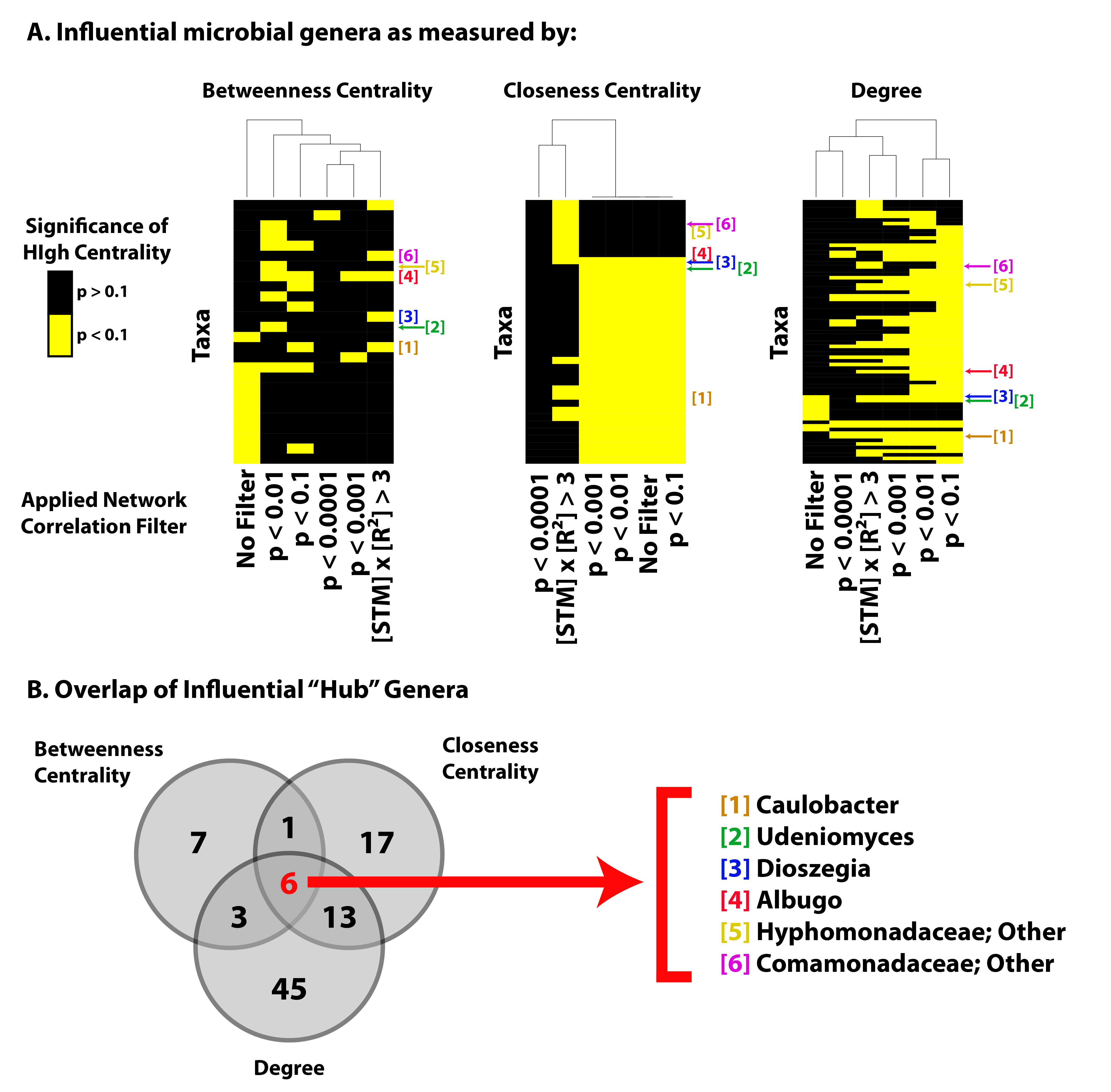

Supplement: S11 Fig — A. Comparison of the effects of different cutoffs for edge filtering (removing weak correlations between taxa) for three measures of node (taxa) centrality, where yellow highlights taxa that are significantly more central in the network using a specific cutoff. ratio of abundance sum to maximum abundance (STM) x R2 > 3 is a cutoff we designed that accounts for distribution of the microbes and the strength of the correlation (see Supplementary Materials and Methods [S1 Text] for details). Genera that were detected with any one of the filters were considered a possible “hub” microbe. B. Only taxa that were discovered using all three metrics were considered as likely “hub” taxa (see S7 Table for other taxonomic levels). Genus-level “hub” taxa are highlighted in A. (TIF) [file pbio.1002352.s012.tif]

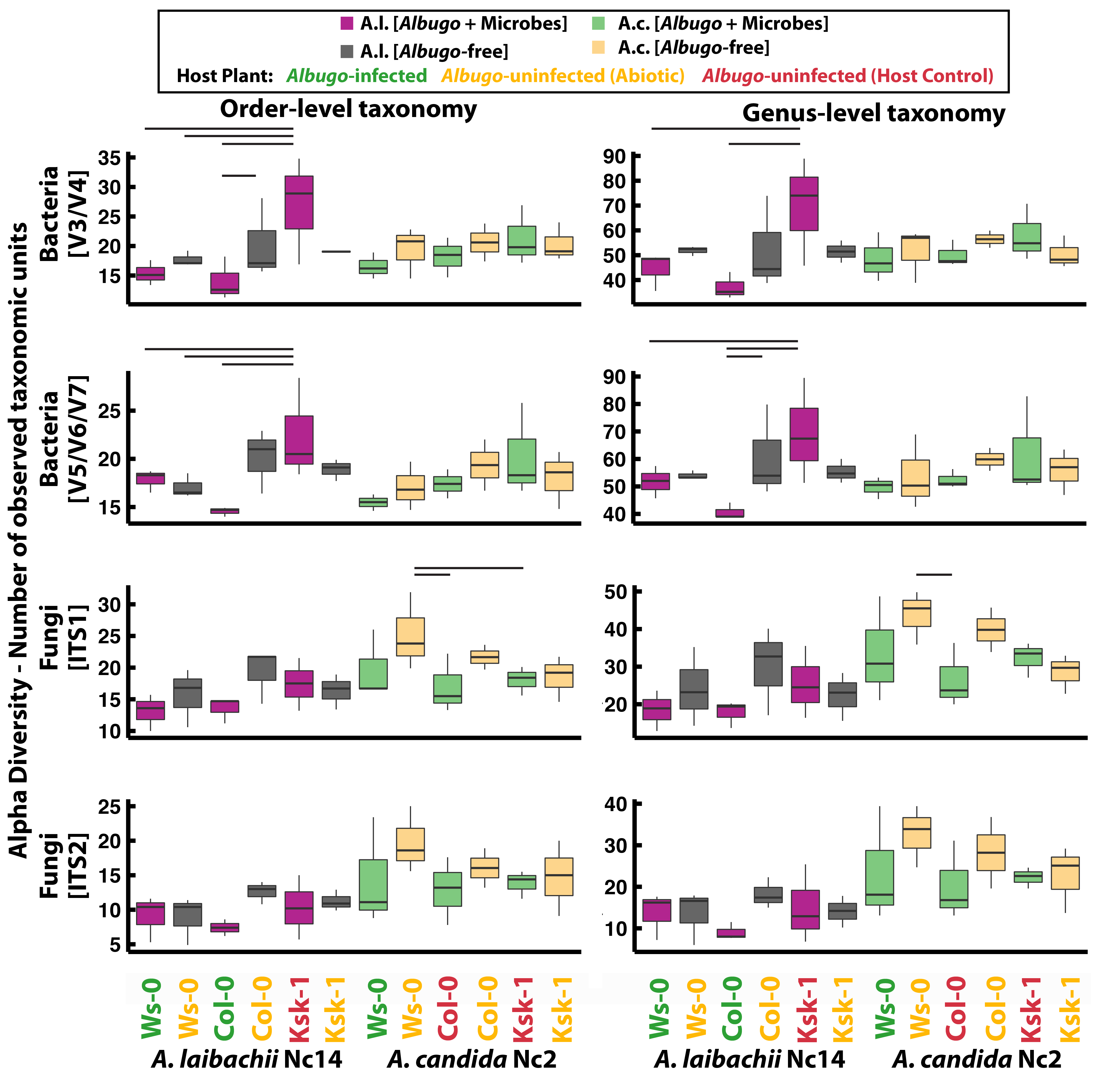

Supplement: S13 Fig — Results demonstrate that Albugo-infected plants had significantly lower bacterial diversity than controls (lines indicate t test, p < 0.05). Key: Ws-0: A. thaliana Ws-0, Col-0: A. thaliana Col-0, Ksk-1: A. thaliana Ksk-1. Green: Susceptible hosts, Red: Resistant hosts, Yellow: Filter removal of Albugo on all hosts. (TIF) [file pbio.1002352.s014.tif]

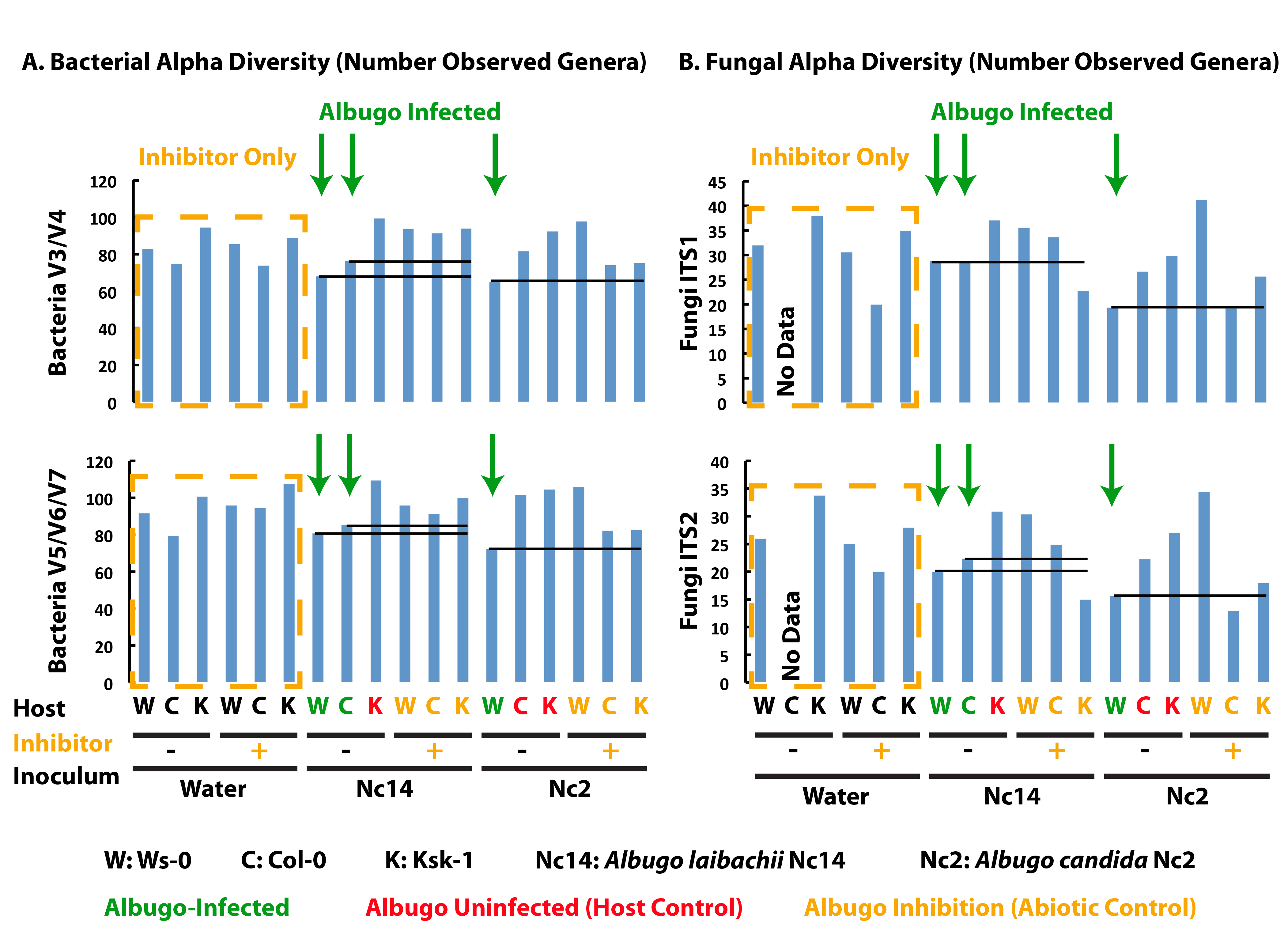

Supplement: S14 Fig — A strong suppressive effect on bacterial alpha diversity by Albugo sp. was confirmed regardless of the mechanism of removal of Albugo (see Fig 4 and S13 Fig for removal of Albugo spores by filtering). Solid black lines are placed to facilitate comparison of control samples to Albugo-infected samples. W: A. thaliana Ws-0, C: A. thaliana Col-0, K: A. thaliana Ksk-1. Green: Susceptible hosts, Red: Resistant hosts, Yellow: Chemical inhibition of Albugo on all hosts by metalaxyl and benalaxyl. (TIF) [file pbio.1002352.s015.tif]

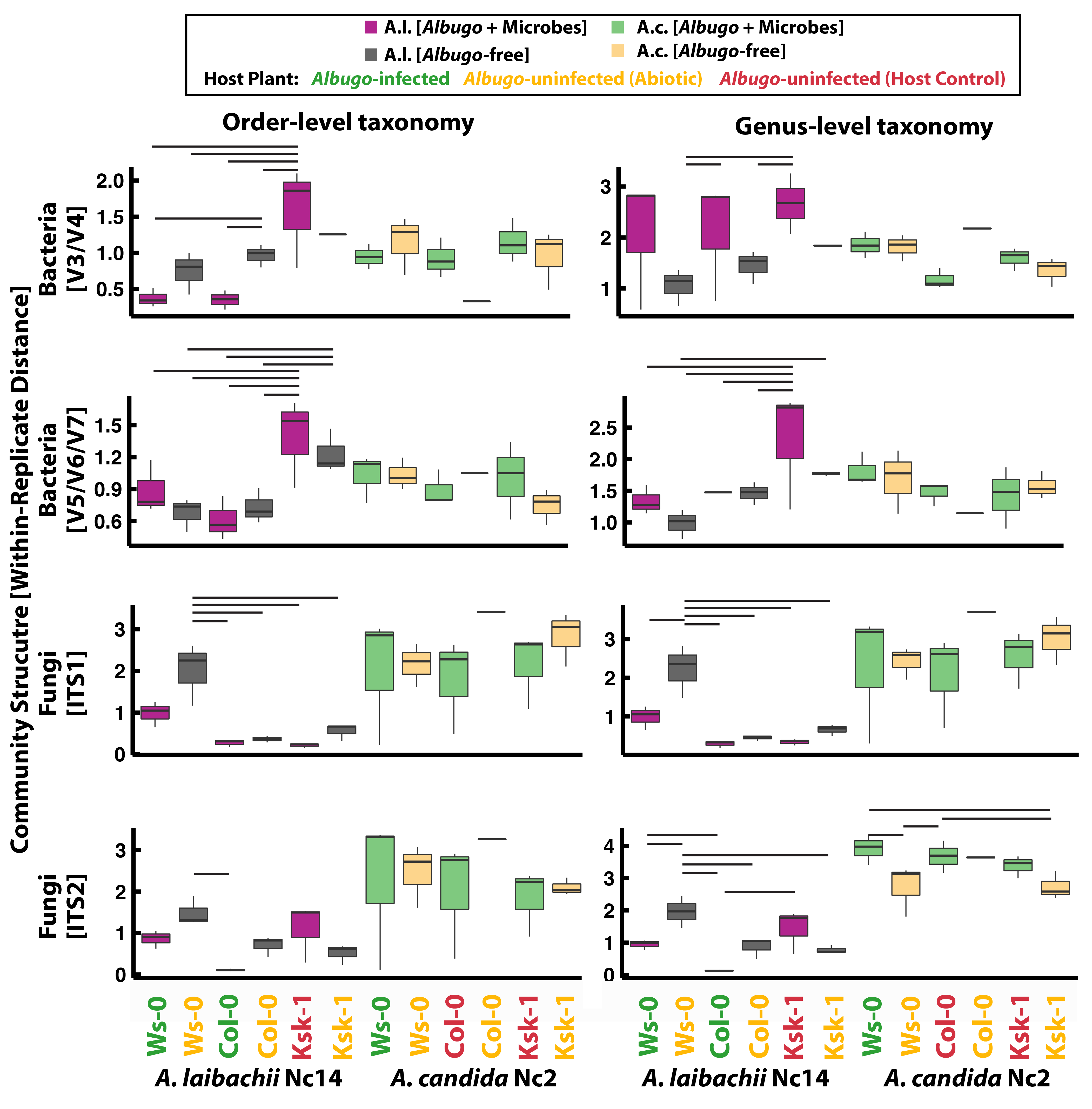

Supplement: S15 Fig — Results demonstrate that the final microbial communities recovered from Albugo-infected plants were more similar between experimental replicates than controls (lines indicate t test, p < 0.05). Key: Ws-0: A. thaliana Ws-0, Col-0: A. thaliana Col-0, Ksk-1: A. thaliana Ksk-1. Green: Susceptible hosts, Red: Resistant hosts, Yellow: Filter removal of Albugo on all hosts. (S1_Data.xlsx) (TIF) [file pbio.1002352.s016.tif]

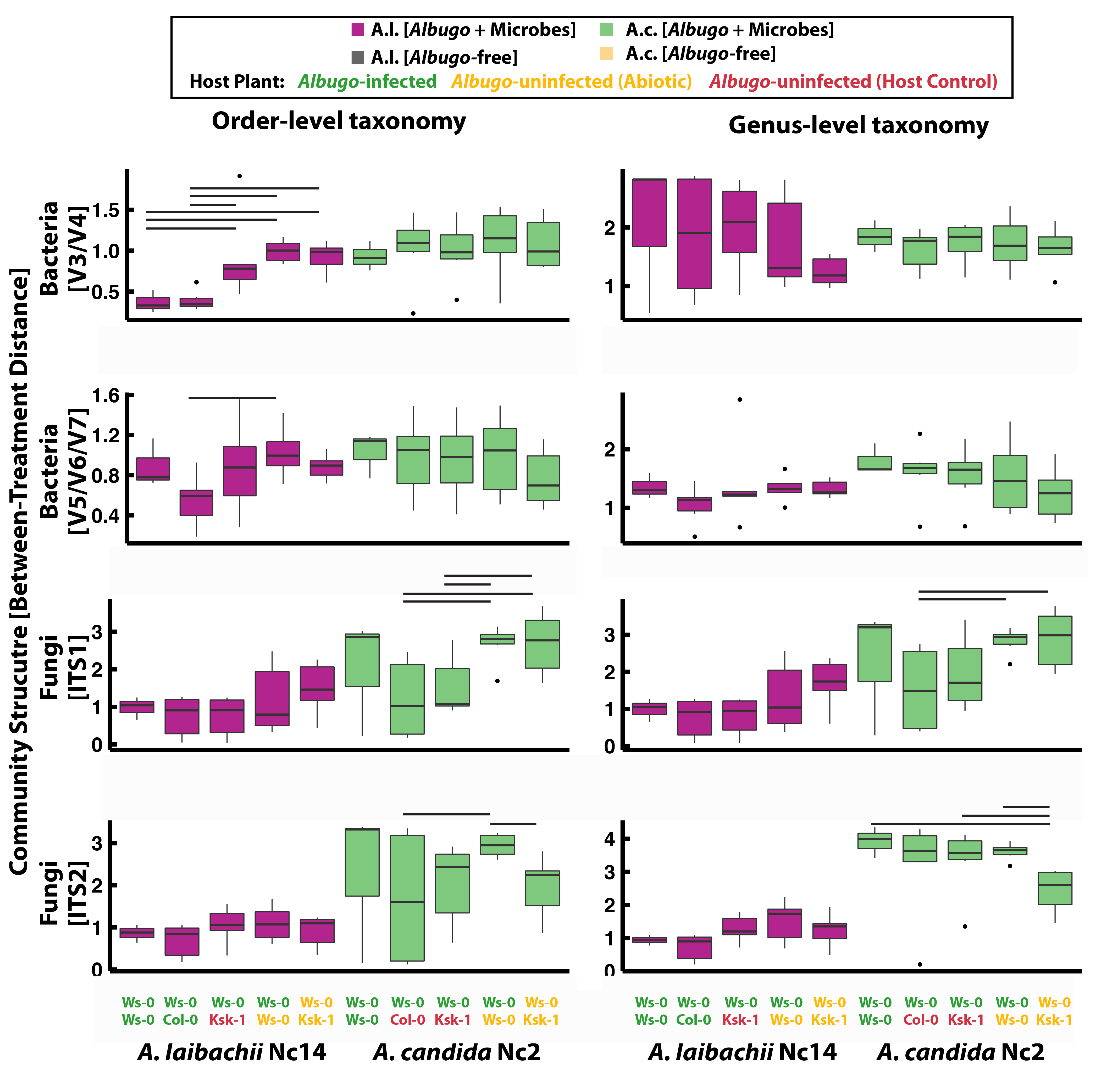

Supplement: S16 Fig — Results demonstrate that bacterial communities at the order level from Albugo-infected A. thaliana was more similar to other infected plant communities than to uninfected controls (lines indicate t test, p < 0.05). For simplicity, not every possible comparison is shown. Key: Ws-0: A. thaliana Ws-0, Col-0: A. thaliana Col-0, Ksk-1: A. thaliana Ksk-1. Green: Susceptible hosts, Red: Resistant hosts, Yellow: Filter removal of Albugo on all hosts. (TIF) [file pbio.1002352.s017.tif]

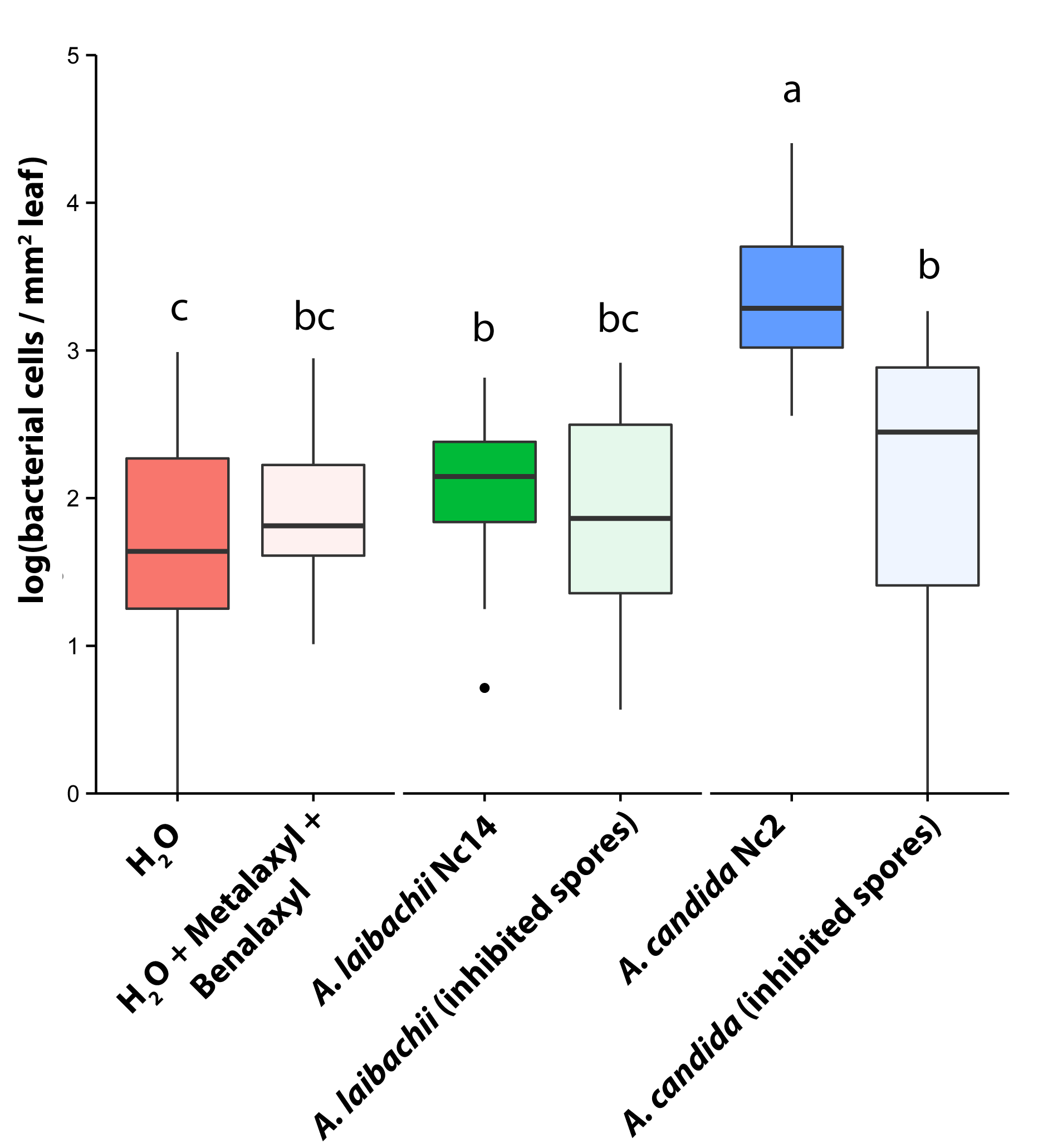

Supplement: S17 Fig — Results demonstrate high counts of bacteria on A. thaliana leaves infected with Alb. candida Nc2 (t test, p < 0.05). Controls with inhibited spores used the oomycete-specific inhibitory compounds metalaxyl and benalaxyl. (TIF) [file pbio.1002352.s018.tif]

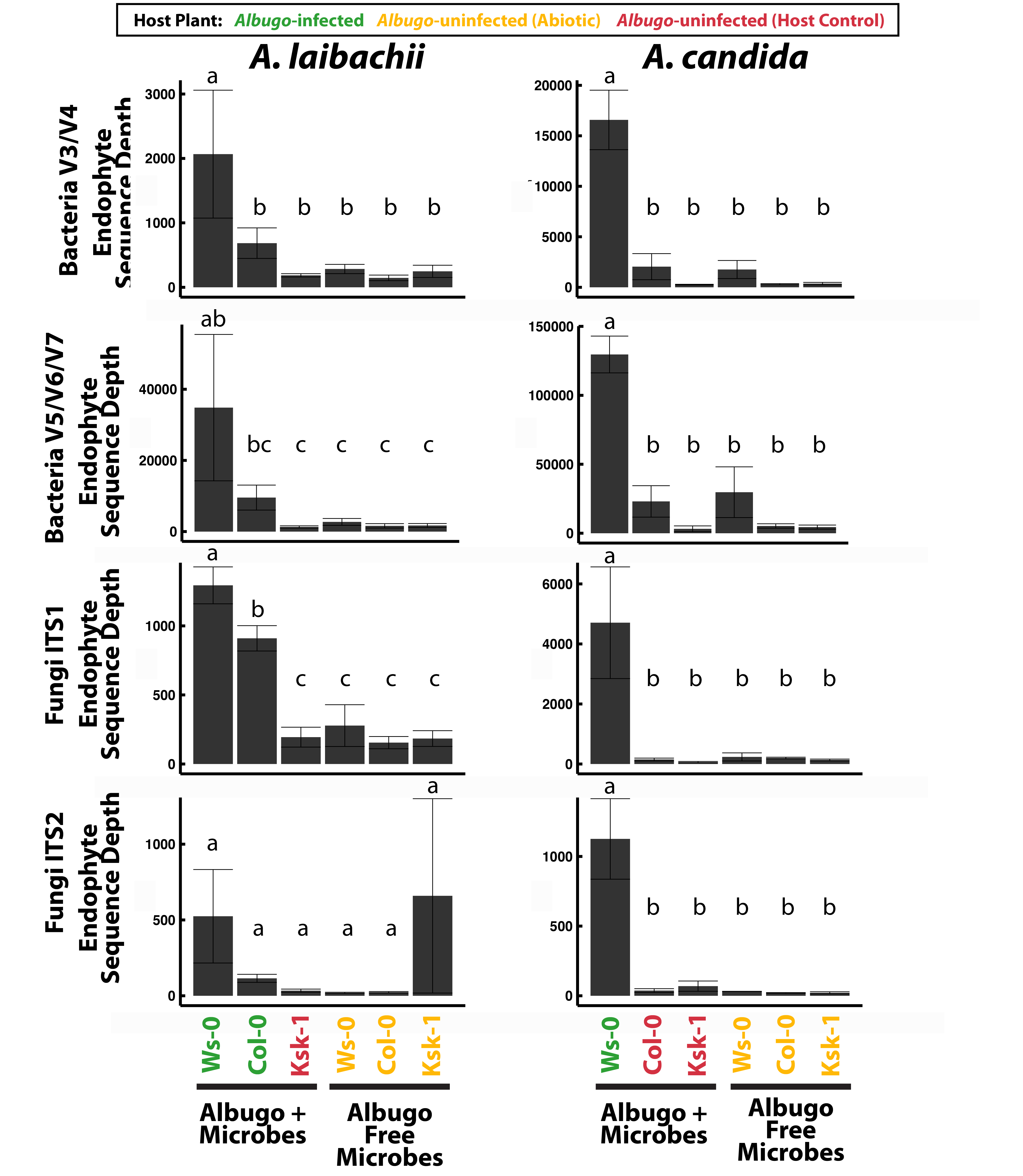

Supplement: S18 Fig — For this analysis, bacterial reads identified as cyanobacteria at only the phylum level (with no more specific taxonomic assignment) have been removed. Indicated significant differences are based on a t test with p < 0.05. (TIF) [file pbio.1002352.s019.tif]

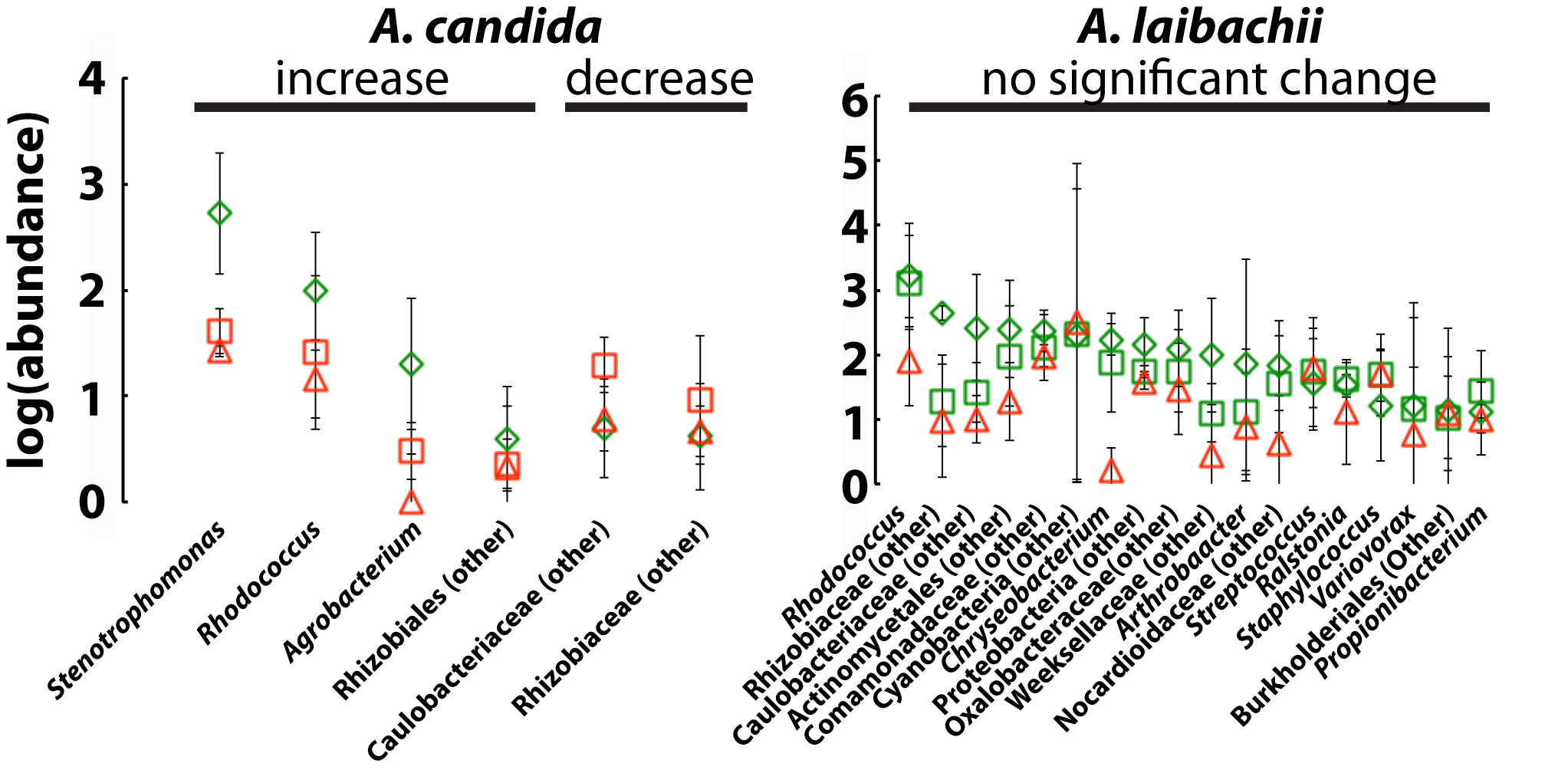

Supplement: S19 Fig — A. Genera with significant increase or decrease (t test, p < 0.05) on plants infected with Alb. candida compared to both controls. B. Genera without statistically significant (t test, p < 0.05) enrichment on two host accessions infected with Alb. laibachii. Some abundant taxa were not enriched due to Alb. laibachii infection, while others were only enriched on Alb. thaliana Ws-0 (the accession on which stock infections were kept) or were similar abundance in all treatments. (TIF) [file pbio.1002352.s020.tif]

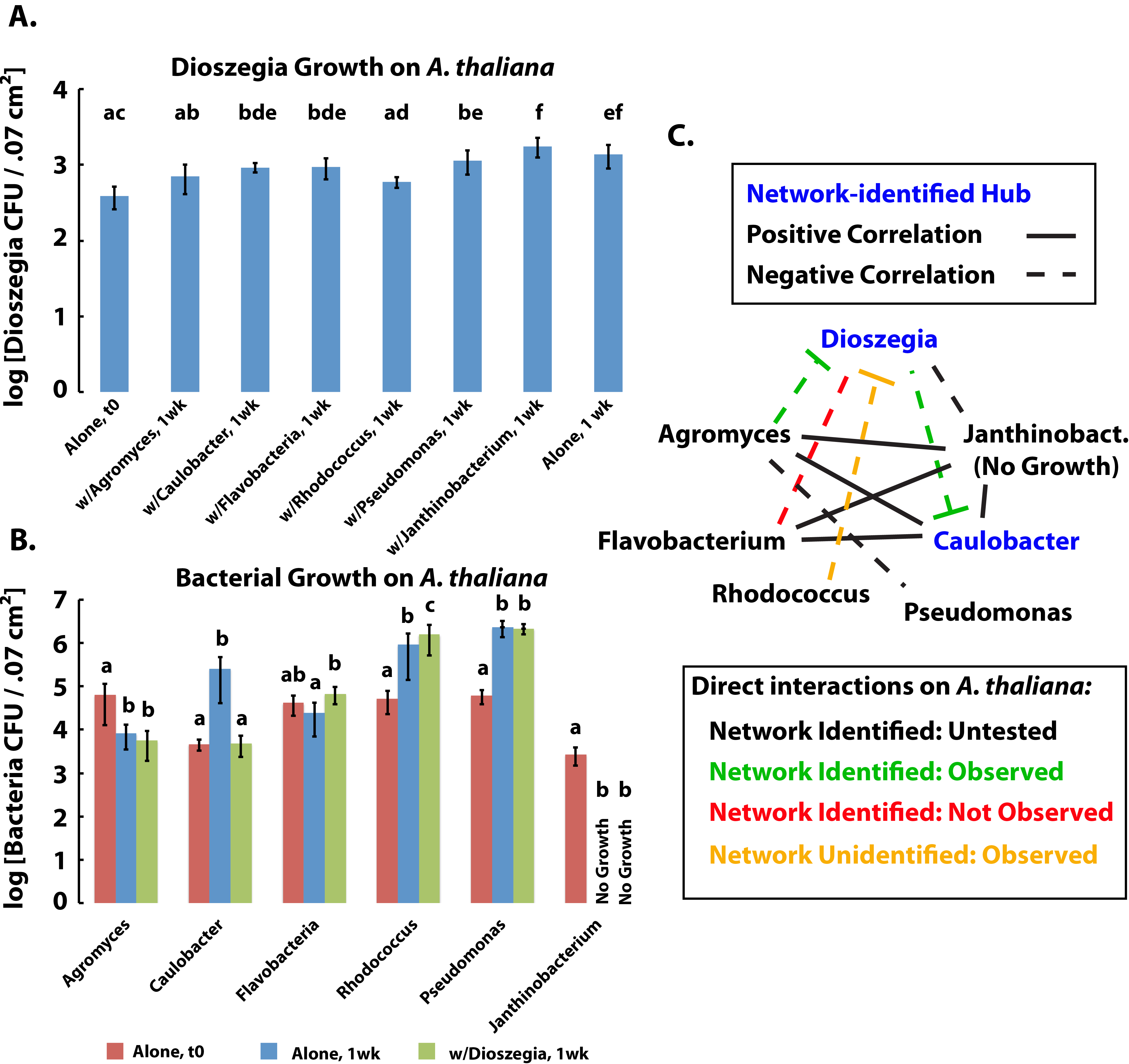

Supplement: S20 Fig — A. Dioszegia growth with bacteria (letters indicate significant differences at Tukey’s HSD p < 0.05). Dioszegia growth was significantly negatively affected by Rhodococcus sp. and Agromyces sp. compared to growth alone. B. Bacterial growth alone or with Dioszegia (letters indicate significant differences with Tukey’s HSD p < 0.05 only within each bacterial isolate test, not between isolates). The growth of Rhodococcus sp. was slightly increased in the presence of Dioszegia compared to growth alone. The strongest effect was on Caulobacter sp. where strong growth was completely inhibited by Dioszegia sp. Details about bacterial isolates are provided in S8 Table. C. Observed interactions confirmed and gave direction to several correlations observed in our network analysis (green) and others (red) could be a result of indirect connections (black). Rhodococcus sp. interactions were not observed in the network analysis, but this was a lab isolate and the genus was very low abundance in the field. (TIF) [file pbio.1002352.s021.tif]

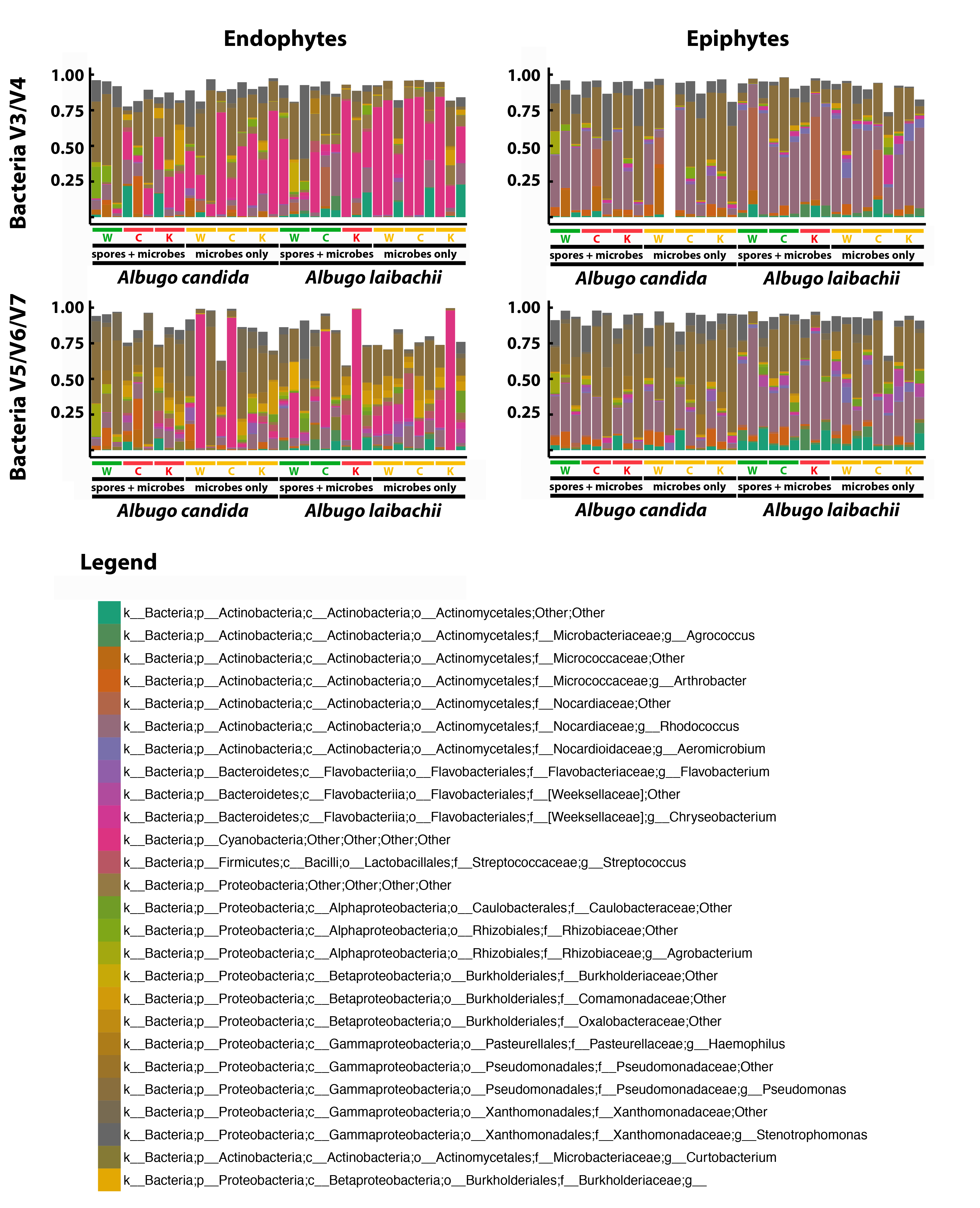

Supplement: S21 Fig — Legends are common for the barcharts in the figure. Data is based on relative abundance calculated from data that was not first subsampled. Key: Spores + microbes: inoculation of Albugo sp. and associated microorganisms, Microbes only: inoculation of associated microorganisms after filter removal of Albugo sp., W: A. thaliana Ws-0, C: A. thaliana Col-0, K: A. thaliana Ksk-1, Green: Susceptible host, Red: Resistant host, Yellow: filter removal of Albugo sp. on all hosts. (TIF) [file pbio.1002352.s022.tif]

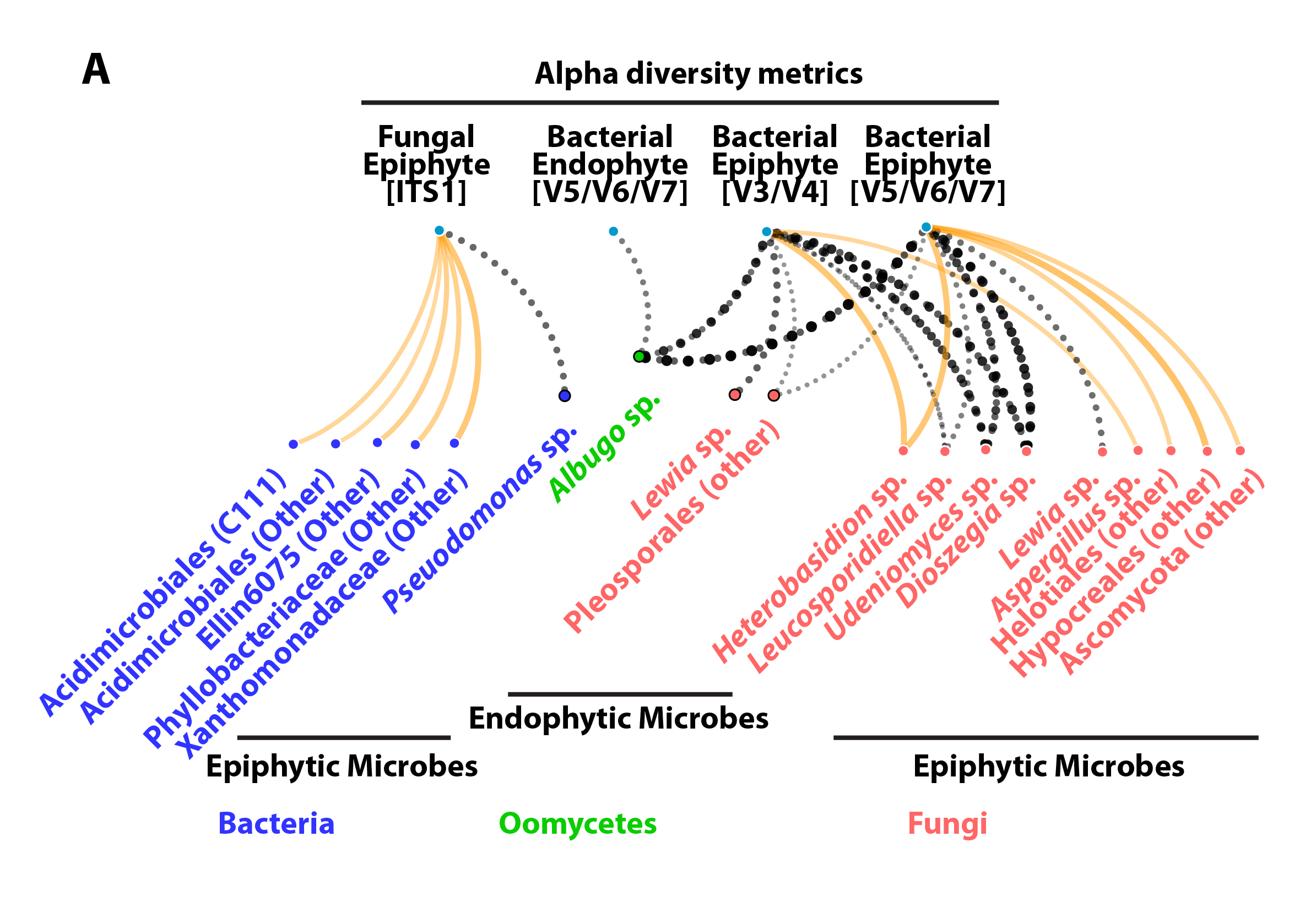

Supplement: S22 Fig — Interkingdom correlations reveal several genera correlated significantly to fungal and bacterial epiphyte and endophyte diversity. Strong negative correlations of Albugo sp. and Dioszegia sp. reinforces their putative role based on paired microbe correlations in limiting abundance of many epiphytic bacterial genera. (TIF) [file pbio.1002352.s023.tif]

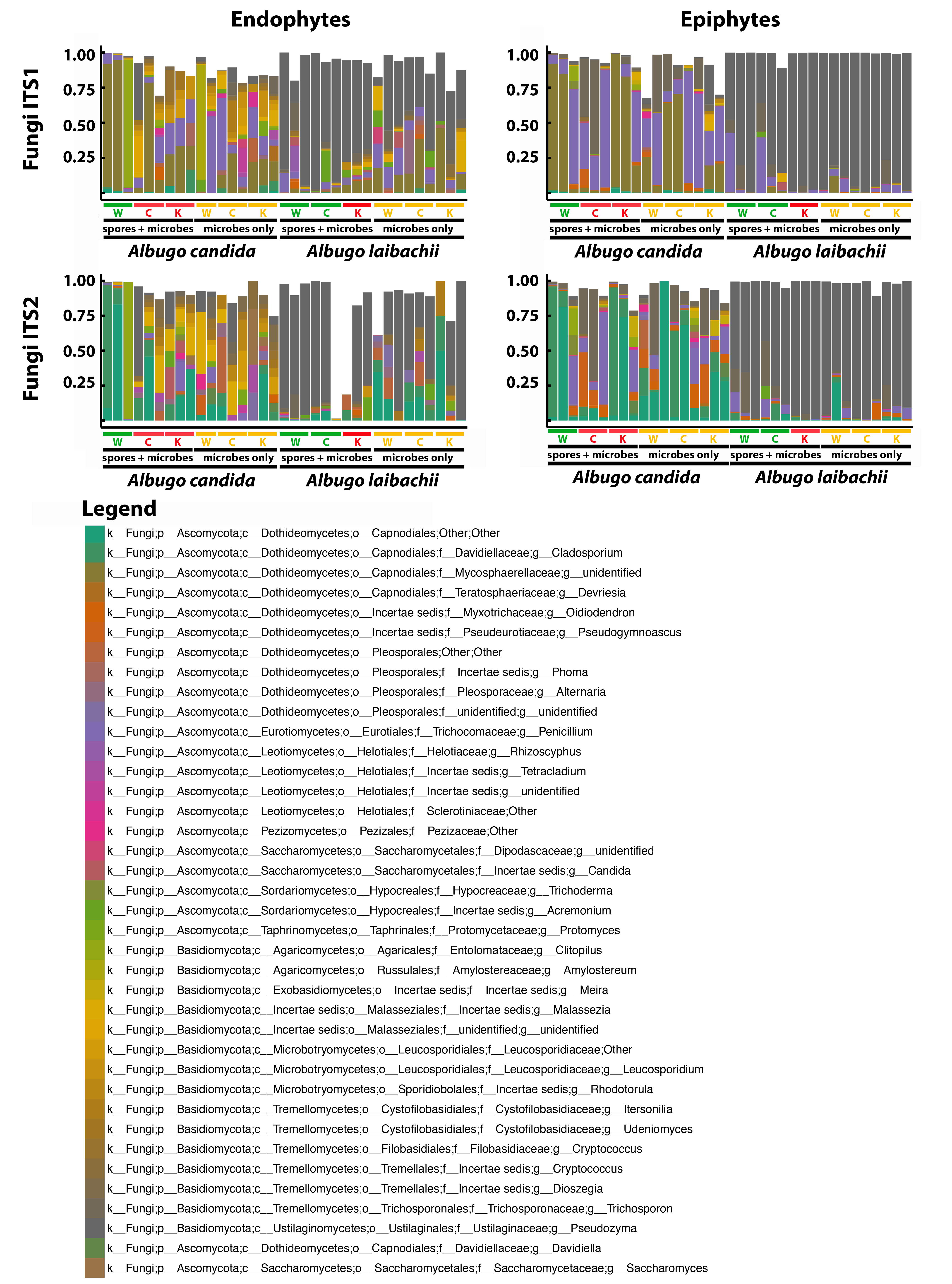

Supplement: S23 Fig — Legends are common for the barcharts in the figure. Data is based on relative abundance calculated from data that was not first subsampled. Key: Spores + microbes: inoculation of Albugo sp. and associated microorganisms, Microbes only: inoculation of associated microorganisms after filter removal of Albugo sp., W: A. thaliana Ws-0, C: A. thaliana Col-0, K: A. thaliana Ksk-1, Green: Susceptible host, Red: Resistant host, Yellow: filter removal of Albugo sp. on all hosts. (TIF) [file pbio.1002352.s024.tif]

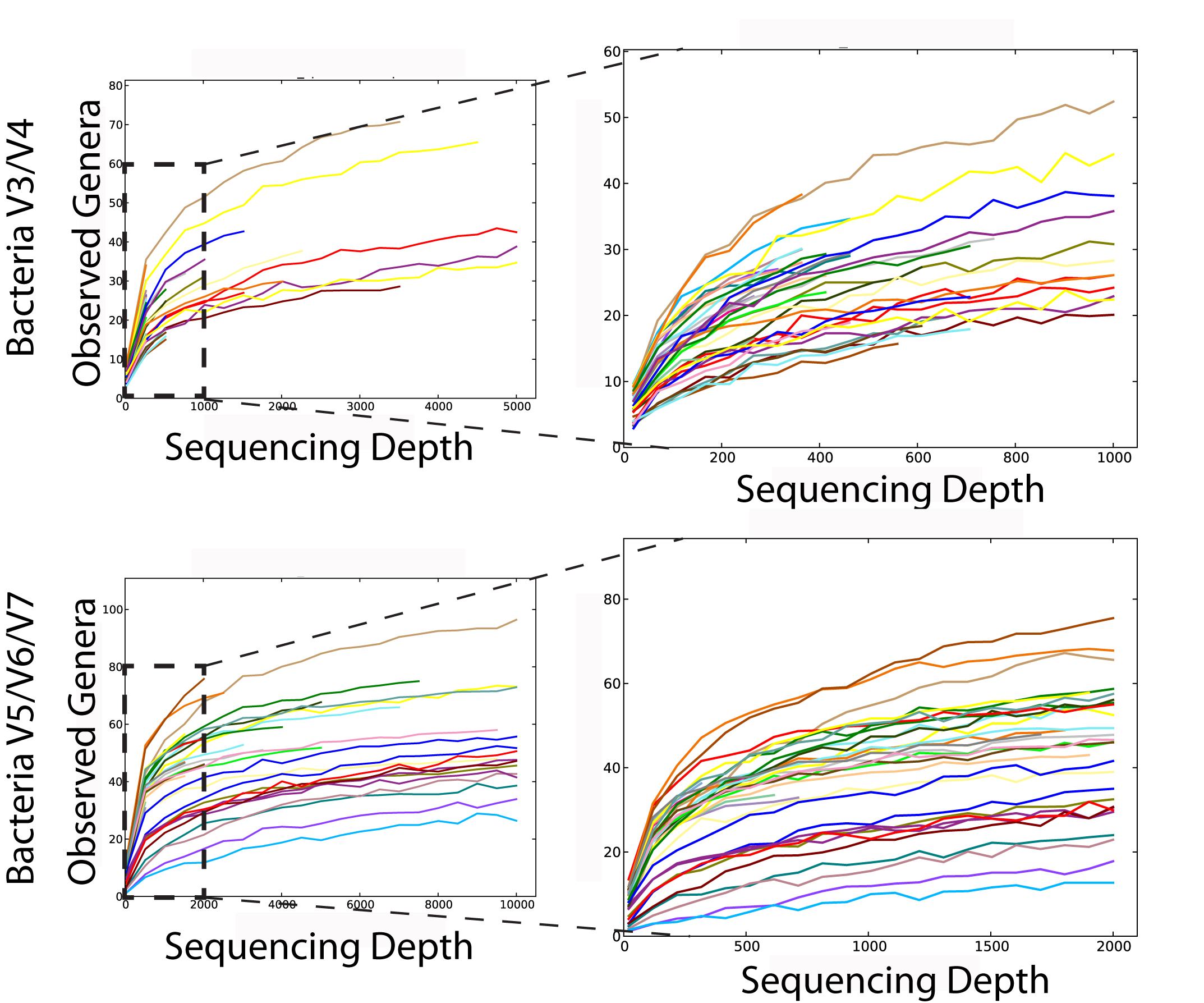

Supplement: S24 Fig — This effect was especially apparent in the V5/V6/V7 region where sampling was deepest. Data is based on observed number of bacterial genera and each line represents one sample. (TIF) [file pbio.1002352.s025.tif]
