# Supplementary material for: Microbial Hub Taxa Link Host and Abiotic Factors to Plant Microbiome Variation
Source: PLoS Biol. 2016 Jan 20;14(1):e1002352. doi: 10.1371/journal.pbio.1002352 (PMC4720289; doi:10.1371/journal.pbio.1002352)
Supplement: S12 Fig — Experiments were complemented with two different types of controls for the experimental infected A. thaliana. The first control were Albugo-free containing microorganisms that were associated with each Albugo strain but no Albugo spores. This control represents an abiotic factor, such as a distribution limitation, that in nature would limit the growth of Albugo. We tested both filter removal of Albugo spores (see Fig 4 and S13, S15 and S16 Figs) and chemical Albugo inhibition (see S14 Fig). The second control was resistant accessions (Ksk1 for Nc14 and Col0/Ksk1 for Nc2) that were inoculated with Albugo and its associated microbial community. This control represents a host factor, resistance, that in nature would limit the growth of Albugo. (PDF) [file pbio.1002352.s013.pdf]

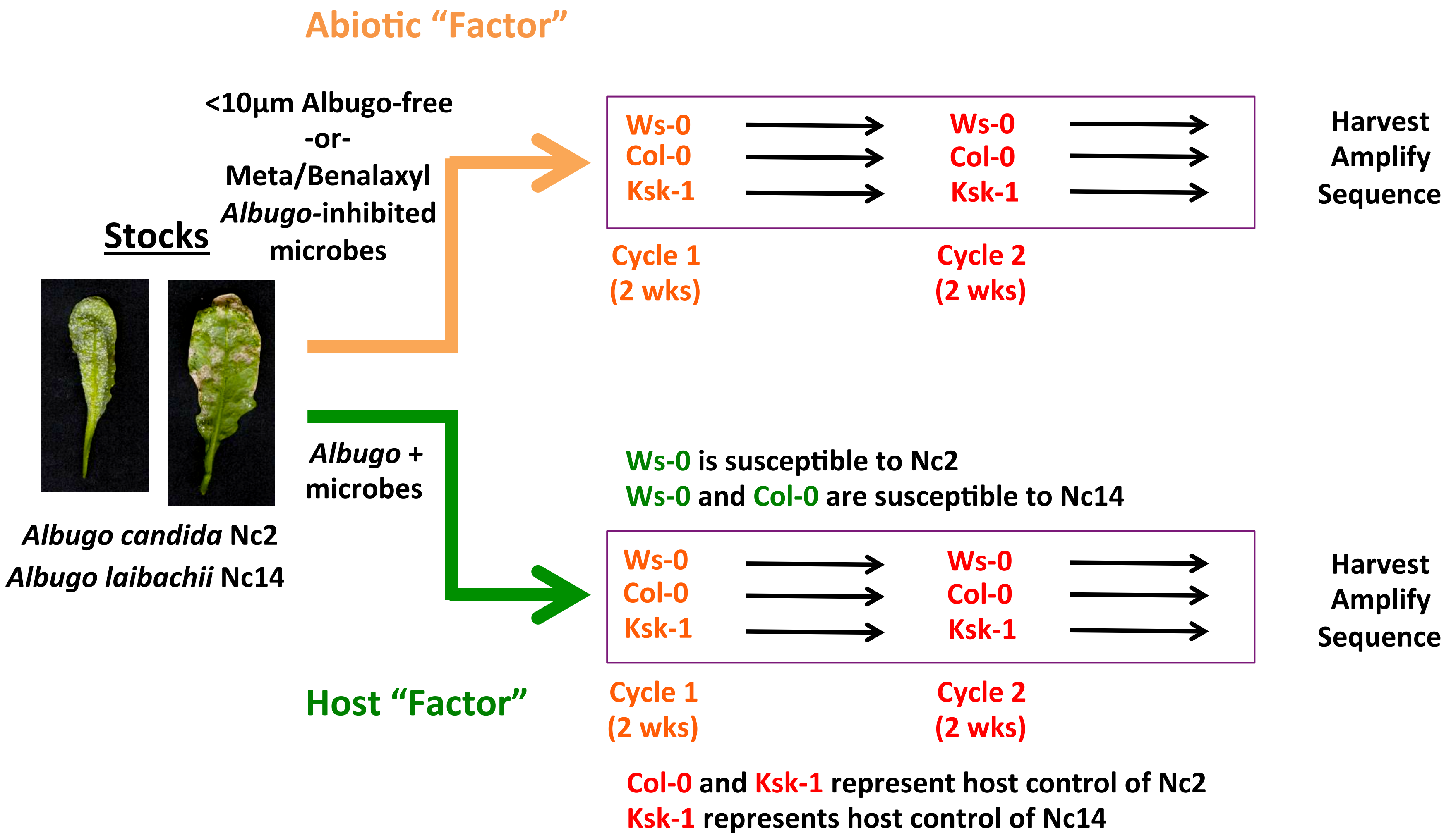
Abiotic "Factor"
<10µm Albugo-free
-or-
Meta/Benalaxyl
Albugo-inhibited
microbes
Stocks
Albugo candida Nc2
Albugo laibachii Nc14
Albugo +
microbes
Host "Factor"
Ws-0
Col-0
Ksk-1
Cycle 1
(2 wks)
Ws-0
Col-0
Ksk-1
Cycle 2
(2 wks)
Harvest
Amplify
Sequence
Ws-0 is susceptible to Nc2
Ws-0 and Col-0 are susceptible to Nc14
Ws-0
Col-0
Ksk-1
Cycle 1
(2 wks)
Ws-0
Col-0
Ksk-1
Cycle 2
(2 wks)
Harvest
Amplify
Sequence
Col-0 and Ksk-1 represent host control of Nc2
Ksk-1 represents host control of Nc14
