# Supplementary material for: Microbial Hub Taxa Link Host and Abiotic Factors to Plant Microbiome Variation
Source: PLoS Biol. 2016 Jan 20;14(1):e1002352. doi: 10.1371/journal.pbio.1002352 (PMC4720289; doi:10.1371/journal.pbio.1002352)
Supplement: S1 Table — (DOCX) [file pbio.1002352.s028.docx]

**S1 Table**

|  | **Coordinates** | |  | **Linear Distances (km)^1^** | | | | | | |  |
| --- | --- | --- | --- | --- | --- | --- | --- | --- | --- | --- | --- |
| **Site** | **N Coord.** | **E Coord.** | **Type** | **WH** | **ERG** | **JUG** | **EY** | | **PFN** | |  |
| **WH** | 48^o^ 30' 24.62" | 8^o^ 56' 12.81" | Urban |  | 9.47 | 15.61 | | 13.21 | | 14.06 | |
| **ERG** | 48^o^ 29' 43.30" | 8^o^ 48' 34.95" | Urban | 9.47 |  | 24.92 | | 5.82 | | 23.26 | |
| **JUG** | 48^o^ 33' 24.30" | 9^o^ 08' 05.00" | Rural | 15.61 | 24.92 |  | | 28.71 | | 1.93 | |
| **EY** | 48^o^ 26' 46.30" | 8^o^ 46' 59.10" | Railroad | 13.21 | 5.82 | 28.71 | |  | | 27.24 | |
| **PFN** | 48^o^ 33' 39.30" | 9^o^ 06' 33.60" | Rural | 14.06 | 23.26 | 1.93 | | 27.24 | |  | |
| **CG** | 50^o^ 57' 21.56" | 6^o^ 51' 40.20" | Rural |  |  |  | |  | |  | |

^1^Linear distances are provided between the Tübingen wild sampling sites between which direct comparisons of community similarity can be made based on Fig 1 and S1Fig.
