# Supplementary material for: Microbial Hub Taxa Link Host and Abiotic Factors to Plant Microbiome Variation
Source: PLoS Biol. 2016 Jan 20;14(1):e1002352. doi: 10.1371/journal.pbio.1002352 (PMC4720289; doi:10.1371/journal.pbio.1002352)
Supplement: S2 Table — (DOCX) [file pbio.1002352.s029.docx]

**S2 Table**

| **Date** | **Sample** | **Site** | **Pooled Plants?^1^** | **Arabidopsis Strain/**  **Accession^2^** | **Observed Albugo^3^** | **Measured Albugo**  **(Relative-scaled)^4^** | **Measured Oomycete (Absolute)^5^** | **Measured Albugo**  **(Absolute)^6^** | ***A. laibachii* Strain^7^** | ***A. candida?^8^*** | **Sequencing Run^7^** |
| --- | --- | --- | --- | --- | --- | --- | --- | --- | --- | --- | --- |
| **11/26/13** | **1.1 S2** | WH | Y | WH1 | Uninfected | NA |  |  | Not Detected | No | Not Sequenced |
|  | **1.2 S1** | WH | Y | WH2 | Infected | 0.889884113 | 51.2278 | 29.35 | WH1 | No | G |
|  | **1.2 S2** | WH | Y | n/a | Uninfected | 0.145508953 | 0.3424 | 1.34E-03 | Not Detected | No | G |
|  | **S1** | ERG | Y | ERG2 | Infected | 0.997036545 | 18.3116 | 17.98 | ERG1 | No | G |
|  | **S2** | ERG | Y | ERG1 | Uninfected | 0.996550939 | 0.1338 | 0.13 | ERG1 | No | G |
|  | **S3** | ERG | Y | ERG1 | Infected | NA |  |  | ERG1 | No | Not Sequenced |
|  | **S4** | ERG | Y | ERG1 | Uninfected | NA |  |  | Not Detected | No | Not Sequenced |
|  | **S1** | EY | Y | EY1 | Uninfected | NA |  |  | Not Detected | No | Not Sequenced |
|  | **S2** | EY | Y | EY1 | Infected | 0.641683103 | 5.6303 | 2.09 | EY1 | No | G |
|  | **S3** | EY | Y | EY1 | Uninfected | 0.339287638 | 0.0004 | 1.01E-05 | Not Detected | No | G |
|  | **S4** | EY | Y | EY1 | Uninfected | 0.135578396 | 0.0772 | 3.04E-04 | Not Detected | No | G |
|  | **S2** | JUG | Y | JUG2 | Uninfected | 0.094103887 | 0.0467 | 1.34E-04 | Not Detected | No | G |
|  | **S5** | JUG | Y | JUG1 | Infected | 0.915442472 | 7.4205 | 4.74 | JUG1 | No | G |
|  | **S1** | PFN | Y | Mixed | Uninfected | 0 | 0.0265 | 3.1E-05 | Not Detected | No | G |
| **5/7/13** | **S1** | WH | Y | WH1 | Infected | 0.999843138 | 32.7676 | 32.75 | WH1 | No | C |
|  | **S2** | WH | Y | WH1 | Infected | 0.774392223 | 4.0118 | 1.37 | WH1 | No | C |
|  | **S3** | ERG | Y | ERG1 | Infected | 0.959600198 | 6.9199 | 5.34 | ERG1 | No | D |
|  | **S4** | ERG | Y | ERG1 | Infected | 0.999598844 | 9.1256 | 9.11 | ERG1 | No | D |
|  | **S5** | JUG | N | JUG1 | Infected | NA |  |  | JUG1 | No | Not Sequenced |
|  | **S6** | JUG | N | JUG1 | Infected | NA |  |  | JUG1 | Yes^9^ | Not Sequenced |
|  | **s8** | JUG | N | JUG1 | Infected | 0.986962084 | 6.3655 | 5.83 | JUG1 | No | D |
|  | **s9** | JUG | N | JUG1 | Infected | 0.887636779 | 17.2767 | 10.11 | JUG1 | No | D |
|  | **s10** | JUG | N | *Capsella* sp. | Infected | NA |  |  | *A. condida* | Yes | Not Sequenced |
|  | **S11** | EY | Y | EY1 | Uninfected | NA |  |  | Not Detected | No | Not Sequenced |
|  | **S12** | EY | N | EY1 | Uninfected | 0.293872377 | 0.2144 | 2.38E-03 | Not Detected | No | E |
|  | **S13** | EY | N | EY1 | Uninfected | NA |  |  | EY2 | No | Not Sequenced |
|  | **S14** | EY | N | EY1 | Infected | 0.999965258 | 91.9464 | 91.92 | EY1 | No | E |
|  | **S15** | EY | N | EY1 | Infected | 0.961120316 | 35.5693 | 28.08 | EY1 | No | E |
|  | **S16** | EY | N | EY1 | Uninfected | NA |  |  | Abn. Pattern | No | Not Sequenced |
|  | **S17** | PFN | N | PFN1 | Uninfected | NA |  |  | Not Detected | No | Not Sequenced |
|  | **S18** | PFN | N | PFN2 | Uninfected | 0.605427349 | 0.0058 | 1.38E-03 | Not Detected | No | E |
| **5/10/13** | **1** | CG | N | Ws-0 | Infected | 0.994702256 | 31.5113 | 30.26 | C1 | No | A/B |
|  | **2** | CG | N | Ws-0 | Infected | 0.999361273 | 10.0130 | 9.98 | C1 | No | A/B |
|  | **3** | CG | N | Ws-0 | Infected | 0.999161277 | 6.2338 | 6.21 | C1 | No | A/B |
|  | **4** | CG | N | Col-0 | Infected | 0.999053261 | 35.6621 | 35.46 | C1 | No | A/B |
|  | **5** | CG | N | Col-0 | Infected | 0.99977347 | 7.8712 | 7.87 | C1 | No | A/B |
|  | **6** | CG | N | Col-0 | Infected | 0.999441442 | 10.5477 | 10.54 | C1 | No | A/B |
|  | **7** | CG | N | Ksk-1 | Possible | 0.999326095 | 4.0235 | 4.02 | C3 | No | A/B |
|  | **8** | CG | N | Ksk-1 | Uninfected | 0.998884528 | 0.2386 | 0.14 | Not Detected | No | A/B |
|  | **9** | CG | N | Ksk-1 | Uninfected | 0.991378184 | 0.0126 | 0.01 | Not Detected | No | E |
| **5/6/13** | **1** | CG | N | Ws-0 | Infected | 0.999982632 | 98.2863 | 97.05 | C1 | No | F |
|  | **2** | CG | N | Ws-0 | Infected | 0.999860508 | 153.1004 | 153.06 | C1 | No | F |
|  | **3** | CG | N | Col-0 | Infected | NA | 52.0952 |  | C1 | No | Not Sequenced |
|  | **4** | CG | N | Col-0 | Infected | 0.999387748 | 157.7821 | 157.44 | C1 | No | F |
|  | **5** | CG | N | Ksk-1 | Uninfected | NA | 0.0024 |  | Not Detected | No | Not Sequenced |
|  | **6** | CG | N | Ksk-1 | Uninfected | NA | 0.2650 |  | C1 | No | Not Sequenced |
|  | **7** | CG | N | Ws-0 | Infected | 0.945382838 | 55.8369 | 39.44 | C2 | No | F |
|  | **8** | CG | N | Col-0 | Infected | 0.999642033 | 35.9873 | 35.91 | C2 | No | F |
|  | **9** | CG | N | Ksk-1 | Uninfected | NA | 0.0076 |  | Not Detected | No | Not Sequenced |

^1^ Pooled plants = Y indicates that collected leaves did not derive from a single plant, which otherwise was the case

^2^ *Arabidopsis* strains/accessions in each Tübingen site are determined using microsattelite primers by numbering distinguishible genotypes in each site

^3^ Observed *Albugo* = Infected indicates that all leaves in the collected pool had some visible white rust.

^4^ Measured *Albugo* (relative-scaled) is the measured endophytic relative abundance of *Albugo* sp. in samples (log-transformed abundances from ITS1 and ITS2 datasets scaled from 0-1 and averaged)

^5^ Measured Oomycete (Absolute) is the qPCR quantified abundance of oomycete 5.8S rRNA gene normalized to the qPCR quantified *A. thaliana* EF1-alpha gene. For comparison between samples, the *A. laibachii* Nc14-infected *A. thaliana* Col-0 sample was used as a standard.

^6^ Measured Albugo (Absolute) Is the measured oomycete value multiplied by the relative abundance of endophytic *Albugo* sp. in the genus-level grouped OTUs (the average of the relative abundances measured for each sample based on the ITS1 and ITS2 regions).

^7^ *A. laibachii* strains in each site were determined using microsattelite primers by numbering distinguishible genotypes

^6^ Presence of *A. candida* as confirmed by amplification with A. candida specific PCR primers

^8^ Sequencing run denotes which of 7 MiSeq sequencing run the samples were included on. Not sequenced samples were not included in amplicon analyses

^9^ This plant was collected directly next to the listed *Capsella* sp., and presumably this is detection of surface spores since we were unable to isolate this *Albugo candida* on *A. thaliana in the lab*
