# Supplementary material for: Microbial Hub Taxa Link Host and Abiotic Factors to Plant Microbiome Variation
Source: PLoS Biol. 2016 Jan 20;14(1):e1002352. doi: 10.1371/journal.pbio.1002352 (PMC4720289; doi:10.1371/journal.pbio.1002352)
Supplement: S3 Table — Enrichment at a location is based a significantly higher relative abundance there compared to any other location (Tukey’s HSD p < 0.01). (DOCX) [file pbio.1002352.s030.docx]

**S3 Table**

| **Compartment** | **Organism** | **Location** |
| --- | --- | --- |
| Epiphytic | k__Bacteria;p__[Thermi];c__Deinococci;o__Deinococcales;f__Deinococcaceae;g__Deinococcus | ERG |
| Epiphytic | k__Bacteria;p__[Thermi];c__Deinococci;o__Deinococcales;f__Trueperaceae;g__Truepera | ERG |
| Epiphytic | k__Bacteria;p__Actinobacteria;c__Actinobacteria;o__Actinomycetales;f__Nocardiaceae;g__Rhodococcus | ERG |
| Epiphytic | k__Bacteria;p__Cyanobacteria;c__Oscillatoriophycideae;o__Chroococcales;f__Xenococcaceae;g__ | ERG |
| Epiphytic | k__Bacteria;p__Proteobacteria;c__Gammaproteobacteria;o__Pseudomonadales;f__Pseudomonadaceae;Other | ERG |
| Epiphytic | k__Bacteria;p__Proteobacteria;c__Gammaproteobacteria;o__Pseudomonadales;Other;Other | ERG |
| Epiphytic | k__Fungi;p__Ascomycota;c__Dothideomycetes;o__Capnodiales;f__Incertae sedis;g__Capnobotryella | ERG |
| Epiphytic | k__Fungi;p__Ascomycota;c__Lecanoromycetes;o__Lecanorales;f__Incertae sedis;g__Leprocaulon | ERG |
| Epiphytic | k__Fungi;p__Ascomycota;c__Lecanoromycetes;o__Lecanorales;f__Incertae sedis;Other | ERG |
| Epiphytic | k__Fungi;p__Basidiomycota;c__Tremellomycetes;o__Tremellales;Other;Other | ERG |
| Epiphytic | k__Bacteria;p__Actinobacteria;c__Actinobacteria;o__Actinomycetales;f__Micromonosporaceae;Other | EY |
| Epiphytic | k__Bacteria;p__Proteobacteria;c__Alphaproteobacteria;o__Rhizobiales;f__Methylocystaceae;g__Methylopila | EY |
| Epiphytic | k__Fungi;p__Ascomycota;c__Eurotiomycetes;o__Chaetothyriales;f__Chaetothyriaceae;g__Cyphellophora | EY |
| Epiphytic | k__Fungi;p__Ascomycota;c__Incertae sedis;o__Incertae sedis;f__Incertae sedis;g__Chaetosphaeronema | EY |
| Epiphytic | k__Fungi;p__Basidiomycota;c__Microbotryomycetes;o__Leucosporidiales;f__Leucosporidiaceae;g__Mastigobasidium | EY |
| Endophytic | k__Bacteria;p__Proteobacteria;c__Gammaproteobacteria;o__Alteromonadales;f__[Chromatiaceae];g__ | JUG |
| Endophytic | k__Bacteria;p__Proteobacteria;c__Gammaproteobacteria;o__Alteromonadales;f__[Chromatiaceae];g__Rheinheimera | JUG |
| Epiphytic | k__Bacteria;p__Actinobacteria;c__Actinobacteria;o__Actinomycetales;f__Micromonosporaceae;Other | JUG |
| Epiphytic | k__Bacteria;p__Actinobacteria;c__Actinobacteria;o__Actinomycetales;f__Nocardiaceae;g__Rhodococcus | JUG |
| Epiphytic | k__Bacteria;p__Proteobacteria;c__Gammaproteobacteria;o__Alteromonadales;f__[Chromatiaceae];g__Rheinheimera | JUG |
| Epiphytic | k__Fungi;p__Ascomycota;c__Dothideomycetes;o__Botryosphaeriales;f__unidentified;g__unidentified | JUG |
| Epiphytic | k__Fungi;p__Ascomycota;c__Dothideomycetes;o__Pleosporales;f__Leptosphaeriaceae;g__Neosetophoma | JUG |
| Epiphytic | k__Fungi;p__Ascomycota;c__Dothideomycetes;o__Pleosporales;f__Lophiostomataceae;g__Lophiostoma | JUG |
| Epiphytic | k__Fungi;p__Ascomycota;c__Dothideomycetes;o__Pleosporales;f__Pleomassariaceae;Other | JUG |
| **Compartment** | **Organism** | **Location** |
| Epiphytic | k__Fungi;p__Ascomycota;c__Eurotiomycetes;o__Chaetothyriales;f__Chaetothyriaceae;g__Cyphellophora | JUG |
| Epiphytic | k__Fungi;p__Ascomycota;c__Eurotiomycetes;o__Chaetothyriales;f__Herpotrichiellaceae;g__Rhinocladiella | JUG |
| Epiphytic | k__Fungi;p__Ascomycota;c__Eurotiomycetes;o__Chaetothyriales;f__Incertae sedis;g__Coniosporium | JUG |
| Epiphytic | k__Fungi;p__Ascomycota;c__Lecanoromycetes;o__Lecanorales;f__Ramalinaceae;g__Bacidina | JUG |
| Epiphytic | k__Fungi;p__Ascomycota;c__Leotiomycetes;o__Helotiales;f__Incertae sedis;g__Rhynchosporium | JUG |
| Epiphytic | k__Fungi;p__Ascomycota;c__Leotiomycetes;o__Leotiales;f__Leotiaceae;g__Alatospora | JUG |
| Epiphytic | k__Fungi;p__Ascomycota;c__Sordariomycetes;o__Hypocreales;f__Clavicipitaceae;g__Claviceps | JUG |
| Epiphytic | k__Fungi;p__Basidiomycota;c__Agaricomycetes;o__Atheliales;f__Atheliaceae;g__Athelia | JUG |
| Endophytic | k__Bacteria;p__Actinobacteria;c__Acidimicrobiia;o__Acidimicrobiales;Other;Other | PFN |
| Endophytic | k__Bacteria;p__Actinobacteria;c__Thermoleophilia;o__Gaiellales;f__Gaiellaceae;g__ | PFN |
| Endophytic | k__Bacteria;p__Chloroflexi;c__Ellin6529;o__;f__;g__ | PFN |
| Endophytic | k__Bacteria;p__Firmicutes;c__Bacilli;o__Bacillales;f__Alicyclobacillaceae;g__Alicyclobacillus | PFN |
| Endophytic | k__Bacteria;p__Firmicutes;c__Bacilli;o__Bacillales;f__Bacillaceae;g__Bacillus | PFN |
| Endophytic | k__Bacteria;p__Firmicutes;c__Bacilli;o__Bacillales;f__Bacillaceae;g__Geobacillus | PFN |
| Endophytic | k__Bacteria;p__Firmicutes;c__Bacilli;o__Bacillales;f__Bacillaceae;Other | PFN |
| Endophytic | k__Bacteria;p__Firmicutes;c__Bacilli;o__Bacillales;f__Paenibacillaceae;g__Brevibacillus | PFN |
| Endophytic | k__Bacteria;p__Firmicutes;c__Bacilli;o__Bacillales;f__Paenibacillaceae;g__Paenibacillus | PFN |
| Endophytic | k__Bacteria;p__Firmicutes;c__Bacilli;o__Bacillales;f__Planococcaceae;Other | PFN |
| Endophytic | k__Bacteria;p__Firmicutes;c__Bacilli;o__Bacillales;f__Thermoactinomycetaceae;g__Planifilum | PFN |
| Endophytic | k__Bacteria;p__Firmicutes;c__Bacilli;o__Bacillales;f__Thermoactinomycetaceae;g__Thermoactinomyces | PFN |
| Endophytic | k__Bacteria;p__Firmicutes;c__Bacilli;o__Bacillales;f__Thermoactinomycetaceae;Other | PFN |
| Endophytic | k__Bacteria;p__Firmicutes;c__Bacilli;Other;Other;Other | PFN |
| Endophytic | k__Bacteria;p__Firmicutes;c__Clostridia;o__Clostridiales;f__Caldicoprobacteraceae;g__Caldicoprobacter | PFN |
| Endophytic | k__Bacteria;p__Firmicutes;c__Clostridia;o__Clostridiales;f__Clostridiaceae;Other | PFN |
| Endophytic | k__Bacteria;p__Firmicutes;c__Clostridia;o__Clostridiales;f__Peptostreptococcaceae;Other | PFN |
| Endophytic | k__Bacteria;p__Gemmatimonadetes;c__Gemmatimonadetes;o__Gemmatimonadales;f__A1-B1;g__ | PFN |
| **Compartment** | **Organism** | **Location** |
| Endophytic | k__Bacteria;p__Gemmatimonadetes;c__Gemmatimonadetes;Other;Other;Other | PFN |
| Endophytic | k__Bacteria;p__Proteobacteria;c__Alphaproteobacteria;o__Rhizobiales;f__Hyphomicrobiaceae;g__ | PFN |
| Endophytic | k__Bacteria;p__Proteobacteria;c__Alphaproteobacteria;o__Rhizobiales;f__Hyphomicrobiaceae;g__Rhodoplanes | PFN |
| Endophytic | k__Bacteria;p__Proteobacteria;c__Alphaproteobacteria;o__Rhodospirillales;f__Rhodospirillaceae;g__ | PFN |
| Endophytic | k__Bacteria;p__Proteobacteria;c__Deltaproteobacteria;o__Myxococcales;f__Haliangiaceae;g__ | PFN |
| Endophytic | k__Bacteria;p__Proteobacteria;c__Deltaproteobacteria;o__Myxococcales;f__Polyangiaceae;g__Aetherobacter | PFN |
| Endophytic | k__Bacteria;p__Verrucomicrobia;c__[Spartobacteria];o__[Chthoniobacterales];f__[Chthoniobacteraceae];g__DA101 | PFN |
| Endophytic | k__Fungi;p__Ascomycota;c__Dothideomycetes;o__Capnodiales;f__Teratosphaeriaceae;g__Devriesia | PFN |
| Endophytic | k__Fungi;p__Ascomycota;c__Dothideomycetes;o__Pleosporales;f__Incertae sedis;g__Massariosphaeria | PFN |
| Endophytic | k__Fungi;p__Ascomycota;c__Dothideomycetes;o__Pleosporales;f__Pleosporaceae;g__Dendryphion | PFN |
| Endophytic | k__Fungi;p__Ascomycota;c__Incertae sedis;o__Incertae sedis;f__Incertae sedis;g__Ochroconis | PFN |
| Endophytic | k__Fungi;p__Basidiomycota;c__Exobasidiomycetes;o__Entylomatales;f__Entylomataceae;g__Entyloma | PFN |
| Epiphytic | k__Bacteria;p__Acidobacteria;c__Acidobacteriia;o__Acidobacteriales;f__Koribacteraceae;g__ | PFN |
| Epiphytic | k__Bacteria;p__Acidobacteria;c__DA052;o__Ellin6513;f__;g__ | PFN |
| Epiphytic | k__Bacteria;p__Acidobacteria;c__iii1-8;o__32-20;f__;g__ | PFN |
| Epiphytic | k__Bacteria;p__Actinobacteria;c__Actinobacteria;o__Actinomycetales;f__Nocardioidaceae;g__Kribbella | PFN |
| Epiphytic | k__Bacteria;p__Actinobacteria;c__Actinobacteria;o__Actinomycetales;f__Streptosporangiaceae;g__Streptosporangium | PFN |
| Epiphytic | k__Bacteria;p__Chloroflexi;c__TK10;o__B07_WMSP1;f__FFCH4570;g__ | PFN |
| Epiphytic | k__Bacteria;p__Cyanobacteria;c__Synechococcophycideae;o__Pseudanabaenales;f__Pseudanabaenaceae;g__Pseudanabaena | PFN |
| Epiphytic | k__Bacteria;p__Firmicutes;c__Bacilli;o__Bacillales;f__Alicyclobacillaceae;g__Alicyclobacillus | PFN |
| Epiphytic | k__Bacteria;p__Firmicutes;c__Bacilli;o__Bacillales;f__Paenibacillaceae;g__Cohnella | PFN |
| Epiphytic | k__Bacteria;p__Firmicutes;c__Bacilli;o__Bacillales;f__Thermoactinomycetaceae;g__Thermoactinomyces | PFN |
| Epiphytic | k__Bacteria;p__Gemmatimonadetes;c__Gemmatimonadetes;o__Ellin5290;f__;g__ | PFN |
| Epiphytic | k__Bacteria;p__Gemmatimonadetes;c__Gemmatimonadetes;o__Gemmatimonadales;f__Gemmatimonadaceae;g__Gemmatimonas | PFN |
| Epiphytic | k__Bacteria;p__Proteobacteria;c__Deltaproteobacteria;o__Myxococcales;f__Myxococcaceae;g__Anaeromyxobacter | PFN |
| Epiphytic | k__Bacteria;p__Proteobacteria;c__Deltaproteobacteria;o__Spirobacillales;f__;g__ | PFN |
| **Compartment** | **Organism** | **Location** |
| Epiphytic | k__Bacteria;p__Proteobacteria;c__Gammaproteobacteria;o__HTCC2188;f__HTCC2089;g__ | PFN |
| Epiphytic | k__Bacteria;p__Verrucomicrobia;c__[Spartobacteria];o__[Chthoniobacterales];f__[Chthoniobacteraceae];g__DA101 | PFN |
| Epiphytic | k__Fungi;p__Ascomycota;c__Dothideomycetes;o__Pleosporales;f__Incertae sedis;g__Letendraea | PFN |
| Epiphytic | k__Fungi;p__Ascomycota;c__Dothideomycetes;o__Pleosporales;f__Phaeosphaeriaceae;g__Phaeosphaeriopsis | PFN |
| Epiphytic | k__Fungi;p__Ascomycota;c__Dothideomycetes;o__Pleosporales;f__Pleosporaceae;g__Pyrenophora | PFN |
| Epiphytic | k__Fungi;p__Ascomycota;c__Eurotiomycetes;o__Chaetothyriales;f__Chaetothyriaceae;g__Cyphellophora | PFN |
| Epiphytic | k__Fungi;p__Ascomycota;c__Eurotiomycetes;o__Chaetothyriales;f__Herpotrichiellaceae;g__Exophiala | PFN |
| Epiphytic | k__Fungi;p__Ascomycota;c__Sordariomycetes;o__Chaetosphaeriales;f__Chaetosphaeriaceae;g__Chloridium | PFN |
| Epiphytic | k__Fungi;p__Ascomycota;c__Sordariomycetes;o__Hypocreales;f__Incertae sedis;g__Cephalosporium | PFN |
| Epiphytic | k__Fungi;p__Ascomycota;c__Sordariomycetes;o__Hypocreales;f__Incertae sedis;Other | PFN |
| Epiphytic | k__Fungi;p__Ascomycota;c__Sordariomycetes;o__Incertae sedis;f__Plectosphaerellaceae;g__Verticillium | PFN |
| Epiphytic | k__Fungi;p__Ascomycota;c__Sordariomycetes;o__Sordariales;f__Chaetomiaceae;g__Chaetomium | PFN |
| Epiphytic | k__Fungi;p__Ascomycota;c__Sordariomycetes;o__Xylariales;f__Incertae sedis;g__Microdochium | PFN |
| Epiphytic | k__Fungi;p__Basidiomycota;c__Exobasidiomycetes;o__Entylomatales;f__Entylomataceae;g__Entyloma | PFN |
| Epiphytic | k__Fungi;p__Basidiomycota;c__Microbotryomycetes;o__Leucosporidiales;f__Leucosporidiaceae;g__Leucosporidium | PFN |
| Epiphytic | k__Fungi;p__Basidiomycota;c__Microbotryomycetes;o__Leucosporidiales;f__Leucosporidiaceae;g__Mastigobasidium | PFN |
| Epiphytic | k__Fungi;p__Zygomycota;c__Incertae sedis;o__Mortierellales;f__Mortierellaceae;g__Mortierella | PFN |
| Endophytic | k__Bacteria;p__Actinobacteria;c__Actinobacteria;o__Actinomycetales;f__Microbacteriaceae;g__Frigoribacterium | WH |
| Epiphytic | k__Bacteria;p__[Thermi];c__Deinococci;o__Deinococcales;f__Deinococcaceae;g__Deinococcus | WH |
| Epiphytic | k__Bacteria;p__Actinobacteria;c__Actinobacteria;o__Actinomycetales;f__Micromonosporaceae;Other | WH |
| Epiphytic | k__Bacteria;p__Gemmatimonadetes;c__Gemmatimonadetes;o__Gemmatimonadales;f__Gemmatimonadaceae;g__Gemmatimonas | WH |
| Epiphytic | k__Bacteria;p__Proteobacteria;c__Betaproteobacteria;o__Burkholderiales;f__Comamonadaceae;g__Variovorax | WH |
| Epiphytic | k__Fungi;p__Ascomycota;c__Dothideomycetes;o__Capnodiales;f__Incertae sedis;g__Capnobotryella | WH |
