# Supplementary material for: Microbial Hub Taxa Link Host and Abiotic Factors to Plant Microbiome Variation
Source: PLoS Biol. 2016 Jan 20;14(1):e1002352. doi: 10.1371/journal.pbio.1002352 (PMC4720289; doi:10.1371/journal.pbio.1002352)
Supplement: S4 Table — Enrichment in fall or spring is based on a significantly higher relative abundance in that season compared to the other season (Tukey’s HSD p < 0.01). (DOCX) [file pbio.1002352.s031.docx]

**S4 Table**

| **Compartment** | **Organism** | **Season** |
| --- | --- | --- |
| Endophytic | k__Bacteria;p__Bacteroidetes;c__Flavobacteriia;o__Flavobacteriales;f__[Weeksellaceae];Other | Fall |
| Endophytic | k__Bacteria;p__Bacteroidetes;c__Flavobacteriia;o__Flavobacteriales;f__Flavobacteriaceae;g__Flavobacterium | Fall |
| Endophytic | k__Bacteria;p__Bacteroidetes;c__Flavobacteriia;o__Flavobacteriales;f__Flavobacteriaceae;Other | Fall |
| Endophytic | k__Bacteria;p__Cyanobacteria;c__Oscillatoriophycideae;o__Oscillatoriales;f__Phormidiaceae;g__Phormidium | Fall |
| Endophytic | k__Bacteria;p__Planctomycetes;c__Planctomycetia;o__Gemmatales;f__Isosphaeraceae;g__ | Fall |
| Endophytic | k__Bacteria;p__Proteobacteria;c__Alphaproteobacteria;o__Caulobacterales;f__Caulobacteraceae;g__Caulobacter | Fall |
| Endophytic | k__Bacteria;p__Proteobacteria;c__Alphaproteobacteria;o__Rhizobiales;f__Aurantimonadaceae;g__Aurantimonas | Fall |
| Endophytic | k__Bacteria;p__Proteobacteria;c__Alphaproteobacteria;o__Rhizobiales;f__Aurantimonadaceae;Other | Fall |
| Endophytic | k__Bacteria;p__Proteobacteria;c__Alphaproteobacteria;o__Rhizobiales;f__Methylobacteriaceae;g__Methylobacterium | Fall |
| Endophytic | k__Bacteria;p__Proteobacteria;c__Alphaproteobacteria;o__Rhizobiales;Other;Other | Fall |
| Endophytic | k__Bacteria;p__Proteobacteria;c__Alphaproteobacteria;o__Rhodobacterales;f__Rhodobacteraceae;g__Rhodobacter | Fall |
| Endophytic | k__Bacteria;p__Proteobacteria;c__Alphaproteobacteria;o__Sphingomonadales;f__Erythrobacteraceae;g__Erythrobacter | Fall |
| Endophytic | k__Bacteria;p__Proteobacteria;c__Alphaproteobacteria;o__Sphingomonadales;Other;Other | Fall |
| Endophytic | k__Bacteria;p__Proteobacteria;c__Betaproteobacteria;o__Burkholderiales;f__Comamonadaceae;g__Acidovorax | Fall |
| Endophytic | k__Bacteria;p__Proteobacteria;c__Betaproteobacteria;o__Burkholderiales;f__Comamonadaceae;g__Methylibium | Fall |
| Endophytic | k__Bacteria;p__Proteobacteria;c__Betaproteobacteria;o__Burkholderiales;f__Comamonadaceae;g__Pelomonas | Fall |
| Endophytic | k__Bacteria;p__Proteobacteria;c__Betaproteobacteria;o__Burkholderiales;f__Comamonadaceae;g__Polaromonas | Fall |
| Endophytic | k__Bacteria;p__Proteobacteria;c__Betaproteobacteria;o__Burkholderiales;f__Oxalobacteraceae;g__Herbaspirillum | Fall |
| Endophytic | k__Bacteria;p__Proteobacteria;c__Betaproteobacteria;o__Burkholderiales;f__Oxalobacteraceae;g__Janthinobacterium | Fall |
| Endophytic | k__Bacteria;p__Proteobacteria;c__Betaproteobacteria;o__Burkholderiales;f__Oxalobacteraceae;g__Massilia | Fall |
| Endophytic | k__Bacteria;p__Proteobacteria;c__Betaproteobacteria;o__Methylophilales;f__Methylophilaceae;g__Methylotenera | Fall |
| Endophytic | k__Bacteria;p__Proteobacteria;c__Betaproteobacteria;o__Methylophilales;f__Methylophilaceae;Other | Fall |
| Endophytic | k__Bacteria;p__Proteobacteria;c__Betaproteobacteria;Other;Other;Other | Fall |
| Endophytic | k__Bacteria;p__Proteobacteria;c__Gammaproteobacteria;o__Alteromonadales;f__Alteromonadaceae;g__Cellvibrio | Fall |
| **Compartment** | **Organism** | **Season** |
| Endophytic | k__Bacteria;p__Proteobacteria;c__Gammaproteobacteria;o__Xanthomonadales;f__Xanthomonadaceae;g__Arenimonas | Fall |
| Endophytic | k__Bacteria;p__Proteobacteria;Other;Other;Other;Other | Fall |
| Endophytic | k__Fungi;p__Ascomycota;c__Sordariomycetes;o__Hypocreales;f__Incertae sedis;g__Acremonium | Fall |
| Endophytic | k__Fungi;p__Basidiomycota;c__Agaricomycetes;o__Agaricales;f__Agaricaceae;g__Lycoperdon | Fall |
| Endophytic | k__Fungi;p__Basidiomycota;c__Agaricomycetes;o__Agaricales;f__Psathyrellaceae;g__Coprinellus | Fall |
| Endophytic | k__Fungi;p__Basidiomycota;c__Agaricomycetes;o__Agaricales;f__Strophariaceae;g__Hypholoma | Fall |
| Epiphytic | k__Bacteria;p__[Thermi];c__Deinococci;o__Deinococcales;f__Deinococcaceae;g__R18-435 | Fall |
| Epiphytic | k__Bacteria;p__Acidobacteria;c__Acidobacteria-6;Other;Other;Other | Fall |
| Epiphytic | k__Bacteria;p__Acidobacteria;c__Solibacteres;o__Solibacterales;Other;Other | Fall |
| Epiphytic | k__Bacteria;p__Actinobacteria;c__Acidimicrobiia;o__Acidimicrobiales;f__C111;g__ | Fall |
| Epiphytic | k__Bacteria;p__Actinobacteria;c__Acidimicrobiia;o__Acidimicrobiales;f__EB1017;g__ | Fall |
| Epiphytic | k__Bacteria;p__Actinobacteria;c__Acidimicrobiia;o__Acidimicrobiales;f__Iamiaceae;Other | Fall |
| Epiphytic | k__Bacteria;p__Actinobacteria;c__Acidimicrobiia;o__Acidimicrobiales;Other;Other | Fall |
| Epiphytic | k__Bacteria;p__Actinobacteria;c__Actinobacteria;o__Actinomycetales;f__Dermatophilaceae;g__Piscicoccus | Fall |
| Epiphytic | k__Bacteria;p__Actinobacteria;c__Actinobacteria;o__Actinomycetales;f__Frankiaceae;Other | Fall |
| Epiphytic | k__Bacteria;p__Actinobacteria;c__Actinobacteria;o__Actinomycetales;f__Geodermatophilaceae;g__ | Fall |
| Epiphytic | k__Bacteria;p__Actinobacteria;c__Actinobacteria;o__Actinomycetales;f__Geodermatophilaceae;g__Blastococcus | Fall |
| Epiphytic | k__Bacteria;p__Actinobacteria;c__Actinobacteria;o__Actinomycetales;f__Kineosporiaceae;g__Kineococcus | Fall |
| Epiphytic | k__Bacteria;p__Actinobacteria;c__Actinobacteria;o__Actinomycetales;f__Microbacteriaceae;g__Agromyces | Fall |
| Epiphytic | k__Bacteria;p__Actinobacteria;c__Actinobacteria;o__Actinomycetales;f__Microbacteriaceae;g__Microbacterium | Fall |
| Epiphytic | k__Bacteria;p__Actinobacteria;c__Actinobacteria;o__Actinomycetales;f__Micrococcaceae;Other | Fall |
| Epiphytic | k__Bacteria;p__Actinobacteria;c__Actinobacteria;o__Actinomycetales;f__Micromonosporaceae;g__Actinoplanes | Fall |
| Epiphytic | k__Bacteria;p__Actinobacteria;c__Actinobacteria;o__Actinomycetales;f__Micromonosporaceae;g__Couchioplanes | Fall |
| Epiphytic | k__Bacteria;p__Actinobacteria;c__Actinobacteria;o__Actinomycetales;f__Micromonosporaceae;Other | Fall |
| Epiphytic | k__Bacteria;p__Actinobacteria;c__Actinobacteria;o__Actinomycetales;f__Propionibacteriaceae;g__Microlunatus | Fall |
| Epiphytic | k__Bacteria;p__Actinobacteria;c__Actinobacteria;o__Actinomycetales;f__Pseudonocardiaceae;g__Pseudonocardia | Fall |
| **Compartment** | **Organism** | **Season** |
| Epiphytic | k__Bacteria;p__Actinobacteria;c__Thermoleophilia;o__Solirubrobacterales;f__Conexibacteraceae;g__ | Fall |
| Epiphytic | k__Bacteria;p__Actinobacteria;c__Thermoleophilia;o__Solirubrobacterales;f__Solirubrobacteraceae;Other | Fall |
| Epiphytic | k__Bacteria;p__Actinobacteria;Other;Other;Other;Other | Fall |
| Epiphytic | k__Bacteria;p__Bacteroidetes;c__Cytophagia;o__Cytophagales;f__Cytophagaceae;g__ | Fall |
| Epiphytic | k__Bacteria;p__Bacteroidetes;c__Sphingobacteriia;o__Sphingobacteriales;f__;g__ | Fall |
| Epiphytic | k__Bacteria;p__Bacteroidetes;c__Sphingobacteriia;o__Sphingobacteriales;Other;Other | Fall |
| Epiphytic | k__Bacteria;p__Bacteroidetes;Other;Other;Other;Other | Fall |
| Epiphytic | k__Bacteria;p__Chloroflexi;c__Anaerolineae;o__SBR1031;f__A4b;g__ | Fall |
| Epiphytic | k__Bacteria;p__Chloroflexi;c__Thermomicrobia;o__AKYG1722;f__;g__ | Fall |
| Epiphytic | k__Bacteria;p__Chloroflexi;c__Thermomicrobia;o__JG30-KF-CM45;f__;g__ | Fall |
| Epiphytic | k__Bacteria;p__Elusimicrobia;c__Elusimicrobia;o__IIb;f__;g__ | Fall |
| Epiphytic | k__Bacteria;p__Fibrobacteres;c__Fibrobacteria;o__258ds10;f__;g__ | Fall |
| Epiphytic | k__Bacteria;p__Firmicutes;c__Bacilli;o__Bacillales;f__Planococcaceae;g__Sporosarcina | Fall |
| Epiphytic | k__Bacteria;p__Gemmatimonadetes;c__Gemmatimonadetes;Other;Other;Other | Fall |
| Epiphytic | k__Bacteria;p__Planctomycetes;c__vadinHA49;o__DH61;f__;g__ | Fall |
| Epiphytic | k__Bacteria;p__Proteobacteria;c__Alphaproteobacteria;o__Caulobacterales;f__Caulobacteraceae;g__Brevundimonas | Fall |
| Epiphytic | k__Bacteria;p__Proteobacteria;c__Alphaproteobacteria;o__Caulobacterales;f__Caulobacteraceae;g__Caulobacter | Fall |
| Epiphytic | k__Bacteria;p__Proteobacteria;c__Alphaproteobacteria;o__Caulobacterales;f__Caulobacteraceae;g__Phenylobacterium | Fall |
| Epiphytic | k__Bacteria;p__Proteobacteria;c__Alphaproteobacteria;o__Ellin329;f__;g__ | Fall |
| Epiphytic | k__Bacteria;p__Proteobacteria;c__Alphaproteobacteria;o__Rhizobiales;f__Aurantimonadaceae;g__Aurantimonas | Fall |
| Epiphytic | k__Bacteria;p__Proteobacteria;c__Alphaproteobacteria;o__Rhizobiales;f__Aurantimonadaceae;Other | Fall |
| Epiphytic | k__Bacteria;p__Proteobacteria;c__Alphaproteobacteria;o__Rhizobiales;f__Bradyrhizobiaceae;Other | Fall |
| Epiphytic | k__Bacteria;p__Proteobacteria;c__Alphaproteobacteria;o__Rhizobiales;f__Hyphomicrobiaceae;g__Rhodoplanes | Fall |
| Epiphytic | k__Bacteria;p__Proteobacteria;c__Alphaproteobacteria;o__Rhizobiales;f__Hyphomicrobiaceae;Other | Fall |
| Epiphytic | k__Bacteria;p__Proteobacteria;c__Alphaproteobacteria;o__Rhizobiales;f__Phyllobacteriaceae;g__Mesorhizobium | Fall |
| Epiphytic | k__Bacteria;p__Proteobacteria;c__Alphaproteobacteria;o__Rhizobiales;Other;Other | Fall |
| **Compartment** | **Organism** | **Season** |
| Epiphytic | k__Bacteria;p__Proteobacteria;c__Alphaproteobacteria;o__Rhodobacterales;f__Hyphomonadaceae;g__ | Fall |
| Epiphytic | k__Bacteria;p__Proteobacteria;c__Alphaproteobacteria;o__Rhodobacterales;f__Rhodobacteraceae;g__Paracoccus | Fall |
| Epiphytic | k__Bacteria;p__Proteobacteria;c__Alphaproteobacteria;o__Rhodobacterales;Other;Other | Fall |
| Epiphytic | k__Bacteria;p__Proteobacteria;c__Alphaproteobacteria;o__Rhodospirillales;f__;g__ | Fall |
| Epiphytic | k__Bacteria;p__Proteobacteria;c__Alphaproteobacteria;o__Rhodospirillales;f__Acetobacteraceae;g__Roseomonas | Fall |
| Epiphytic | k__Bacteria;p__Proteobacteria;c__Alphaproteobacteria;o__Rhodospirillales;f__Rhodospirillaceae;Other | Fall |
| Epiphytic | k__Bacteria;p__Proteobacteria;c__Alphaproteobacteria;o__Rhodospirillales;Other;Other | Fall |
| Epiphytic | k__Bacteria;p__Proteobacteria;c__Alphaproteobacteria;o__Sphingomonadales;f__Erythrobacteraceae;g__Erythrobacter | Fall |
| Epiphytic | k__Bacteria;p__Proteobacteria;c__Alphaproteobacteria;o__Sphingomonadales;f__Sphingomonadaceae;g__Novosphingobium | Fall |
| Epiphytic | k__Bacteria;p__Proteobacteria;c__Alphaproteobacteria;Other;Other;Other | Fall |
| Epiphytic | k__Bacteria;p__Proteobacteria;c__Betaproteobacteria;o__Burkholderiales;f__Burkholderiaceae;g__ | Fall |
| Epiphytic | k__Bacteria;p__Proteobacteria;c__Betaproteobacteria;o__Burkholderiales;f__Comamonadaceae;g__Acidovorax | Fall |
| Epiphytic | k__Bacteria;p__Proteobacteria;c__Betaproteobacteria;o__Burkholderiales;f__Comamonadaceae;g__Leptothrix | Fall |
| Epiphytic | k__Bacteria;p__Proteobacteria;c__Betaproteobacteria;o__Burkholderiales;f__Comamonadaceae;g__Methylibium | Fall |
| Epiphytic | k__Bacteria;p__Proteobacteria;c__Betaproteobacteria;o__Burkholderiales;f__Comamonadaceae;g__Pelomonas | Fall |
| Epiphytic | k__Bacteria;p__Proteobacteria;c__Betaproteobacteria;o__Burkholderiales;f__Comamonadaceae;g__Polaromonas | Fall |
| Epiphytic | k__Bacteria;p__Proteobacteria;c__Betaproteobacteria;o__Burkholderiales;f__Comamonadaceae;g__Xylophilus | Fall |
| Epiphytic | k__Bacteria;p__Proteobacteria;c__Betaproteobacteria;o__Burkholderiales;f__Oxalobacteraceae;g__Janthinobacterium | Fall |
| Epiphytic | k__Bacteria;p__Proteobacteria;c__Betaproteobacteria;o__Burkholderiales;f__Oxalobacteraceae;g__Massilia | Fall |
| Epiphytic | k__Bacteria;p__Proteobacteria;c__Betaproteobacteria;o__Burkholderiales;Other;Other | Fall |
| Epiphytic | k__Bacteria;p__Proteobacteria;c__Betaproteobacteria;o__Ellin6067;f__;g__ | Fall |
| Epiphytic | k__Bacteria;p__Proteobacteria;c__Betaproteobacteria;o__SC-I-84;f__;g__ | Fall |
| Epiphytic | k__Bacteria;p__Proteobacteria;c__Betaproteobacteria;Other;Other;Other | Fall |
| Epiphytic | k__Bacteria;p__Proteobacteria;c__Deltaproteobacteria;o__Bdellovibrionales;f__Bacteriovoracaceae;g__ | Fall |
| Epiphytic | k__Bacteria;p__Proteobacteria;c__Gammaproteobacteria;o__Alteromonadales;f__Alteromonadaceae;g__Cellvibrio | Fall |
| Epiphytic | k__Bacteria;p__Proteobacteria;c__Gammaproteobacteria;o__Alteromonadales;Other;Other | Fall |
| **Compartment** | **Organism** | **Season** |
| Epiphytic | k__Bacteria;p__Proteobacteria;c__Gammaproteobacteria;o__Xanthomonadales;f__Xanthomonadaceae;g__Arenimonas | Fall |
| Epiphytic | k__Bacteria;p__Proteobacteria;c__Gammaproteobacteria;o__Xanthomonadales;f__Xanthomonadaceae;g__Dyella | Fall |
| Epiphytic | k__Bacteria;p__Proteobacteria;c__Gammaproteobacteria;o__Xanthomonadales;f__Xanthomonadaceae;g__Fulvimonas | Fall |
| Epiphytic | k__Bacteria;p__Proteobacteria;c__Gammaproteobacteria;o__Xanthomonadales;Other;Other | Fall |
| Epiphytic | k__Bacteria;p__Proteobacteria;Other;Other;Other;Other | Fall |
| Epiphytic | k__Bacteria;p__Verrucomicrobia;c__[Pedosphaerae];o__[Pedosphaerales];Other;Other | Fall |
| Epiphytic | k__Bacteria;p__Verrucomicrobia;c__Verrucomicrobiae;o__Verrucomicrobiales;f__Verrucomicrobiaceae;g__ | Fall |
| Epiphytic | k__Bacteria;p__Verrucomicrobia;c__Verrucomicrobiae;o__Verrucomicrobiales;f__Verrucomicrobiaceae;g__Luteolibacter | Fall |
| Epiphytic | k__Fungi;p__Ascomycota;c__Dothideomycetes;o__Capnodiales;f__Davidiellaceae;g__Cladosporium | Fall |
| Epiphytic | k__Fungi;p__Ascomycota;c__Dothideomycetes;o__Capnodiales;f__Davidiellaceae;Other | Fall |
| Epiphytic | k__Fungi;p__Ascomycota;c__Dothideomycetes;o__Capnodiales;f__Mycosphaerellaceae;g__unidentified | Fall |
| Epiphytic | k__Fungi;p__Ascomycota;c__Dothideomycetes;o__Capnodiales;Other;Other | Fall |
| Epiphytic | k__Fungi;p__Ascomycota;c__Dothideomycetes;o__Dothideales;f__Dothioraceae;g__Aureobasidium | Fall |
| Epiphytic | k__Fungi;p__Ascomycota;c__Dothideomycetes;o__Pleosporales;f__Cucurbitariaceae;g__Pyrenochaetopsis | Fall |
| Epiphytic | k__Fungi;p__Ascomycota;c__Dothideomycetes;o__Pleosporales;f__Pleosporaceae;g__Alternaria | Fall |
| Epiphytic | k__Fungi;p__Ascomycota;c__Dothideomycetes;o__Pleosporales;f__unidentified;g__unidentified | Fall |
| Epiphytic | k__Fungi;p__Ascomycota;c__Eurotiomycetes;o__Eurotiales;f__Trichocomaceae;g__Aspergillus | Fall |
| Epiphytic | k__Fungi;p__Ascomycota;c__Leotiomycetes;Other;Other;Other | Fall |
| Epiphytic | k__Fungi;p__Ascomycota;c__Sordariomycetes;o__Hypocreales;f__Nectriaceae;g__Gibberella | Fall |
| Epiphytic | k__Fungi;p__Basidiomycota;c__Agaricomycetes;o__Russulales;f__Bondarzewiaceae;g__Heterobasidion | Fall |
| Endophytic | k__Bacteria;p__Actinobacteria;c__Actinobacteria;o__Actinomycetales;f__Corynebacteriaceae;g__Corynebacterium | Spring |
| Endophytic | k__Bacteria;p__Actinobacteria;c__Actinobacteria;o__Actinomycetales;f__Nocardiaceae;g__Rhodococcus | Spring |
| Endophytic | k__Bacteria;p__Firmicutes;c__Bacilli;o__Lactobacillales;f__Streptococcaceae;g__Streptococcus | Spring |
| Endophytic | k__Bacteria;p__Proteobacteria;c__Gammaproteobacteria;o__Xanthomonadales;f__Xanthomonadaceae;g__Stenotrophomonas | Spring |
| Endophytic | k__Bacteria;p__Proteobacteria;Other;Other;Other;Other | Spring |
| Endophytic | k__Fungi;p__Ascomycota;c__Dothideomycetes;o__Pleosporales;f__Pleosporaceae;g__Lewia | Spring |
| **Compartment** | **Organism** | **Season** |
| Epiphytic | k__Bacteria;p__Actinobacteria;c__Actinobacteria;o__Actinomycetales;f__Microbacteriaceae;Other | Spring |
| Epiphytic | k__Bacteria;p__Actinobacteria;c__Actinobacteria;o__Actinomycetales;f__Nocardiaceae;g__ | Spring |
| Epiphytic | k__Bacteria;p__Actinobacteria;c__Actinobacteria;o__Actinomycetales;f__Nocardioidaceae;g__Pimelobacter | Spring |
| Epiphytic | k__Bacteria;p__Bacteroidetes;c__Cytophagia;o__Cytophagales;f__Cytophagaceae;g__Hymenobacter | Spring |
| Epiphytic | k__Bacteria;p__Firmicutes;c__Bacilli;o__Lactobacillales;f__Streptococcaceae;g__Streptococcus | Spring |
| Epiphytic | k__Bacteria;p__Proteobacteria;c__Gammaproteobacteria;o__Xanthomonadales;f__Xanthomonadaceae;g__Stenotrophomonas | Spring |
| Epiphytic | k__Fungi;p__Ascomycota;c__Dothideomycetes;o__Pleosporales;f__Pleosporaceae;g__Lewia | Spring |
| Epiphytic | k__Fungi;p__Ascomycota;c__Leotiomycetes;o__Helotiales;f__Incertae sedis;g__unidentified | Spring |
| Epiphytic | k__Fungi;p__Ascomycota;c__Taphrinomycetes;o__Taphrinales;f__Protomycetaceae;g__Protomyces | Spring |
| Epiphytic | k__Fungi;p__Basidiomycota;c__Exobasidiomycetes;o__Entylomatales;f__unidentified;g__unidentified | Spring |
| Epiphytic | k__Fungi;p__Basidiomycota;c__Exobasidiomycetes;Other;Other;Other | Spring |
| Epiphytic | k__Fungi;p__Basidiomycota;c__Microbotryomycetes;o__Leucosporidiales;f__Leucosporidiaceae;g__Leucosporidiella | Spring |
| Epiphytic | k__Fungi;p__Basidiomycota;c__Microbotryomycetes;o__Leucosporidiales;f__Leucosporidiaceae;g__Mastigobasidium | Spring |
| Epiphytic | k__Fungi;p__Basidiomycota;c__Microbotryomycetes;o__Sporidiobolales;f__Incertae sedis;g__Rhodotorula | Spring |
| Epiphytic | k__Fungi;p__Basidiomycota;c__Microbotryomycetes;Other;Other;Other | Spring |
| Epiphytic | k__Fungi;p__Basidiomycota;c__Tremellomycetes;o__Cystofilobasidiales;f__Cystofilobasidiaceae;g__Cystofilobasidium | Spring |
| Epiphytic | k__Fungi;p__Basidiomycota;c__Tremellomycetes;o__Cystofilobasidiales;f__Cystofilobasidiaceae;g__Udeniomyces | Spring |
| Epiphytic | k__Fungi;p__Basidiomycota;c__Tremellomycetes;o__Tremellales;f__Incertae sedis;g__Cryptococcus | Spring |
| Epiphytic | k__Fungi;p__Basidiomycota;c__Tremellomycetes;o__Tremellales;f__Incertae sedis;g__Dioszegia | Spring |
