# Supplementary material for: Microbial Hub Taxa Link Host and Abiotic Factors to Plant Microbiome Variation
Source: PLoS Biol. 2016 Jan 20;14(1):e1002352. doi: 10.1371/journal.pbio.1002352 (PMC4720289; doi:10.1371/journal.pbio.1002352)
Supplement: S5 Table — Enrichment on an accession is based a significantly higher relative abundance on that accession compared to any other accession (Tukey’s HSD p < 0.05). (DOCX) [file pbio.1002352.s032.docx]

**S5 Table**

| **Compartment** | | **Organism** | **Accession** |
| --- | --- | --- | --- |
| Endophytic | k__Bacteria;p__Proteobacteria;Other;Other;Other;Other | | Ws-0 |
| Endophytic | k__Fungi;p__Basidiomycota;c__Microbotryomycetes;o__Leucosporidiales;f__Leucosporidiaceae;g__Leucosporidiella | | Ws-0 |
| Epiphytic | k__Bacteria;p__Actinobacteria;c__Actinobacteria;o__Actinomycetales;f__Nocardioidaceae;g__Aeromicrobium | | Ws-0 |
| Epiphytic | k__Bacteria;p__Actinobacteria;c__Actinobacteria;o__Actinomycetales;f__Propionibacteriaceae;g__Propionibacterium | | Ws-0 |
| Epiphytic | k__Bacteria;p__Proteobacteria;c__Alphaproteobacteria;o__Rhodobacterales;f__Rhodobacteraceae;g__Rubellimicrobium | | Ws-0 |
| Epiphytic | k__Bacteria;p__Proteobacteria;c__Alphaproteobacteria;o__Rhodospirillales;f__Acetobacteraceae;g__Roseomonas | | Ws-0 |
| Epiphytic | k__Bacteria;p__Proteobacteria;c__Betaproteobacteria;o__Methylophilales;f__Methylophilaceae;Other | | Ws-0 |
| Epiphytic | k__Fungi;p__Ascomycota;c__Sordariomycetes;o__Hypocreales;f__Incertae sedis;g__Acremonium | | Ws-0 |
| Endophytic | k__Bacteria;p__Proteobacteria;Other;Other;Other;Other | | Col-0 |
| Endophytic | k__Fungi;p__Basidiomycota;c__Microbotryomycetes;o__Leucosporidiales;f__Leucosporidiaceae;g__Leucosporidiella | | Col-0 |
| Epiphytic | k__Bacteria;p__Chloroflexi;c__Chloroflexi;o__[Roseiflexales];f__[Kouleothrixaceae];Other | | Col-0 |
| Epiphytic | k__Bacteria;p__Proteobacteria;c__Alphaproteobacteria;o__Rhodospirillales;f__Acetobacteraceae;g__Roseomonas | | Col-0 |
| Epiphytic | k__Fungi;p__Ascomycota;c__Dothideomycetes;o__Capnodiales;f__Davidiellaceae;g__Cladosporium | | Col-0 |
| Epiphytic | k__Fungi;p__Ascomycota;c__Dothideomycetes;o__Incertae sedis;f__Pseudeurotiaceae;g__unidentified | | Col-0 |
| Epiphytic | k__Fungi;p__Ascomycota;c__Dothideomycetes;o__Pleosporales;f__Incertae sedis;g__Ascochyta | | Col-0 |
| Epiphytic | k__Fungi;p__Ascomycota;c__Leotiomycetes;o__Helotiales;f__Incertae sedis;g__unidentified | | Col-0 |
| Endophytic | k__Bacteria;p__Actinobacteria;c__Actinobacteria;o__Actinomycetales;f__Micrococcaceae;g__Arthrobacter | | Ksk-1 |
| Endophytic | k__Bacteria;p__Proteobacteria;c__Gammaproteobacteria;o__Enterobacteriales;f__Enterobacteriaceae;Other | | Ksk-1 |
| Epiphytic | k__Bacteria;p__Actinobacteria;c__Actinobacteria;o__Actinomycetales;f__Propionibacteriaceae;g__Propionibacterium | | Ksk-1 |
| Epiphytic | k__Bacteria;p__Firmicutes;c__Bacilli;o__Bacillales;f__Thermoactinomycetaceae;g__Planifilum | | Ksk-1 |
| Epiphytic | k__Bacteria;p__Proteobacteria;c__Alphaproteobacteria;o__Rhodobacterales;f__Rhodobacteraceae;g__Rubellimicrobium | | Ksk-1 |
| Epiphytic | k__Bacteria;p__Proteobacteria;c__Betaproteobacteria;o__Methylophilales;f__Methylophilaceae;Other | | Ksk-1 |
| Epiphytic | k__Fungi;p__Ascomycota;c__Dothideomycetes;o__Pleosporales;f__Pleosporaceae;g__Dendryphion | | Ksk-1 |
