# Supplementary material for: Microbial Hub Taxa Link Host and Abiotic Factors to Plant Microbiome Variation
Source: PLoS Biol. 2016 Jan 20;14(1):e1002352. doi: 10.1371/journal.pbio.1002352 (PMC4720289; doi:10.1371/journal.pbio.1002352)
Supplement: S6 Table — (DOCX) [file pbio.1002352.s033.docx]

**S6 Table**

|  | **Possible Correlations** | | | | |  | | |  | | |  | | |  | | |  | | |  | |
| --- | --- | --- | --- | --- | --- | --- | --- | --- | --- | --- | --- | --- | --- | --- | --- | --- | --- | --- | --- | --- | --- | --- |
|  |  |  | | | **Bac-Epi** | | **Bac-Endo** | | | **Fungi-Epi** | | | **Fungi-Endo** | | | **Oom-Epi** | | | **Oom-Endo** | | |  |
|  |  | **No. Genera^1^** | | | 234 | | 72 | | | 89 | | | 23 | | | 3 | | | 5 | | |  |
| **Bacteria** | **Epiphyte** | 234 | | | 27261 | |  | | |  | | |  | | |  | | |  | | |  |
|  | **Endophyte** | 72 | | | 16848 | | 2556 | | |  | | |  | | |  | | |  | | |  |
| **Fungi** | **Epiphyte** | 89 | | | 20826 | | 6408 | | | 3916 | | |  | | |  | | |  | | |  |
|  | **Endophyte** | 23 | | | 5382 | | 1656 | | | 2047 | | | 253 | | |  | | |  | | |  |
| **Oomycete** | **Epiphyte** | 3 | | | 702 | | 216 | | | 267 | | | 69 | | | 3 | | |  | | |  |
|  | **Endophyte** | 5 | | | 1170 | | 360 | | | 445 | | | 115 | | | 15 | | | 10 | | |  |
|  |  |  | | |  | |  | | |  | | |  | | |  | | |  | | |  |
|  | **Observed Correlations^2^** | | | | |  | | |  | | |  | | |  | | |  | | |  | |
|  |  | |  | **Bac-Epi** | | | | **Bac-Endo** | | | **Fungi-Epi** | | | **Fungi-Endo** | | | **Oom-Epi** | | | **Oom-Endo** | |  |
| **Bacteria** | **Epiphyte** | |  | 533 | | | |  | | |  | | |  | | |  | | |  | |  |
|  | **Endophyte** | |  | 65 | | | | 11 | | |  | | |  | | |  | | |  | |  |
| **Fungi** | **Epiphyte** | |  | 70 | | | | 6 | | | 16 | | |  | | |  | | |  | |  |
|  | **Endophyte** | |  | 8 | | | | 0 | | | 1 | | | 2 | | |  | | |  | |  |
| **Oomycete** | **Epiphyte** | |  | 4 | | | | 0 | | | 1 | | | 0 | | | 1 | | |  | |  |
|  | **Endophyte** | |  | 36 | | | | 8 | | | 8 | | | 0 | | | 0 | | | 1 | |  |

^1^ Number of genera refers to the number that passed abundance cutoffs before checking for correlations

^2^ Observed correlations are those supported by all collected samples (wild and common garden experiment) as well as the subset of wild samples only and are additionally filtered for only the strength of correlation (see supporting materials and methods in S1 Text)
