# Supplementary material for: Microbial Hub Taxa Link Host and Abiotic Factors to Plant Microbiome Variation
Source: PLoS Biol. 2016 Jan 20;14(1):e1002352. doi: 10.1371/journal.pbio.1002352 (PMC4720289; doi:10.1371/journal.pbio.1002352)
Supplement: S7 Table — (DOCX) [file pbio.1002352.s034.docx]

**S7 Table^1^**

| **Taxonomic Grouping Tested:** | | | |
| --- | --- | --- | --- |
| **Order-Level** | **Family-Level** | **Genus-Level** | **Species-Level** |
| **Fungi** | | | |
| Agaricales |  |  |  |
|  |  |  | Lewia infectoria |
|  |  | Udeniomyces |  |
| Tremellales |  | Dioszegia | Dioszegia buhagiarii |
|  | Bondarzewiaceae |  |  |
| **Oomycetes** | | | |
| Albuginales | Albuginaceae | Albugo | Albugo laibachii |
| **Bacteria** | | | |
|  |  | Caulobacter | Caulobacter; Other |
| Burkholderiales |  | Comamonadaceae; genus: Other | Comamonadaceae; species: Other |
| Deinococcales |  |  |  |
| DH61 | DH61; family: Other |  | DH61; species: Other |
| Ellin329 | Ellin329; family: Other |  |  |
| Gemmatales |  |  |  |
|  | Hyphomonadaceae | Hyphomonadaceae; genus: Other |  |

^1^ For each level of taxonomic grouping, we identified taxa as hubs if they were discovered to have significantly higher betweenness centrality, closeness centrality and degree than other taxa using any of 5 significance cutoffs for correlations.
