# Supplementary material for: Microbial Hub Taxa Link Host and Abiotic Factors to Plant Microbiome Variation
Source: PLoS Biol. 2016 Jan 20;14(1):e1002352. doi: 10.1371/journal.pbio.1002352 (PMC4720289; doi:10.1371/journal.pbio.1002352)
Supplement: S8 Table — (DOCX) [file pbio.1002352.s035.docx]

**S8 Table**

| **Strain** | **Closest Blast Match^1^**  **(identity in %)** | **Isolated from** | **Isolated by** |
| --- | --- | --- | --- |
| *Dioszegia* sp. | *Diozegia hungarica* (100) | Arabidopsis leaves | This study |
| Agromyces sp. | *Agromyces sp.* N13_011-11 (99) | Rhizosphere | Bai et al. (2015) |
| Flavobacterium sp. | *Flavobacterium resistans* (99) | Arabidopsis leaves | This study |
| Rhodococcus sp. | *Rhodococcus fascians* (99) | Nc14 spore solution | This study |
| *Pseudomonas sp.* | *Pseudomonas helmanticensis* (99) | Arabidopsis leaves | This study |
| *Caulobacter sp.* | *Caulobacter sp*. CR 7-03 (99) | Rhizosphere | Bai et al. (2015) |
| Janthinobacterium sp. | *Janthinobacterium sp.* CC13A2 (99) | Rhizosphere | Bai et al. (2015) |

^1^Closest Blast Match refers to the top match in a Blast search with default settings of the organism 16S sequence (identified by amplification of pure-culture extracted genomic DNA with 8F/1492R primers and sanger sequencing) against the NCBI 16S rRNA gene database.
