# Supplementary material for: Microbial Hub Taxa Link Host and Abiotic Factors to Plant Microbiome Variation
Source: PLoS Biol. 2016 Jan 20;14(1):e1002352. doi: 10.1371/journal.pbio.1002352 (PMC4720289; doi:10.1371/journal.pbio.1002352)
Supplement: S1 Text — (DOCX) [file pbio.1002352.s036.docx]

**Supporting Materials and Methods**

**For: Microbial hub taxa link host and abiotic factors to plant microbiome variation**

Matthew T. Agler, Jonas Ruhe, Samuel Kroll, Constanze Morhenn, Sang-Tae Kim, Detlef Weigel, Eric M. Kemen

***Infection of* A. thaliana *with lab strains of* Albugo sp.**

*A. candida* Nc2 and *A. laibachii* Nc14 had previously been kept alive *via* weekly re-inoculation on *A. thaliana* Ws-0 or Col-TH0, respectively, for > 1 year. Two weeks before the experiment, each species was infected on Ws-0, from which solutions of 15 x 10^4^ conidiospores per mL were prepared. A second *Albugo* spore-free solution was prepared from this by sonicating the spore solution for 5 seconds and then filtering through a 6 μm filter to retain microbes that were smaller than spores. Alternatively, for chemical inhibition of *Albugo*, the spore solution was treated with 10 μg/mL benalaxyl and 50 μg/mL of metalaxyl. Next, three 5-week old plants (2 weeks in seedling stage followed by pricking into half of a 6-pack and 3 more weeks of growth at short day conditions) of each *A. thaliana* accession (Ws-0, Col-0, or Ksk-1) was inoculated separately with spore solutions or the *Albugo* spore-free control solution of microbes. In the experiment with chemical inhibition of *Albugo*, we included a set of plants treated with only water or water with metalaxyl/benalaxyl. Within each replicate experiment, all treatments were kept under identical conditions in the same growth chamber. Sprayed plants were placed overnight in a dark cold room (4^o^C), then were kept one day in a clear plastic bag in incubation chambers set on a 12-hour light (20^o^C) / 12-hour dark (16^o^C) cycle. After removing from the clear bag, the three accessions for each treatment (9 plants) were covered with autoclaved Mira cloth to limit the spread of spores and microbes by air flow. Infections were allowed to progress for 12 days, at which time leaf washes from all 12 treatments were obtained by vortexing 6 leaves collected from each of 3 replicate plants in 10 mL autoclaved water. In the experiment where we filter-removed *Albugo*, we filtered leaf washes from control plants through a 6 µm filter (Whatman No. 3 Qualitative, GE Healthcare, Inc.) to ensure that any spores spread by air were not further carried. For treatment by chemical inhibition of *Albugo*, the leaf washes were not treated a second time. Leaf washes were used to reinoculate a second set of plants (4 5-week old plants in a 9x9cm pot). These were treated similarly after inoculation and each pot was individually covered with sterilized Mira cloth (Merck Millipore, Inc). After another 12 days, the epiphytic (leaf surface) and endophytic (intra/intercellular) microbial communities were recovered from collected leaves from each treatment (S9 Fig). The infection experiments were performed in three replicates.

***Growth assays on* A. thaliana *for 1-on-1 interactions of* Dioszegia *sp. with plant-associated bacterial isolates***

Endophytic bacteria and yeast were isolated from wild *Arabidopsis* populations by washing leaves briefly with autoclaved water, followed by 70 % ethanol and 2 % bleach. Subsequently, leaves were washed 3 times with water to remove residuals and afterwards crushed in 10 ml autoclaved water. To select for bacteria and fungi, the solution was plated on King's B (20 g Peptone, 1.5 g K_2_HPO_4_, 1.5 g MgSO_4_ * 7H_2_O, 10 mL glycerol and 15 g agar in 1L water at pH 7.2) and malt extract (20 g malt extract and 20 g agar) media, respectively. Colonies that appeared different were subcultured at least twice on fresh media. A few isolates were from the rhizosphere and were generously provided by Yang Bai [1].

Interaction between *Dioszegia* and bacteria was observed on 4-weeks-old *Arabidopsis thaliana* (Ws-0) seedlings grown under sterile conditions on 1/2 MS media. Bacteria and Dioszegia were grown in liquid 10 % tryptic soy broth [TSB] and potato dextrose [PD] media (200 g diced potatoes boiled in 1L water for 1h, strained with 20g dextrose), respectively, until they reached an OD_600_ of 0.6. The microbes were pelleted, suspended in 10 mM MgCl_2_ and 200 μl were sprayed on the individual plants using an airbrush pistol (Conrad Electronics, Germany). *Dioszegia* was first inoculated followed after two days by one bacterial isolate. Seedlings were 3 weeks old at the time of inoculation. After 1-week, leaf discs (d=0.07 cm^2^) were punched out from single leaves, crunched and suspended in 50 μl of water. The CFU's for bacteria and Dioszegia were determined using 10 % TSB plates containing Nystantin and PDA plates containing antibiotics, respectively. Experiments were performed in three fully independent biological replicates.

***DNA extraction and amplicon library preparation.***

We prepared libraries for 10 samples from each of two wild collection events (always 2 samples from WH, 2 from ERG, 3 from EY, 2 from JUG, and 1 from PFN) and 3 samples of each A. thaliana accession from the garden experiment collected on October 5^th^, 2013. From the controlled lab experiments we prepared libraries for triplicates of each of 12 treatments. In total, libraries were prepared from 65 epiphyte and 65 endophyte leaf fraction samples (130 samples total) (see S1 File for samples and index sequences).

For DNA extractions, collected membranes and leaves that had been stored at -80 ^o^C were manually crushed with a pestle while still frozen in liquid nitrogen. The whole membrane was processed or about 0.2 g of leaf material was used. To tubes containing the crushed samples, acid-washed and sterilized zirconium beads of diameters 0.1 and 0.5 mm (0.2 g each) were added along with 0.5 mL of DNA extraction buffer. The buffer consisted of 0.5% SDS, 50 mM TRIS buffer at pH 8, 200 mM NaCl, 2mM EDTA, 1mg/mL Lysozyme, and 100mg/mL proteinase K. The samples/beads were first incubated for 45 min at 37^o^C, were then beat using a Bertin Precellys 24 (Bertin Technologies, Inc) at 6300 rpm for 2 x 45 seconds with a 15 second pause. A second incubation was then carried out at 37^o^C for 45 minutes in the presence of 10 µg/mL RNAse. The tubes were then centrifuged at 10000 rpm for 2 minutes and the liquid was recovered to a new tube. The nucleic acids were cleaned up with phenol/chloroform/isoamyl alcohol (25:24:1), and chloroform/isoamyl alcohol (24:1), then precipitated by adding 1/10th of the sample volume of 3 M sodium acetate and 2.5x volume 100% ethanol and centrifuging at 4^o^C at 15000 rpm for 40 minutes. The product was washed twice with 70% ethanol. Samples recovered from membranes were eluted in 40 μL TRIS pH 8.0, while leaf samples were eluted into 200 μL. To avoid potential problems with PCR inhibition, samples were mixed 1:1 with 20% Chelex-100 and allowed a 30 minute contact time, after which the liquid fraction was recovered and stored at -20^o^C until further use.

PCR amplification was performed with a custom protocol in two steps to allow the use of blocking primers to decrease plant plastid contamination that occurs with 16S amplification (only used for endophyte samples). In the first step, universal primers (sequences and details in S2 File) [2](Bacteria V3/V4: B341F [2] / B806R [3], Bacteria V5/V6/V7: B799F [4] / B1194R [5], Fungi ITS1: ITS1F [6] / ITS2 [7], Fungi ITS2: fITS7 [8] / ITS4 [7], Oomycete ITS1: ITS1O [9] / 5.8s-O-R (designed for this study), Oomycete ITS2: 5.8s-O-F (designed for this study) / ITS4 [7]) were used to amplify targeted regions. For each sample, all six targeted regions were amplified on a single PCR plate with 20 μL reactions containing 0.2 μL Q5 high-fidelity DNA polymerase (New England Biolabs, Inc), 0.5 μL template, 1X Q5 GC Buffer, 1x Q5 5x reaction buffer, 0.08 μM each forward and reverse primer, 0.25 μM blocking primer (endophyte samples only), 225 μM dNTP and 10.33 μL (endophytes) or 10.83 μL (epiphytes) nuclease free water. Triplicate plates were run in parallel on three independent thermocycler blocks (Bio-Rad Laboratories, Inc.) at 95 °C for 40 sec, 10 cycles (endophytes) or 25 cycles (epiphytes) of 95 °C for 35 sec, 55 °C for 45 sec, 72 °C for 15 sec with a final elongation at 72 °C for 3 min. The three reactions were combined and 10 μL was recovered for an enzymatic cleanup with Antarctic phosphatase and Exonuclease I (New England Biolabs, Inc) to degrade leftover primers and inactivate nucleotides (0.5 μL each enzyme with 1.22 μL Antarctic phosphatase buffer at 37 °C for 30 minutes followed by 80 °C for 15 min). The products of the enzymatic cleanup reaction were then used as template in a second, 50 μL PCR reaction containing 0.5 μL Q5 high-fidelity DNA polymerase (New England Biolabs, Inc), 0.5 μL template (endophyte) or 12.22 μL template (epiphyte), 1x Q5 GC Buffer, 1x Q5 5x reaction buffer, 0.16 μM each forward and reverse primer, 200 μM dNTP and 26.34 μL (endophytes) or 14.14 μL (epiphytes) nuclease free water. The plate was amplified with the same cycling steps as before for 25 cycles (endophyte) or 10 cycles (epiphyte) for a total of 35 cycles. This step elongated amplicons from the first step with primers that consisted of a concatenation of the Illumina adapter P5 (forward) or P7 (reverse), an index sequence (reverse only), a linker region, and the base primer for the region being amplified. We used 20 different reverse primers that were identical except for the 12-bp index [10] that would be used later to identify sequencing products in combined libraries. Information for all primers used can be found in S2 File. Finally, the elongated products were cleaned up and purified using 1.8x volume Ampure XP purification beads (Beckman-Coulter, Inc) and eluted in 40 μL according to manufacturers instructions.

***Preparation and amplicon sequencing on Illumina MiSeq***

Amplified and cleaned gene products were first fluorescently quantified with PicoGreen (Life Technologies, Inc) with salmon sperm DNA (Invitrogen, Inc) as standard in two independent measurements and at both 100x and 1000x dilutions. The results were averaged, and molar DNA concentrations were calculated using the approximate length of the DNA products of each region to correct for the length of the salmon sperm DNA. Products of 120 amplicon libraries (the 6 targeted amplicon regions from epiphyte and endophyte templates from 10 samples) were combined in equimolar concentrations. From the combined product, we cleaned up 400 μL twice using 0.8x volume Ampure XP purification beads and eluted into 40 μL, which eliminated any primer dimers that may have remained in products. Before sequencing, the final product was quantified *via* qPCR using as standard a similar library of amplicons that had already been successfully sequenced (Agilent qPCR NGS Library quantification kit, Agilent Technologies, Inc). The final library was used for loading a MiSeq lane spiked with 15% PhiX genomic DNA to ensure high enough sequence diversity. 3.4 μL of a 100 pM solution of custom sequencing primers complementary to the linker/primer region of the concatenated primers (S2 File) were added together for each of the forward, reverse, and index sequencing reactions. Sequencing was performed for 500 cycles to recover 250 bp of information in the forward and reverse directions.

***Oomycete ITS database creation***

We needed to address the lack of oomycete taxa coverage in fungal ITS databases. Further, conserved regions where ITS primers are designed have some similarity to non-targeted groups, so a broader database is needed to identify non-target amplicons, e.g., amplicons from oomycetes and host plants in fungal ITS amplicon libraries. To address this problem, we created a more diverse eukaryote ITS reference database for use in processing ITS reads. To do so, we combined the UNITE fungal ITS database ([11], Qiime-compatible version, downloaded on July 7^th^, 2014), the *Arabidopsis thaliana* ITS sequence, and a custom made oomycete ITS database. To create the oomcyete database, we wrote a script to recover all nucleotide sequences from NCBI corresponding to taxid 4762 (oomycetes). For each recovered sequence, we then used the GIDs to recover the corresponding taxonomy and created a 6-level taxonomy file (in a QIIME 1.8.0 [12]-compatible format similar to the Greengenes 16S rRNA [13] and UNITE ITS databases). With the sequences, we picked OTUs using the QIIME implementation of UCLUST [14] with default settings at a 97% sequence similarity. To locate the 5.8s sequence in OTUs (most of which contained ITS1, 5.8S rRNA gene, ITS2), sequences were BLASTed [15] against a database of fungal 5.8S rRNA sequences, allowing poor matches (e-value 100). We then used a custom perl script to remove the 5.8s regions keeping sequences at least 80bp long and discarding those containing degenerate bases. We then trimmed the previously made taxonomy files to those sequences that were remaining after all processing steps. With the combined fungal, A. thaliana, and oomycete databases, we used ITSx 1.0.8 [16] to divide the database into ITS1 and ITS2 regions which were used as databases in further analyses (see below).

***Processing amplicon data***

We developed a custom pipeline to simultaneously process reads from bacteria, fungi, and oomycetes for downstream analysis. Between pipeline steps, custom scripts performed tasks like adjusting headers to make files compatible for various softwares. We started by processing each Illumina lane individually up until operational taxonomic unit (OTU) picking. The first step was to demultiplex and quality filter the data using Qiime 1.8.0 [12]. The quality filter truncated reads after three consecutive bases below quality 15 and discarded the read if < 50% of bases were below quality 15. We also allowed up to 2 errors in the barcode (Golay barcodes [10] are distinguishable with up to 3 errors) and up to 5 “N” bases. A custom script then split the resulting sequence files into 6 groups based on which amplicon library they originated from (bacteria 16S V3/V4 or V5/V6/V7, fungi ITS1 or ITS2 and oomycete ITS1 or ITS2) and separated reads that still had a matching pair and reads which were now “orphans” (where the paired read was quality filtered out). All subsequent steps were completed for all 6 amplicon groups. Next, we used Cutadapt 1.2.1 ([17], defaults except for minimum length of 10 after trimming and minimum overlap length of 10) to trim adapter sequences from reads in case short amplicons were completely sequenced through and again recovered matching pairs and “orphan” reads. Next, we aligned paired reads using Pandaseq [18] with defaults except allowing reads to lack a barcode, selecting output to fastq format, allowing min/max aligned lengths of 100 bp/600 bp, and outputting unaligned pairs to a file. Unaligned pairs are those which did not overlap because of long amplicons or because of quality filtering and are output end-to-end. The barcodes of aligned and unaligned pairs from Pandaseq were recovered and the files were re-split with Qiime without any quality filtering to get back Qiime-compatible files. At this point in the pipeline, we had for each of 6 libraries aligned and unaligned reads, as well as read 1 and read 2 orphans from the first quality filtering step.

Some amplicon data processing pipelines discard unpaired reads. We found that especially for ITS amplicon libraries of some microbes, reads are only obtained in either the forward or reverse direction. Discarding unpaired reads causes loss of information associated with these taxa. Thus, to include as many of these reads as possible, for each of the 6 libraries we concatenated all reads together (aligned, unaligned, and orphans). For fungal and oomycete ITS2 reads, we used ITSx 1.0.8 [16] to trim reads to only the ITS region with defaults except that we preserved sequence headers, checked against fungi, oomycete and plant profiles, allowed single domain matching with an e-value cutoff of 1e-5.0, allowed matching of only one HMM gene profile and turned on saving partial ITS regions. This was not performed or necessary for the oomycete ITS1 region because because ITSx relies on detection of neighboring rRNA gene regions which were not large enough for detection here. Next, for all 6 groups, we performed denovo and reference-based chimera checking with USEARCH 6.1 [19] with defaults as implemented in QIIME. We used the greengenes 13_8 release [13] and our custom eukaryote database (above) as a reference for bacteria and fungi/oomcyetes, respectively. Discovered chimeric sequences were removed from the fasta files.

To make it possible to keep forward and reverse “orphan reads”, we first picked “prefix” OTUs then from those OTU representative sequences picked “suffix” OTUs using the prefix/suffix OTU picker in QIIME. This works because all reads after quality filtering were at least 125 bp, and the picker combines reads into OTUs whose first/last (prefix/suffix) 125 bp are identical. For the final OTU picking, data resulting from all Illumina sequencing lanes were combined. Since barcode identifier sequences were reused between lanes, we added a letter to representative sequence headers corresponding to the Illumina lane from which it came before concatenating together matching groups (e.g., bacteria V3/V4 prefix/suffix OTUs from each sequencing run) from 8 Illumina lanes. Next, for all 6 groups we picked final OTUs using UCLUST [14] at 97% identity and picked representative sequences for each OTU. Since these final OTUs were only picked from the suffix OTUs, we used a custom perl script to combine the OTU mapping files from all OTU picking steps. This was necessary so that all original reads represented by a final OTU were accounted for. In the final OTU map, we filtered OTUs with < 2 reads, as is default in QIIME.

With the final set of OTUs, we could now assign taxonomy using the RDP taxonomy assigner [20] implemented in QIIME at a confidence level of 0.8 for 16S sequences and 0.65 for ITS sequences. We used QIIME to generate an OTU table and to filter OTUs present in < 2 samples and with < 50 reads. As a final filtering step, we filtered the following taxa from the 16S OTU tables: “Unclassified” or “Unassigned” reads, and any read in the class “Chloroplast” or the order “Rickettsiales” (primarily host mitochondria 16S). From the ITS OTU tables we filtered: “Unassignable”, “Unclassified”, the kingdom “unidentified” and the kingdom Viridiplantae (host ITS). Because our ITS database contained more than one eukaryotic group, we were able to identify significant amounts of non-target reads in some samples (e.g., oomycetes in fungal ITS libraries). Therefore, we filtered fungi from oomycete ITS OTU tables and oomycetes from fungal ITS OTU tables.

For all downstream analyses (except where noted) the OTU tables were summarized at the genus level and randomly subsampled to an even depth per sample. Because of differences in sequencing depth (endophytes were generally less deeply sequenced because of host-derived amplicons that were filtered out) we subsampled epiphyte and endophyte sample tables separately. Tables were subsampled as deeply as possible while maintaining most samples for analysis. For field samples, depths for epiphytes were 2000, 14000, 3000, 7000, 10000 and 1000 sequences per sample for bacteria V3/V4, bacteria V5/V6/V7, fungal ITS1, fungal ITS2, oomycete ITS1 and oomycete ITS2 amplicons, respectively. Endophytes were rarefied to 900, 7000, 1000, 1000, 1000 and 1000 reads per sample for the same groups, respectively. For analysis of laboratory experiments, subsampling was to 12000, 15000, 32000 and 2000 reads per sample for epiphytes from bacteria V3/V4, bacteria V5/V6/V7, fungal ITS1, fungal ITS2, respectively. For measurements where comparison between all amplicon groups was necessary (for example for alpha diversity in S2 Fig), we rarefied each to 900 sequences per sample. For analyses other than alpha diversity, rarefied tables were log-transformed before analysis.

***Statistical analysis***

Generally, the two amplicon datasets for each group of organisms (e.g., Bacteria V3/V4 and V5/V6/V7) were treated as complementary since some level of primer bias means that no primer set will detect all target organisms. Thus, we performed all analyses on each dataset and used the results of both complementarily. For example, in the network analysis we considered a correlation between two organisms to be true whether it was found with both datasets or with only one dataset. When results from the two datasets were the same and this would affect downstream analyses we only considered one (for example in the correlation network analysis, the degree of the nodes [microorganisms] would be skewed by finding the same correlation multiple times).

*Evaluation of variation between wild and garden experiment samples*

To evaluate how microbial communities varied between sampling locations and sampling times in the Tübingen wild sites, or between host accessions in the Cologne garden experiment, we used constrained correspondence analysis (CCA) in the R package Vegan 2.1-10 [21]. We first used the command cca to build an unconstrained model of the log-transformed microbial genera abundance data, then constrained the model by sampling location, sampling time, or host accession. We visualized the results by plotting the first two unconstrained axes and calculated the fraction of the microbial community variation that was correlated to the constraining variables. To test significance of the correlations, we used the built-in ANOVA function in Vegan which randomly permutes the data classes of the samples. Permutation tests are only of limited usefulness with few data points where permutation is rapidly saturated. Therefore, we supplemented this analysis with Tukey’s HSD analysis (command aov and TukeyHSD in R 3.1.0 [22]) to determine which genera were significantly (p<0.01 for location and sampling time and p<0.05 for host accession) enriched due to one of the factors (i.e., relatively more abundant in one location compared to another, in one season compared to another or on one host accession compared to another).

Alpha diversity estimations based on the number of observed genera were also used to analyze differences between sampling sites. For this analysis, genera-summarized microbial abundance tables were each randomly subsampled 10 times and the number of observed taxa in each subsample was calculated using QIIME 1.8.0 scripts. Depths of subsampling are given above. We calculated the average of the 10 values and the results were visualized in boxplots by sampling site (based on even-depth subsampling) or were stored for use in correlation analysis (see below for correlation analysis, see above for subsampling depths used). We tested for significant differences in alpha diversity using a Welch’s two-sided t-test between locations with the built-in R function pairwise.t.test.

*Analysis of genera distribution*

For samples collected in the wild and from our garden experiment, we were also interested in evaluating and comparing distribution of microbial genera between sites. To make results comparable between groups that were sequenced at different depths, we used genera abundance tables that were subsampled to 1000 sequences per sample (see above). From these, we calculated for each genus how many different sites it was observed in (across the six different sampling locations). Our relatively deep sequencing approach should have the advantage of being able to detect even low-abundance microbes to ascertain whether or not distribution effects are really present. To take advantage of this, we counted a genus as present at a site if any read was found for that genus in any sample from a given site.

*Correlation and network analysis*

We used correlation networks to evaluate potential microbial interactions and to find microbes that are especially interactive. Essentially, we calculated correlations between all taxa in log-transformed abundance tables using the lm function in R 3.1.0 [22]. We did not consider binary interactions where the presence of one microbe would depend absolutely on the presence of the other regardless of abundance because this information would be distorted by pooling leaves. Networks were generated for OTUs grouped taxonomically at each of the order, family, genus or species levels. We did not consider extremely low-abundance taxa by limiting to those with at least 50 observations that were present in more than 10 samples. Correlations were calculated based on all samples from both wild sampling in Tübingen [Experiment 1] and the Cologne garden experiment [Experiment 2]. Strong correlations should be apparent in data subsets, so we also calculated correlations using only data from the wild sampling in Tübingen, and discarded correlations that were not supported by this subset (i.e., correlations with a p-value > 0.05). After calculations, we rejected correlations of organisms to themselves, unless they were from different leaf compartments. To confirm that correlations were well-supported, we randomly subsampled observations in each pair of genera 100 times and counted the number of times where the scaled r-square value (STM x R^2^, see below) was > 3. Correlations to alpha diversity were calculated similarly, except that the correlations were measured between each genus and the observed number of taxa (at maximum subsampling depth, see above). Correlations to diversity were only measured between kingdoms, since many organisms would be expected to be autocorrelated to diversity in the amplicon set from which they are derived.

To get a robust identification of highly interactive “hub” microbes, we used multiple networks calculated based on several cutoffs for “weak” correlations. First, at each taxonomy level, we generated four correlation networks based on discarding “weak” correlations with p-values > 0.1, 0.01, 0.001 or 0.0001. We used multiple p-values because we found that increasingly strong cutoffs did not just reduce the number of discovered hubs, but instead altered the network such that different hubs were found with different cutoffs. We additionally created another metric which accounts for strength of the correlation (R^2^-value) and the distribution of the taxa to avoid correlations based on only a few points. In short, we calculated the ratio of the sum of observations to the maximum observation, where higher values represent genera with broader distribution for which correlations can more reliably be calculated. We averaged these ratios for each taxa, then scaled the r-square value by this amount (creating the STM x R^2^ measurement) and used a value of 3 as a cutoff for a fifth network. To analyze the nodes in the network, we used a custom script to translate correlation data from each taxa level and cutoff level into a format compatible with Cytoscape 3.2.0 [23]. We loaded data into Cytoscape, calculated node statistics (excluding nodes that were not connected to the main interconnected network), and output parameters degree, betweeness centrality and closeness centrality. We also used Cytoscape to fit a power law to the degree distribution. Next, we used R 3.1.0 [22] to fit a normal distribution to log-transformed degree, betweenness centrality or closeness centrality data and to calculate the values above which nodes can be considered outliers, corresponding to p < 0.1. Nodes that were above this value for all three parameters using any of the five correlation cutoffs were considered to be hubs. Fig 2 is a representation of this process with genus-level taxonomy using the STM x R^2^ > 3 cutoff, where three nodes were identified as “hubs”. For this figure, visualizations of inter-genera correlations was prepared in Cytoscape 3.2.0. S11Fig shows the analysis for all cutoffs resulting in 6 robust genus-level hubs.

The three hub nodes in Fig 2 were highly supported at different taxa levels, so we output their first neighbors in the correlation network and computed shared neighbors. To evaluate the “keystone-ness” of these three hubs, we checked the dependence of edges in the network to these and other specific organisms. To do so, we re-calculated the correlation network using the function pcor.test in the R package ppcor 1.0 to evaluate partial correlations between all genera while controlling for the abundance of the test organism. For test organism abundances, we log-transformed the abundances from each of the two amplicon data sets, scaled the values from 0-1 so that we could average them, and used the averaged abundances in the analysis. We then calculated how many of the correlations in the original network (using the network with cutoff STM x R^2^ > 3) were supported in the network calculated with partial correlations. Edges “dependent” on the organism of interest were those that were no longer supported.

*Laboratory experimental analysis*

To find out how epiphytic microbial communities changed with different treatments in laboratory experiments, we looked at alpha and beta diversity measurements based on class and genus-level microbial abundances. Therefore, OTU abundance tables were summarized not only at the genus level but also at the class level in QIIME 1.8.0 [12]. Epiphyte abundance tables were randomly subsampled to depths of 12000, 15000, 32000 and 2000 sequences per sample for bacteria V3/V4, bacteria V5/V6/V7, fungi ITS1 and fungi ITS2 regions, respectively. For each abundance table we calculated alpha diversity analogously to the method described above for other samples. In short, alpha diversity for each sample was the average number of observed taxa based on 10 random subsamples of abundance tables. We also generated a sample distance matrix based on the Chi-square distance between samples. We wrote a script in R 3.1.0 [22] to recover from the matrix distances within replicates (referred to here as treatment replicability) and between treatments. Alpha diversities and distances were visualized in boxplots with significance based on a Welch’s two-sided t-test between groups (tested using the built-in R function pairwise.t.test). Significance was only tested within the *A. laibachii* or *A. candida* treatments, not between the two microbes.

Endophyte reads from control laboratory samples (plants not infected with *Albugo* sp.) were largely dominated by plant plastid 16S or host ITS sequences, while infected plants contained higher levels of microbial-derived reads. Previous work has linked the levels of microbial compared to host reads from amplicon sequencing with levels of microbial colonization [24]. To check if we could also estimate endophytic colonization this way, we checked rarefaction curves of alpha diversity (observed taxa) in endophyte samples. Essentially, samples were rarefied to various depths (10 times per depth) and the average number of observed taxa were plotted vs. depth (S24 Fig). The rarefaction curves near an asymptote, which means we have nearly completely sampled endophyte communities and microbial endophyte reads should be a good estimate of endophyte colonizaiton. We then used a Welch’s two-sided t-test to check for significant (p < 0.05) differences between treatments for total endophyte colonization and colonization of individual genera. We included genera as significant if the colonization was significantly higher in infected host leaves but not in any control compared to other controls.

*Factor correlation overlap*

We again turned to constrained correspondence analysis in the R package Vegan 2.0-10 to find overlaps in the microbial community variation correlated to the confirmed hub microbe *Albugo* sp., the putative hub microbe *Dioszegia* sp. and to the factors sampling location and sampling time in wild collected samples. For this analysis, we focused on bacteria since they were most strongly affected by abundances of the two eukaryote hub microbes. To ensure we had reliable abundance data for the hub microbes corresponding to each sample, we considered the abundances generated from both the ITS1 and ITS2 datasets. In short, we log-transformed their abundances and scaled these values from 0-1, then averaged the abundances of the two datasets for each sample. Next, we performed constrained correspondence analysis using each of the bacterial dataset. Constraints used in the analysis were each of *Albugo* sp*.* abundance, *Dioszegia* sp. abundance, and the factors sampling location and sampling time together. We also tested all combinations of the constraints and used the version of ANOVA in Vegan to test for independence of the factors with p < 0.05. We calculated the “total model” as the amount of bacterial community variation that was constrained using all variables together. We also calculated the “overlap percent” which is the percentage of the variation correlated to location and sampling time which could also be correlated to either *Albugo* sp. or *Dioszegia* sp. (“factor overlap” divided by location/sampling time correlated variation, where “factor overlap” is the percent of total community variation shared by *Albugo*/*Dioszegia* and location/sampling time).

**A. thaliana *and* A. laibachii *genetic diversity measurement***

Microsatellite markers were used to analyze genetic diversity between wild *A. thaliana* plants and wild/common garden experiment *A. laibachii* strains. For the host plant, three sets of previously designed primers were used [25]: nga59F/R, nga111F/R, nga158F/R. For *A. laibachii*, we used Gmato [26] to identify >600 candidate genomic regions where small sequence repeats occur. We narrowed this list to 13 candidate genomic regions with > 5 motif repeats and where coverage in 5 sequenced *A. laibachii* isolates was identified as variable. We next designed primers flanking these candidate regions and selected three primer sets (AlSSR2[F/R]: tgtgacgctcgacatgctat/atttcccgtttgagcagttg, AlSSR6 [F/R]: aatataacccttgcctccgttt/aaagagtggctttggacgaa, AlSSR10 [F/R]: cactgaattcgacacacgatct/ttgaattcggtttatgatgtgc) that could easily distinguish four lab strains of *A. laibachii* but were not sensitive to *A. candida*. Primers specific for the ITS region of *A. candida* (Can[F/R]: ttgtgactgggacgttgtact/agccgaagcaaaacataccgc) were used to check for its presence in the same samples.

All PCRs for plant and pathogen diversity estimations were performed using extracted DNA from endophytic compartment samples. DNA concentrations in samples were measured relative to one another using PicoGreen and equivalent amounts of DNA were added in a volume of 0.5 µL to the reaction. The PCRs to check microsatellite marker polymorphisms was performed in a 20 µL reaction using 0.2 µL Phusion Polymerase (New England Biolabs, Inc.) with 1x Phusion high fidelity buffer, 0.1 µM forward and reverse primers, 100 µM dNTP and nuclease free water. The PCR program consisted of: 98 ^o^C for 30 sec, then 30 cycles of 98 ^o^C for 10 sec, 55 ^o^C for 30 sec, 72 ^o^C for 30 sec, followed by a final extension at 72 ^o^C for 5 min. Products were visualized on a 3% high-resolution agarose gel (Bio-Budget) with a 25bp ladder for the *A. thaliana* markers and a 100bp ladder for *Albugo* markers. In some gels weak bands appeared in the background at lengths unexpected for the used markers. These were considered as non-target amplification and only bright bands of similar intensity were analyzed. If the length of amplified bands for all three markers were indistinguishable, we considered strains to be the same. The PCRs to check for *A. candida* presence/absence was performed in a 20 µL reaction using 0.2 µL Taq Polymerase (New England Biolabs, Inc.) with 1x standard Taq buffer, 0.1 µM forward and reverse primers, 100 µM dNTP and nuclease free water. The PCR program consisted of: 95 ^o^C for 2 min, then 30 cycles of 95 ^o^C for 30 sec, 58 ^o^C for 40 sec, 72 ^o^C for 1 min, followed by a final extension at 72 ^o^C for 3 min. 10 µL of product was run out on a 2% agarose gel and visualized to check for amplification products.

***qPCR quantification of endophytic* Albugo *sp.***

To calculate the amount of endophytic *Albugo* sp., total oomycetes were measured relative to the host by quantifying amounts of the oomycete 5.8S rRNA gene and *A. thaliana* EF1-alpha gene in each endophytic sample. First the total DNA was roughly quantified using a nonodrop and diluted to a concentration of 1 ng/μL. We used 5 μL of this dilution in 15 μL reactions with 2x Sso Advanced universal Sybr Green Supermix (BioRad Laboratories, LLC). To each reaction we added 0.2 μM of each oomycete primer (oomy5.8sF: ACTTTCAGCAGTGGATGTCTA / oomy5.8sR: GATGACTCACTGAATTCTGCA) or each A. thaliana primer (AT_EF1a_F_q: AAGGAGGCTGCTGAGATGAA / AT_EF1a_R_q: TGGTGGTCTCGAACTTCCAG). For quantification of samples collected from laboratory experiments with *Albugo* sp., all 12 samples of each replicate were quantified together using the *Albugo laibachii* Nc14-infected Col-0 sample from each replicate as standard. For all samples from the Cologne garden experiment or Tübingen samples, the *Albugo laibachii* Nc14-infected Col-0 sample from replicate 3 of the lab experiment was always used as standard. The measured amount of oomycete 5.8S rRNA gene were divided by the amount of host EF1-alpha gene. To calculate the endophytic Albugo level, the oomycete measurements were then multiplied by the relative abundance of the genus *Albugo.* For each sample, we used the average *Albugo* relative abundance from the ITS1 and ITS2 gene regions. If a relative abundance value was only available from one dataset we used that value only.

***Fluourescent cell counting of epiphytic bacteria***

Plants were either sprayed with *A. laibachii* Nc14 or *A. candida* Nc2 spore solutions (4 x 10^4^ conidiospores per plant), sterile H_2_O, or spore solutions with Metalaxyl and Benalaxyl which inhibit oomycete growth (179 µM resp. 4.7 µM end concentration). At 12 days post infection [dpi] all leaves of one plant were harvested. Pictures of all leaves were taken to measure the total leaf surface. Bacteria were washed off via 10 minutes agitation in a 50 ml tube with 1% Triton X-100 in 1 x PBS buffer. To separate spores from bacteria the solution was filtered through a 5 µm syringe filter. A 2 ml subsample of this bacterial solution was stained with 25 µl of a 1.4 mM DAPI solution for 5 minutes and filtered through a 0.2 µm non-fluorescing polycarbonate filter (Whatman, Inc.) supported with a glass fiber filter. The filter was afterwards rinsed with 5 ml 1 x PBS buffer and mounted on a microscope slide. For bacterial cell counting 10 pictures per sample were randomly taken with the 40x objective of a Zeiss Axio Imager.D2 microscope (DAPI reflector, UV filter). The ImageJ ITCN plugin was used for automatic cell counting of each picture. Based on these measurements, the number of bacteria per mm² leaf was calculated.

**References**

1. Bai Y, Müller DB, Srinivas G, Garrido-Oter R, Potthoff E, Rott M, et al. Functional overlap and specialization of the Arabidopsis leaf and root microbiotas. In revision. 2015.

2. Muyzer G, de Waal EC, Uitterlinden AG. Profiling of complex microbial populations by denaturing gradient gel electrophoresis analysis of polymerase chain reaction-amplified genes coding for 16S rRNA. Appl Environ Microbiol. 1993;59(3):695-700. PubMed PMID: 7683183; PubMed Central PMCID: PMC202176.

3. Caporaso JG, Lauber CL, Walters WA, Berg-Lyons D, Lozupone CA, Turnbaugh PJ, et al. Global patterns of 16S rRNA diversity at a depth of millions of sequences per sample. Proc Natl Acad Sci U S A. 2011;108 Suppl 1:4516-22. doi: 10.1073/pnas.1000080107. PubMed PMID: 20534432; PubMed Central PMCID: PMC3063599.

4. Chelius MK, Triplett EW. The diversity of archaea and bacteria in association with the roots of *Zea mays* L. Microb Ecol. 2001;41(3):252-63. doi: 10.1007/s002480000087. PubMed PMID: 11391463.

5. Bodenhausen N, Horton MW, Bergelson J. Bacterial communities associated with the leaves and the roots of *Arabidopsis thaliana*. PLoS One. 2013;8(2):e56329. doi: 10.1371/journal.pone.0056329.

6. Gardes M, Bruns TD. ITS primers with enhanced specificity for basidiomycetes--application to identification of mycorrhizae and rusts. Mol Ecol. 1993;2(2):113-8. PubMed PMID: IND 93054055; PubMed Central PMCID: PMCM07/0002.

7. White TJ, Bruns T, Lee S, Taylor J. Amplification and direct sequencing of fungal ribosomal RNA genes for phylogenetics. In: Innis MA, Gelfand DH, Sninsky JJ, White TJ, editors. PCR Protocols: a guide to methods and applications. New York, USA: Academic Press; 1990. p. 315-22.

8. Ihrmark K, Bodeker IT, Cruz-Martinez K, Friberg H, Kubartova A, Schenck J, et al. New primers to amplify the fungal ITS2 region--evaluation by 454-sequencing of artificial and natural communities. FEMS Microbiol Ecol. 2012;82(3):666-77. doi: 10.1111/j.1574-6941.2012.01437.x. PubMed PMID: 22738186.

9. Thines M, Zipper R, Schauffele D, Spring O. Characteristics of *Pustula tragopogonis* (syn. *Albugo tragopogonis*) newly occurring on cultivated sunflower in Germany. J Phytopathol. 2006;154(2):88-92. doi: DOI 10.1111/j.1439-0434.2006.01065.x. PubMed PMID: WOS:000234975400005.

10. Caporaso JG, Lauber CL, Walters WA, Berg-Lyons D, Huntley J, Fierer N, et al. Ultra-high-throughput microbial community analysis on the Illumina HiSeq and MiSeq platforms. ISME J. 2012;6(8):1621-4. Epub 2012/03/10. doi: 10.1038/ismej.2012.8. PubMed PMID: 22402401; PubMed Central PMCID: PMC3400413.

11. Kõljalg U, Nilsson RH, Abarenkov K, Tedersoo L, Taylor AFS, Bahram M, et al. Towards a unified paradigm for sequence-based identification of fungi. Mol Ecol. 2013;22(21):5271-7. doi: 10.1111/mec.12481. PubMed PMID: 24112409.

12. Caporaso JG, Kuczynski J, Stombaugh J, Bittinger K, Bushman FD, Costello EK, et al. QIIME allows analysis of high-throughput community sequencing data. Nat Methods. 2010;7(5):335-6. Epub 2010/04/13. doi: 10.1038/nmeth.f.303. PubMed PMID: 20383131; PubMed Central PMCID: PMC3156573.

13. DeSantis TZ, Hugenholtz P, Larsen N, Rojas M, Brodie EL, Keller K, et al. Greengenes, a chimera-checked 16S rRNA gene database and workbench compatible with ARB. Appl Environ Microbiol. 2006;72(7):5069-72. doi: 10.1128/AEM.03006-05. PubMed PMID: 16820507; PubMed Central PMCID: PMC1489311.

14. Edgar RC. Search and clustering orders of magnitude faster than BLAST. Bioinformatics. 2010;26(19):2460-1. doi: 10.1093/bioinformatics/btq461. PubMed PMID: 20709691.

15. Altschul SF, Gish W, Miller W, Myers EW, Lipman DJ. Basic local alignment search tool. J Mol Biol. 1990;215(3):403-10.

16. Bengtsson-Palme J, Ryberg M, Hartmann M, Branco S, Wang Z, Godhe A, et al. Improved software detection and extraction of ITS1 and ITS2 from ribosomal ITS sequences of fungi and other eukaryotes for analysis of environmental sequencing data. Methods Ecol Evol. 2013;4(10):914-9. doi: Doi 10.1111/2041-210x.12073. PubMed PMID: WOS:000325459600003.

17. Martin M. Cutadapt removes adapter sequences from high-throughput sequencing reads. EMBnetjournal; Vol 17, No 1: Next Generation Sequencing Data Analysis. 2011.

18. Masella AP, Bartram AK, Truszkowski JM, Brown DG, Neufeld JD. PANDAseq: paired-end assembler for illumina sequences. BMC Bioinform. 2012;13:31. doi: 10.1186/1471-2105-13-31. PubMed PMID: 22333067; PubMed Central PMCID: PMC3471323.

19. Edgar RC, Haas BJ, Clemente JC, Quince C, Knight R. UCHIME improves sensitivity and speed of chimera detection. Bioinformatics. 2011;27(16):2194-200. doi: 10.1093/bioinformatics/btr381. PubMed PMID: 21700674; PubMed Central PMCID: PMC3150044.

20. Wang Q, Garrity GM, Tiedje JM, Cole JR. Naive Bayesian classifier for rapid assignment of rRNA sequences into the new bacterial taxonomy. Appl Environ Microbiol. 2007;73(16):5261-7. doi: 10.1128/AEM.00062-07. PubMed PMID: 17586664; PubMed Central PMCID: PMC1950982.

21. Oksanen J, Blanchet FG, Kindt R, Legendre P, Minchin PR, O'Hara RB, et al. vegan: Community Ecology Package version 2.0-10. 2013. Available from: <http://CRAN.R-project.org/package=vegan>.

22. Team RDC. R: A language and environment for statistical computing Vienna, Austria: R Foundation for Statistical Computing; 2011. Available from: <http://www.R-project.org/>.

23. Shannon P, Markiel A, Ozier O, Baliga NS, Wang JT, Ramage D, et al. Cytoscape: a software environment for integrated models of biomolecular interaction networks. Genome Res. 2003;13(11):2498-504. doi: 10.1101/gr.1239303. PubMed PMID: 14597658; PubMed Central PMCID: PMC403769.

24. Edwards J, Johnson C, Santos-Medellín C, Lurie E, Podishetty NK, Bhatnagar S, et al. Structure, variation, and assembly of the root-associated microbiomes of rice. Proc Natl Acad Sci U S A. 2015;10.1073/pnas.1414592112. doi: 10.1073/pnas.1414592112.

25. van Treuren R, Kuittinen H, Kärkkäinen K, Baena-Gonzalez E, Savolainen O. Evolution of microsatellites in *Arabis petraea* and *Arabis lyrata*, outcrossing relatives of *Arabidopsis thaliana*. Mol Biol Evol. 1997;14(3):220-9. PubMed PMID: 9066790.

26. Wang X, Lu P, Luo Z. GMATo: A novel tool for the identification and analysis of microsatellites in large genomes. Bioinformation. 2013;9(10):541-4. doi: 10.6026/97320630009541. PubMed PMID: 23861572; PubMed Central PMCID: PMC3705631.
